# Agreement Between Mega-Trials and Smaller Trials: A Systematic Review and Meta-Research Analysis

**DOI:** 10.1001/jamanetworkopen.2024.32296

**Published:** 2024-09-06

**Authors:** Lum Kastrati, Hamidreza Raeisi-Dehkordi, Erand Llanaj, Hugo G. Quezada-Pinedo, Farnaz Khatami, Noushin Sadat Ahanchi, Adea Llane, Renald Meçani, Taulant Muka, John P. A. Ioannidis

**Affiliations:** 1 Meta-Research Innovation Center at Stanford (METRICS), Stanford University, Stanford, California; 2 Institute of Social and Preventive Medicine (ISPM), University of Bern, Bern, Switzerland; 3 Graduate School for Health Sciences, University of Bern, Bern, Switzerland; 4 Department of Diabetes, Endocrinology, Nutritional Medicine and Metabolism, Inselspital, Bern University Hospital, University of Bern, Switzerland; 5 Department of Global Public Health and Bioethics, Julius Center for Health Sciences and Primary Care, University Medical Center Utrecht, Utrecht University, Utrecht, the Netherlands; 6 Epistudia, Bern, Switzerland; 7 Department of Molecular Epidemiology, German Institute of Human Nutrition Potsdam-Rehbrücke, Nuthetal, Germany; 8 German Centre for Diabetes Research (DZD), München-Neuherberg, Germany; 9 Department of Population Health Sciences, Duke University School of Medicine, Durham, North Carolina; 10 Community Medicine Department, Tehran University of Medical Sciences, Tehran, Iran; 11 Department of Internal Medicine, Lausanne University Hospital, University of Lausanne, Lausanne, Switzerland; 12 Division of Endocrinology and Diabetology, Department of Internal Medicine, Medical University of Graz, Graz, Austria; 13 Stanford Prevention Research Center, Department of Medicine, Stanford University School of Medicine, Stanford, California; 14 Department of Epidemiology and Population Health, Stanford University School of Medicine, Stanford, California; 15 Department of Biomedical Data Science, Stanford University School of Medicine, Stanford, California; 16 Department of Statistics, Stanford University School of Humanities and Sciences, Stanford, California

## Abstract

**Importance:**

Mega-trials can provide large-scale evidence on important questions.

**Objective:**

To explore how the results of mega-trials compare with the meta-analysis results of trials with smaller sample sizes.

**Data Sources:**

ClinicalTrials.gov was searched for mega-trials until January 2023. PubMed was searched until June 2023 for meta-analyses incorporating the results of the eligible mega-trials.

**Study Selection:**

Mega-trials were eligible if they were noncluster nonvaccine randomized clinical trials, had a sample size over 10 000, and had a peer-reviewed meta-analysis publication presenting results for the primary outcome of the mega-trials and/or all-cause mortality.

**Data Extraction and Synthesis:**

For each selected meta-analysis, we extracted results of smaller trials and mega-trials included in the summary effect estimate and combined them separately using random effects. These estimates were used to calculate the ratio of odds ratios (ROR) between mega-trials and smaller trials in each meta-analysis. Next, the RORs were combined using random effects. Risk of bias was extracted for each trial included in our analyses (or when not available, assessed only for mega-trials). Data analysis was conducted from January to June 2024.

**Main Outcomes and Measures:**

The main outcomes were the summary ROR for the primary outcome and all-cause mortality between mega-trials and smaller trials. Sensitivity analyses were performed with respect to the year of publication, masking, weight, type of intervention, and specialty.

**Results:**

Of 120 mega-trials identified, 41 showed a significant result for the primary outcome and 22 showed a significant result for all-cause mortality. In 35 comparisons of primary outcomes (including 85 point estimates from 69 unique mega-trials and 272 point estimates from smaller trials) and 26 comparisons of all-cause mortality (including 70 point estimates from 65 unique mega-trials and 267 point estimates from smaller trials), no difference existed between the outcomes of the mega-trials and smaller trials for primary outcome (ROR, 1.00; 95% CI, 0.97-1.04) nor for all-cause mortality (ROR, 1.00; 95% CI, 0.97-1.04). For the primary outcomes, smaller trials published before the mega-trials had more favorable results than the mega-trials (ROR, 1.05; 95% CI, 1.01-1.10) and subsequent smaller trials published after the mega-trials (ROR, 1.10; 95% CI, 1.04-1.18).

**Conclusions and Relevance:**

In this meta-research analysis, meta-analyses of smaller studies showed overall comparable results with mega-trials, but smaller trials published before the mega-trials gave more favorable results than mega-trials. These findings suggest that mega-trials need to be performed more often given the relative low number of mega-trials found, their low significant rates, and the fact that smaller trials published prior to mega-trial report more beneficial results than mega-trials and subsequent smaller trials.

## Key Points

**Question:**

Are the results of mega-trials with 10 000 participants or more similar to meta-analysis of trials with smaller sample sizes for the primary outcome and/or all-cause mortality?

**Findings:**

In this meta-research analysis of 82 mega-trials, meta-analyses of smaller studies showed overall comparable results with mega-trials, but smaller trials published before the mega-trials gave more favorable results than mega-trials. There were very low rates of significant results for the primary outcome and all-cause mortality for mega-trials.

**Meaning:**

The findings of this study suggest that mega-trials need to be performed more often, given the relative low number of mega-trials found, their low significant rates, and the fact that smaller trials published prior to mega-trial reported more beneficial results than mega-trials and subsequent smaller trials.

## Teaser

This meta-analysis compares results of mega-trials with results from meta-analyses of smaller trials for the primary outcome and all-cause mortality.

## Introduction

Most randomized comparisons of interventions in medicine use small to modest sample sizes. The call for more mega-trials (ie, large sample trials) with over 10 000 participants has been longstanding.^1,2^ Mega-trials have been rare, but there has been a renewed interest recently. Several mega-trials have found that certain interventions, like vitamin D supplementation, may not be as effective as previously thought.^3,4^ Conversely, other mega-trials, such as the Second International Study of Infarct Survival (ISIS-2) Collaborative Group trial on streptokinase and aspirin after myocardial infarction^5^ found favorable results with major clinical impact. Conducting mega-trials may be facilitated by the growth of interest in pragmatic (ie, practical) research,^6,7^ new platforms for recruitment of participants,^8^ and wider recognition of the limitations of small trials. Therefore, it is important to understand and compare the results of mega-trials with those of smaller trials.

Meta-analyses rarely include large trials, and small trials have traditionally been considered more susceptible to biases, including more prominent selective reporting.^9,10^ Previous literature comparing results of meta-analyses of small trials with subsequent large trials has shown heterogeneous results.^11,12,13,14,15,16^ Furthermore, different methods have been proposed to analyze the agreement.^17^ Different event rates in the control group of the considered trials (baseline risk), differences in trial quality, and variable susceptibility to bias of the health outcomes under investigation may also generate heterogeneity.^11^ Moreover, mega-trials and smaller trials may have topic- and question-specific biases that are different in the 2 groups. In previous work, there was also no clear consensus on what constitutes a large trial. Some^18^ have considered the amount of evidence in each trial (inverse of variance or sample size) as a continuum, while others tried to separate trials with sufficient power (eg, 80%) to detect plausible effects,^19^ and yet others used arbitrary sample size thresholds, (eg, 1000 participants).^12,14^ To our knowledge, no comprehensive empirical examination has systematically compared the results of mega-trials with sample sizes exceeding 10 000 participants versus smaller trials.

Here, we aimed to systematically identify such mega-trials, identify which ones have been included in meta-analyses for their primary outcomes and/or for mortality outcomes, compare the results of these mega-trials against the combined results of smaller trials, and identify potential factors associated with discrepancies.

## Methods

### Design and Eligibility Criteria for Mega-Trials

This meta-analysis was a meta-research project; because this study is not a typical meta-analysis, we followed the Preferred Reporting Items for Systematic Reviews and Meta-analyses (PRISMA) reporting guideline where applicable.^18^ The original protocol was registered in Open Science Framework Because the information we used consisted of publicly available results of RCTs, and not patient-specific data, there was no need for ethical review. We analyzed meta-analyses of clinical trials that have included mega-trial results in their analysis for calculations of a summary effect size for the primary end point of the mega-trial. Additionally, we considered data on all-cause mortality as a secondary outcome because it is the most severe and objective outcome.

Mega-trials were considered for analysis if they were noncluster, nonvaccine randomized clinical trials (RCTs) regardless of masking; had a sample size of more than 10 000 participants; had a peer-reviewed publication presenting the results of the primary end point; and were included in a meta-analysis for their primary outcome and/or all-cause mortality. We excluded cluster trials because the effective sample size is much smaller than the number of participants. We excluded vaccine trials because very large vaccine trials usually have different considerations and types of outcomes than mega-trials of other interventions.

For a meta-analysis to be included in the analysis, it had to have a systematic review design and include the results of the mega-trial along with any number of other trials in obtaining summary effect size estimates with the effect size and variance data available (or possible to calculate) for each trial from presented information.

### Search Strategy

We searched for mega-trials in ClinicalTrials.gov (last updated January 2023) and then performed PubMed searches (until June 2023) to identify the most recent meta-analyses that included the results of these mega-trials for the primary outcome of the mega-trial and for all-cause mortality. Details on the search process are in eAppendix 1 in Supplement 1.

### Data Extraction

For each selected meta-analysis, we extracted the results of RCTs included in the summary effect size estimate that incorporated the effect size estimate of the mega-trial. We also extracted information, whenever available, on the risk of bias assessments for each included trial based on Cochrane Risk of Bias Tools (original, revised, and version 2). All data extractions (except mega-trial identification) were performed by 2 reviewers (L.K. and H.R.D.; L.K. and H.G.Q.P.; L.K. and E.L.L.; L.K. and N.S.A.; L.K. and F.K.; L.K. and R.M.; and L.K. and A.L.L.), and differences were settled by discussion. For any unsettled discrepancies, a third senior reviewer (T.M.) was invited to arbitrate. Details on data extraction appear in eAppendix 2 in Supplement 1.

### Amendments to the Original Protocol

Some of the eligible meta-analyses contained results from other mega-trials that had not been detected by our search. Therefore, we described these extra identified trials and included them in our analyses. We extracted information for all mega-trials based on whether they found statistically significant or nonsignificant results and whether they were designed to show noninferiority. In several meta-analyses, some trials did not pass the 10 000-participant threshold but were substantially large enough to blur the effects. Therefore, in a sensitivity analysis, we compared the results of mega-trials vs only the smaller trials that weighted less than one-fifth of the least weighted mega-trial; in another sensitivity analysis, we compared the results of mega-trials vs smaller trials that weighted less than one-tenth of the least weighted mega-trial. We then further restricted these trials to those published only before or up to the first trial. We also explored the agreement on different thresholds, setting the threshold at a sample size of 30 000. In addition, we also compared the agreement between the mega-trials, when more than one was included in a meta-analysis. Finally, we also assessed the risk of bias for the mega-trials that had not been assessed (or had been assessed using various non-Cochrane tools [eg, Jadad scale]) using the Cochrane Risk-of-Bias Tool.^25^

### Statistical Analysis

In each eligible meta-analysis, we combined the results from non–mega-trials using random effects (and fixed effects as sensitivity analysis) and compared them against the results of the mega-trial. In meta-analyses where several mega-trials were available, the results of the mega trials were combined using random effects first before being compared against the results of smaller trials. Any cluster trials were considered to be non–mega-trials.^20^

The odds ratio (OR) was the metric of choice. All the analyzed outcomes were dichotomous. Between-trial heterogeneity assessments used τ^2^ between-study variance estimator, *Q* test, and *I^2^* statistics.^21^

We obtained the log ratio of ORs (ROR) and its variance (the sum of the variances of the logOR in the 2 groups) between the mega-trials and the smaller trials for each eligible outcome. Then, the logROR estimates were combined across each outcome using the DerSimonian-Laird random-effects calculations.^22^ We also performed sensitivity analyses using the Hartung-Knapp-Sidik-Jonkman (HKSJ) method.^23^ In all calculations, treatment effects in single trials and meta-analyses thereof were coined consistently so that an ROR less than 1 means a more favorable outcome for the intervention group over the control group.

A sensitivity analysis was performed to assess whether the results were different when non–mega-trials were included in the calculations only if they were published up until (and including) the year of publication of any mega-trials and comparing them with the results of the mega-trial. This analysis more specifically targets the research question of whether mega-trials corroborate the results of smaller trials that have been performed before them. A separate analysis also compared the results of non–mega-trials published up until the year of publication of the mega-trial vs non–mega-trials published subsequently.

Separate subgroup analyses were performed for the comparison of results in mega-trials vs other trials according to masking (open-label vs masked), intervention type, specialty (eg, cardiovascular), and per heterogeneity (low vs non-low) of the mega-trials. We also performed exploratory meta-regressions considering the same variables (masking, type of outcome, type of intervention, and specialty) and also risk of bias in the mega-trials (high vs other), risk of bias in the other trials (proportion at high risk), median number of participants in non–mega-trials, and total number of participants in non–mega-trials. We also performed exploratory tests for small study effect sizes (Egger test),^24^ when there were more than 10 trials.

Analyses were conducted using Stata software version 17 (StataCorp). The threshold for significance was a 2-tailed P < .05. Data analysis occurred from January to June 2024.

## Results

### Identification of Mega-Trials and the Respective Meta-Analyses

**Figure 1.**
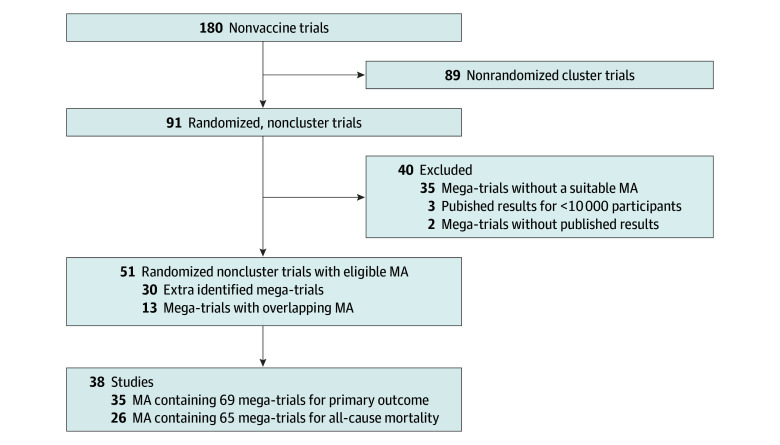
Flowchart of Mega-Trial Selection MA indicates meta-analysis.

A total of 180 registered completed phase 3 or 4 mega-trials that did not involve vaccines and that had 10 000 or more participants were identified through our search (Figure 1). Among these, 91 were randomized, noncluster, nonvaccine mega-trials; but 35 of these 91 trials lacked an appropriate meta-analysis and 2 had no published results, leaving 51 mega-trials with an eligible meta-analysis for either primary outcome and/or all-cause mortality. Three trials registered with more than 10 000 participants and had eligible meta-analyses; however, they randomized less 10 000 participants and were excluded by our analyses.^26,27,28^ Results were compared to smaller trials across 58 meta-analyses, including 35 for primary outcome^29,30,31,32,33,34,35,36,37,38,39,40,41,42,43,44,45,46,47,48,49,50,51,52,53,54,55,56,57,58,59,60,61,62,63,64,65,66,67,68,69,70,71,72,73,74,75,152^ and 26 for all-cause mortality.^29,32,33,34,35,37,38,39,40,41,42,43,44,45,46,47,49,50,51,52,53,54,56,57,58,59,60,61,62,64,65,66,67,68,69,70,72,73,74,76,77,78^ In 3 studies,^32,41,68^ all-cause mortality was the mega-trial’s primary outcome (Table 1). For 19 mega-trials that had a composite primary outcome^,^^30,32,33,39,42,45,46,48,53,55,56,59,61,62,66,68,69,71,152^ no eligible meta-analysis was identified for the complete composite outcome, therefore the meta-analysis of one of the subsets of the composite outcome with the highest number of events was analyzed (Table 1 and eTable 1, eAppendix 3, and eTable 4 in Supplement 1).

**Table 1.** General Characteristics of the Included Mega-Trials

Meta-analysis		Mega-trial	Intervention	Control	Meta-analyzed PO	Mega-trial results		Risk of bias
PO	ACM					PO, OR (95% CI)	ACM, OR (95% CI)	
Bonney et al,^113^ 2022	Bonney et al,^113^ 2022	Aberle et al,^29^ 2011^a^	Low dose computed tomography	Usual care or x-ray	Lung cancer incidence	0.80 (0.70-0.92)	0.94 (0.87-0.99)	High
Chi et al,^115^ 2016	Chi et al,^115^ 2016	Jamerson et al,^53^ 2008^b^	ACEi, ARBs, and CCB	Other combinations	Fatal and nonfatal stroke	0.83 (0.65-1.08)	0.89 (0.75-1.07)	Low
Li et al,^124^ 2016	Wang et al,^145^ 2019	Huo et al,^51^ 2015	Enalapril and Folic Acid	Enalapril	Stroke	0.79 (0.67-0.92)	0.94 (0.8-1.11)	Low
Yu et al,^135^ 2022	Yu et al,^135^ 2022	Bosch et al,^38^ 2012	ω-3	Placebo	Cardiovascular mortality	0.98 (0.87-1.1)	0.98 (0.88-1.08)	Low
Tsigkas et al,^132^ 2023	Tsigkas et al,^132^ 2023	Vranckx et al,^68^ 2018^a^^,^^b^	Very short duration of antiplatelet therapy	>3 mo antiplatelet therapy	All-cause mortality	0.82 (0.64-1.06)	0.82 (0.64-1.06)	High
Hasebe et al,^121^ 2023	Sardar et al,^143^ 2015	Gerstein et al,^47^ 2008^a^	Intensive glucose-lowering treatment	Conventional treatment	MACE	0.94 (0.81-1.1)	1.28 (1.06-1.54)	High
Khan et al,^123^ 2006	NA	Pepine et al,^63^ 2003	β-blockers	Other drugs	MACE	1.02 (0.94 -1.11)	0.96 (0.09-1.00)	Low
Yu et al,^135^ 2022	Yu et al,^135^ 2022	Yokoyama et al,^73^ 2007^a^	ω-3 and statin	ω-6, placebo, or usual care	MACE	0.8 (0.68-0.94)	1.08 (0.91-1.28)	High
Wang et al,^133^ 2022	Wang et al,^133^ 2022	Ikeda et al,^52^ 2014	Low dose aspirin	Placebo or no aspirin	MACE	0.93 (0.76-1.14)	0.98 (0.83-1.15)	High
Keum et al,^122^ 2022	Keum et al,^122^ 2022	Manson et al,^57^ 2019^a^	Vitamin D	Control (placebo or other supplements)	Total cancer incidence	0.96 (0.87- 1.06)	0.98 (0.86-1.11)	Low
Gencer et al,^120^ 2021	NA	Albert et al,^31^ 2021	ω-3 and vitamin D supplementation	Placebo	Atrial fibrillation	1.09 (0.96-1.25)	No Data	Low
Cheng et al,^114^ 2021	Cheng et al,^114^ 2021	Nissen et al,^61^ 2016^b^^,^^c^	Celocoxib	Other NSAID	Myocardial infarction	0.89 (0.65-1.2)	0.69 (0.5-0.94)	Low
Tharmaratnam et al,^131^ 2021	Wanas et al,^144^ 2020	Yusuf et al,^74^ 2008^c^	Blood pressure lowering treatment	Placebo or alternative regimen	Stroke	0.94 (0.85-1.04)	1.02 (0.92-1.14)	Low
Singh et al,^130^ 2009	Singh et al,^130^ 2009	Antman et al,^32^ 2006^b^	Enoxaparin	Unfractioned heparin	All-cause mortality	0.92 (0.82-1.01)	0.92 (0.82-1.01)	Low
Alkhalil et al,^111^ 2021	Ennezat et al,^140^ 2023	Jukema et al,^54^ 2019	Intensive lipid lowering therapy	Less intensive lipid lowering therapy	MACE	0.85 (0.78-0.94)	0.91 (0.82-1.02)	Low
Dong et al,^117^ 2022	NA	Zampieri et al,^75^ 2021	IV fluid treatment with balanced solution	IV normal solution	90 d survival	0.96 (0.88-1.05)	No Data	Low
Maagaard et al,^126^ 2022	Maagaard et al,^127^ 2020	Fox et al,^45^ 2008^b^	Ivabradine	Placebo	MACE	0.87 (0.80-0.95)	1.04 (0.82-1.18)	Low
Yang et al,^134^ 2022	Yang et al,^134^ 2022	Hercberg et al,^49^ 2007	β-carotene supplementation	Placebo	MACE	0.96 (0.76-1.24)	0.77 (0.57-1.04)	Low
Schandelmaier et al,^129^ 2017	NA	Landray et al,^55^ 2004^b^	Extended-release niacin with laropiprant	Laropiprant or matching placebo	Any revascularization procedure	0.9 (0.82-0.99)	1.09 (0.99-1.21)	Low
NA	Ennezat et al,^140^ 2023	Ridker et al,^77^ 2007	Bococizumab	Placebo	MACE	0.83 (0.67-1.01)	1.02 (0.79-1.31)	Low
Chiavaroli et al,^116^ 2021	Riaz et al,^142^ 2019	Barter et al,^33^ 2007^b^	HDL modifiers	CETP	Myocardial infarction	1.2 (0.94-1.54)	1.58 (1.14-2.19)	Low
Bae et al,^112^ 2016	Bae et al,^112^ 2016	Wallentin et al,^69^ 2009^b^	Ticagrelor	Clopidogrel	Myocardial infarction	0.82 (0.75-0.91)	0.77 (0.68-0.89)	Low
Hasebe et al,^121^ 2023	Rados et al,^141^ 2020	Scirica et al,^67^ 2013^c^	DPP-4i	Placebo	MACE	0.99 (0.88-1.12)	1.11 (0.96-1.27)	Low
Hasebe et al,^121^ 2023	Ali et al,^139^ 2024	Bhatt et al,^35^ 2021^c^	SGLT2-I	Placebo	MACE	0.76 (0.65-0.88)	1.00 (0.83-1.2)	Low
Niu et al,^128^ 2022	Niu et al^128^ 2022	White et al,^70^ 2017^b^	Anti-inflamatory drugs	Placebo	MACE	0.93 (0.84-1.03)	1.00 (0.9-1.13)	Low
Hasebe et al,^121^ 2023	Rados et al^141^ 2020	Holman et al,^50^ 2017^c^	GLP-1 RA	Placebo	MACE	0.92 (0.84-1.02)	0.84 (0.74-0.95)	Low
Zhuo et al,^138^ 2018	Zhuo et al,^138^ 2018	Mehta et al,^59^ 2010^b^	Double dose clopidogrel	Other antiplatelet regimens	Cardiac mortality	0.96 (0.77-1.20)	0.94 (0.76-1.16)	Low
Duncan et al,^118^ 2018	Duncan et al,^118^ 2018	Devereaux et al,^42^ 2014^b^	Clonidine	Placebo or non-α-2 adrenergic agonists	Myocardial infarction	1.12 (0.95-1.32)	1.01 (0.71-1.43)	Low
Fanari et al,^119^ 2017	Fanari et al,^119^ 2017	Bhatt et al,^34^ 2006	DAPT (≥12 mo)	Dual antiplatelet therapy (6-12 mo)	MACE	0.92 (0.82-1.04)	0.99 (0.85-1.14)	Low
Liang et al,^125^ 2021	NA	Giugliano et al,^48^ 2003^b^^,^^c^	Edoxaban	Warfarin	Stroke	0.88 (0.75-1.04)	0.87 (0.79-0.96)	Low
Yuan et al,^136^ 2018	Yuan et al,^136^ 2018	Mega et al,^58^ 2012	Rivaroxaban	Placebo	MACE	0.82 (0.72-0.94)	0.79 (0.65-0.98)	Low
Zhang et al,^137^ 2021	NA	Bohula et al,^36^ 2018^c^	Antiobesity drugs	Placebo	MACE	0.99 (0.86-1.14)	1.08 (0.89-1.31)	Low
Niu et al,^128^ 2022	Niu et al,^128^ 2022	O’Donoghue et al,^62^ 2014^b^	Anti-inflamatory drugs	Placebo	Myocardial infarction	0.97 (0.86-1.10)	0.93 (0.81-1.08)	Low
Albasri et al,^110^ 2021	Wanas et al,^144^ 2020	Yusuf et al,^152^ 2008^b^^,^^c^	ARB	Placebo or standard care	Cardiovascular mortality	1.03 (0.92-1.16)	0.97 (0.89-1.07)	High
Maagaard et al,^127^ 2020	Maagaard et al,^127^ 2020	Fox et al,^44^ 2014	Ivabradine	Placebo	Myocardial infarction	1.05 (0.91-1.22)	1.06 (0.93-1.21)	High
Safi et al,^153^ 2019	Safi et al,^153^ 2019	Chen et al,^41^ 2005	β-blockers	Placebo	All-cause mortality	0.98 (0.92-1.05)	0.98 (0.92-1.05)	Low
Chiavaroli et al,^116^ 2021	Riaz et al,^142^ 2019	Bowman et al,^39^ 2017^b^	HDL Cholesterol modifiers	Placebo	Myocardial infarction	0.86 (0.77-0.96)	0.97 (0.89-1.05)	Low
NA	Ennezat et al,^140^ 2023	Ridker et al,^76^ 2008	Intensive LDL-c reducing therapy	Less Intensive LDL-c reducing therapy	MACE	0.56 (0.46-0.69)	0.80 (0.66-0.96)	Low
Wang et al,^133^ 2022	Wang et al,^133^ 2022	Gaziano et al,^46^ 2018^b^	Low dose aspirin	Placebo or no aspirin	MACE	0.95 (0.78-1.15)	0.99 (0.8-1.25)	Low
Chiavaroli et al,^116^ 2021	Riaz et al,^142^ 2019	Lincoff et al,^56^ 2017^b^	HDL modifiers	Placebo	Myocardial infarction	1.00 (0.84-1.2)	0.84 (0.7-1.00)	Low
Chiavaroli et al,^116^ 2021	Riaz et al,^142^ 2019	Schwartz et al,^66^ 2012^b^	HDL modifiers	Placebo	Myocardial infarction	1.01 (0.88-1.17)	0.98 (0.82-1.12)	Low
Hasebe et al,^121^ 2023	Ali et al,^139^ 2024	Wiviott et al,^72^ 2019^c^	SGLT2-I	Placebo	MACE	0.94 (0.84-1.04)	0.92 (0.82-1.04)	High
Hasebe et al,^121^ 2023	Ali et al,^139^ 2024	Neal et al,^60^ 2017^c^	SGLT2-I	Placebo	MACE	0.85 (0.74-0.97)	0.89 (0.75-1.04)	Low
Niu et al,^128^ 2022	Niu et al,^128^ 2022	Ridker et al,^64^ 2017	Anti-inflamatory drugs	Placebo	MACE	0.87 (0.77-0.98)	0.92 (0.81-1.06)	Low
Yu et al,^135^ 2022	Yu et al,^135^ 2022	Roncaglioni et al,^65^ 2013	ω-3 and statin	ω-6, placebo, or usual care	MACE	0.99 (0.89-1.1)	1.03 (0.89-1.21)	Low
Fanari et al,^119^ 2017	Fanari et al,^119^ 2017	Bonaca et al,^37^ 2015^a^	DAPT (≥12 mo)	Dual antiplatelet therapy (6-12 mo)	MACE	0.83 (0.75-0.93)	0.94 (0.82-1.08)	Low
Yuan et al,^136^ 2018	Yuan et al,^136^ 2018	Eikelboom et al,^43^ 2017	Rivaroxaban and aspirin	Aspirin	MACE	0.75 (0.65-0.86)	0.82 (0.7-0.95)	Low
Bae et al,^112^ 2016	NA	Wiviott et al,^71^ 2007^b^	Prasugrel	Clopidogrel	Myocardial infarction	0.75 (0.66-0.84)	0.94 (0.78-1.15)	Low
NA	Wanas et al,^144^ 2020	Yusuf et al,^78^ 2016	Rosuvastatin	Placebo	MACE	0.76 (0.65-0.9)	0.93 (0.8-1.08)	Low
Wang et al,^133^ 2022	Wang et al,^133^ 2022	Bowman et al,^40^ 2018	Aspirin	Placebo	MACE	0.87 (0.78-0.98)	0.93 (0.84-1.04)	Low
Bae et al,^112^ 2016	NA	Abtan et al,^30^ 2016^b^	Cangrelor	Clopidogrel	MACE	0.80 (0.67-0.97)	0.72 (0.35-1.48)	Low

Abbreviations: ACEi, angiotenzin converting enzyme inhibitor; ACM, all-cause mortality; ARBs, angiotenzing receptor blockers; CETP, cholesteryl ester transfer protein; DAPT, dual antiplatelet therapy; GLP-1 RA, glucagon-like peptide-1 receptor agonist; HDL, high density lipoprotein; IV, intravenous; LDL-c, low density lipoprotein cholesterol; MACE, major adverse cardiovascular event; NSAID, nonsteroidal anti-inflamatory drugs; OR, odds ratio; PO, primary outcome; SGLT2-I, sodium glucose cotransporter-2 inhibitors.

^a^ Open label trials; all the other trials were blinded.

^b^ The trial had a composite primary outcome but has been meta-analyzed for only 1 subset of it. Information on the composite outcome results can be found in eTable 1 in Supplement 1.

^c^ Designed for proving noninferiority.

The eligible meta-analyses included estimates from another 30 mega-trials^79,80,81,82,83,84,85,86,87,88,89,90,91,92,93,94,95,96,97,98,99,100,101,102,103,104,105,106,107,108^ that had randomized, noncluster design and more than 10 000 participants but had not been identified in our searches (eTable 2 in Supplement 1). Of these 30 studies, 26 were not registered in ClinicalTrials.gov,^79,80,81,82,83,84,86,87,88,89,90,91,92,93,94,96,97,98,99,100,101,103,104,105,106,107^ while 2^85,108^ had no listed location in ClinicalTrials.gov, 1^95^ had listed no results in ClinicalTrials.gov, and for 1 study,^102^ no reason for missingness was identified. These 30 trials with their estimates for primary outcomes (20 trials) and all-cause mortality (22 trials) were considered in the mega-trials group in all calculations. The meta-analyses included an additional 1 mega-trial that had initially been identified by our search but had no eligible meta-analysis for the primary outcome and/or all-cause mortality but was meta-analyzed for another outcome.^109^ In total, 82 mega-trials were included across all meta-analyses for the primary outcome (69 mega-trials^29,30,31,32,33,34,35,36,37,38,39,40,41,42,43,44,45,46,47,48,49,50,51,52,53,54,55,56,57,58,59,60,61,62,63,64,65,66,67,68,69,70,71,72,73,74,75,79,80,84,85,86,87,89,90,91,92,93,94,97,98,99,100,102,103,104,108,109,152^) and all-cause mortality (65 mega-trials^29,32,33,34,35,37,38,39,40,41,42,43,44,45,46,47,49,50,51,52,53,54,56,57,58,59,60,61,62,64,65,66,67,69,70,72,73,74,76,77,78,79,80,81,82,83,85,87,88,89,92,93,94,95,96,99,101,102,103,104,105,106,107,109,152^).

### Characteristics of Mega-Trials

Of the 82 mega-trials^29,30,31,32,33,34,35,36,37,38,39,40,41,42,43,44,45,46,47,48,49,50,51,52,53,54,55,56,57,58,59,60,61,62,63,64,65,66,67,68,69,70,71,72,73,74,75,76,77,78,79,80,81,82,83,84,85,86,87,88,89,90,91,92,93,94,95,96,97,98,99,100,101,102,103,104,105,106,107,108,109,152^ included in our analyses, 64^30,31,33,34,35,36,37,38,39,40,42,43,44,45,46,47,48,49,50,51,52,53,54,55,56,57,58,59,60,61,62,63,64,65,66,67,68,69,70,71,72,73,74,76,77,78,79,80,81,82,83,84,85,86,89,90,91,92,93,94,96,97,98,100,102,103,104,105,106,108,109^ investigated cardiovascular outcomes, 17 mega-trials^31,38,49,57,65,73,80,88,93,95,97,98,100,101,107,108,109^ were centered around nutritional interventions, and 1 mega-trial^75^ covered various other medical interventions intervention types, such as pharmacological treatment (Table 1 and eTable 1 and eTable 2 in Supplement 1). Moreover, 15 of the mega-trials were open-label,^29,37,47,57,68,73,79,80,81,86,87,90,102,105,106^ while the remaining 65 mega-trials were double-blinded, and 2 trials employed varying degrees of masking (Table 1). Of all the mega-trials, 14^29,44,47,52,68,72,73,79,81,87,97,102,106,152^were judged at high risk of bias. A total of 32 mega-trials^29,30,35,37,39,40,43,45,51,54,55,58,60,64,69,71,73,76,78,79,80,82,85,87,90,92,96,101,102,105,106^ had statistically significant results at *P* < .05 for the primary outcome (30 favoring the intervention group) and only 17^29,33,43,47,48,50,58,61,69,76,79,80,82,86,99,101,106^ had statistically significant results at *P* < .05 for all-cause mortality (13 favoring the intervention group) (Table 1 and eTable 1 and eTable 2 in Supplement 1).

### Comparisons of Mega-Trials vs Smaller Trials: Primary Outcome

A total of 35 comparisons of mega-trials vs other trials were available,^110,111,112,113,114,115,116,117,118,119,120,121,122,123,124,125,126,127,128,129,130,131,132,133,134,135,136,137,138^ yielding a total of 85 point estimates coming from 69 unique mega-trials.^29,30,31,32,33,34,35,36,37,38,39,40,41,42,43,44,45,46,47,48,49,50,51,52,53,54,55,56,57,58,59,60,61,62,64,65,66,67,68,69,70,71,72,73,74,75,76,77,78,79,80,81,82,83,84,85,86,87,88,89,90,91,92,93,94,95,96,97,98,99,100,101,102,103,104,105,106,109,152^ These 69 mega-trials yielded a median (IQR) of 15 715 (12 530-20 114) participants (Table 2). The total number of smaller trials across these 35 mega-trials was 272 (median [range], 6 [1-45] smaller trials) (Table 2). There was a median (IQR) of 1639 (297-4128) participants across the 35 studies from the smaller trials. Of the 272 smaller trials, 133 were published before or up to the year of the first mega-trial of the respective topic. In 7 meta-analyses,^110,114,117,121,124,132,137^ the cumulative sample size of all the other smaller trials exceeded the cumulative sample size of the mega-trials (Table 2).

**Table 2.** Comparison of Results of Meta-Analyses of Mega-Trials and Other Smaller Trials for Primary Outcome

Meta-analysis	Primary outcome	Mega-trials			Other smaller trials			Meta-analysis results, OR (95% CI)	
		No.	Participants, No.	Participants, range per trial	No.	Participants, No.	Participants, range per trial	Mega-trials	Other smaller trials
Bonney et al,^113^ 2022	Lung cancer incidence	2	53 454	15 789-53 454	6	21 879	2450-4104	0.78 (0.70-0.87)	0.80 (0.67-0.95)
Chi et al,^115^ 2016	Fatal and nonfatal stroke	1	11 394	11 394	2	7260	2182-5078	0.84 (0.65-1.08)	0.82 (0.55-1.24)
Li et al,^124^ 2016	Stroke	2	32 766	12 064-20 702	19	46 035	88-8164	0.89 (0.70-1.14)	0.83 (0.73-0.94)
Yu et al,^135^ 2022	Cardiovascular mortality	6	96 361	11 324-25 871	13	31 321	102-8179	0.92 (0.85-1.00)	0.89 (0.76-1.04)
Tsigkas et al,^132^ 2023	All-cause mortality	1	15 968	15 968	7	25 236	1460-7119	0.82 (0.64-1.06)	0.92 (0.73-1.16)
Hasebe et al,^121^ 2023	MACE	2	21 391	10 251-11 140	2	5658	1791-3867	0.94 (0.85-1.03)	0.84 (0.71-0.99)
Khan et al,^123^ 2006	MACE	5	140 693	10 881-79 775	7	36 351	758-9193	0.99 (0.95-1.02)	1.10 (0.99-1.22)
Yu et al,^135^ 2022	MACE	6	96 361	12 505-25 871	16	35 237	101-8179	0.94 (0.89-1.00)	0.92 (0.82-1.04)
Wang et al,^133^ 2022	MACE	6	119 670	12 546-39 876	4	15 287	2539-5713	0.89 (0.84-0.95)	0.82 (0.71-0.94)
Keum et al,^122^ 2022	Total cancer incidence	2	62 223	25 871-36 352	10	27 503	511-5292	0.98 (0.92-1.04)	0.99 (0.89-1.10)
Gencer et al,^120^ 2021	Atrial fibrillation	4	66 182	12 505-25 119	3	14 773	759-8179	1.27 (1.06-1.52)	1.27 (1.02-1.58)
Cheng et al,^114^ 2021	Myocardial infarction	1	23 895	23 895	5	24 359	213-8065	0.89 (0.65-1.22)	1.29 (0.96-1.73)
Tharmaratnam et al,^131^ 2021	Stroke	1	20 332	20 332	6	18 264	1022-6105	0.94 (0.85-1.04)	0.90 (0.76-1.08)
Singh et al,^130^ 2009	All-cause mortality	1	20 479	20 479	6	7093	242-4078	0.92 (0.82-1.02)	0.94 (0.77-1.15)
Alkhalil et al,^111^ 2021	MACE	3	52 687	16 204-19 113	4	29 155	5401-9395	0.90 (0.84-0.96)	0.83 (0.73-0.96)
Dong et al,^117^ 2022^a^	90 d survival	1	10 520	10 520	6	23 997	46-15 802*	0.96 (0.88-1.05)	0.95 (0.88-1.02)
Maagaard et al,^126^ 2022^b^	MACE	1	10 917	10 917	14	7826	19-6505	1.08 (0.94-1.23)	0.89 (0.78-1.01)
Yang et al,^134^ 2022	MACE	5	109 438	12 741-39 876	5	10 970	181-8171	1.02 (0.96-1.08)	1.07 (0.97-1.18)
Schandelmaier et al,^129^ 2017	Any revascularization procedure	1	25 673	25 673	6	3841	64-3365	0.89 (0.81-0.99)	0.99 (0.79-1.23)
Chiavaroli et al,^116^ 2021	Myocardial infarction	4	73 479	12 092-30 449	5	4805	472-1612	0.99 (0.87-1.12)	0.86 (0.50-1.50)
Bae et al,^112^ 2016^b^	Myocardial infarction	3	43 163	10 929-18 624	6	36 097	612-7754	0.79 (0.73-0.86)	0.95 (0.86-1.06)
Hasebe et al,^121^ 2023	MACE	2	131 163	14 671-16 492	3	16 551	4192-6979	1.00 (0.92-1.08)	1.00 (0.90-1.11)
Hasebe et al,^121^ 2023	MACE	3	37 886	10 142-17 160	3	19 659	4401-8238	0.91 (0.77-1.06)	0.88 (0.77-1.01)
Niu et al,^128^ 2022	MACE	3	38 915	10 061-15 828	2	10 308	4786-5522	0.93 (0.87-1.00)	0.86 (0.61-1.21)
Hasebe et al,^121^ 2023	MACE	1	14 752	14 752	8	49 484	3183-9901	0.92 (0.84-1.02)	0.87 (0.78-0.96)
Zhuo et al,^138^ 2018	Cardiac mortality	1	17 263	17 263	3	4015	106-3755	0.96 (0.77-1.20)	1.15 (0.49-2.65)
Duncan et al,^118^ 2018	Myocardial infarction	1	10 010	10 010	8	3898	24-1897	1.12 (0.95-1.35)	0.71 (0.40-1.26)
Fanari et al,^119^ 2017	MACE	2	36 765	15 603-21 162	4	18 826	1850-9961	0.88 (0.8-0.97)	0.84 (0.69-1.01)
Liang et al,^125^ 2021	Stroke	2	28 141	14 070-14 071	4	3612	484-2149	1.01 (0.78-1.30)	0.43 (0.16-1.13)
Yuan et al,^136^ 2018	MACE	2	33 620	15 342-18 278	2	6428	3391-3037	0.79 (0.72-0.87)	0.92 (0.73-1.17)
Zhang et al,^137^ 2021^c^	MACE	1	12 000	11 988	10	18 396	422-8910	0.99 (0.86-1.14)	1.00 (0.78-1.26)
Niu et al,^128^ 2022	Myocardial infarction	3	38 915	10 061-15 828	6	16 721	249-5522	0.91 (0.83-0.98)	0.81 (0.64-1.02)
Albasri et al,^110^ 2021	Cardiovascular mortality	2	29 823	12 705-17 118	19	93 337	530-9794	1.00 (0.90-1.12)	0.90 (0.82-0.98)
Maagaard et al,^127^ 2020	Myocardial infarction	2	30 009	10 907-19 102	3	1801	98-1277	1.03 (0.90-1.17)	0.98 (0.23-4.20)
Safi et al,^153^ 2019	All-cause mortality	2	61 879	16 027-45 852	45	19 202	39-5778	0.85 (0.74-0.97)	0.87 (0.77-0.99)

Abbreviation: MACE, major adverse cardiovascular event.

^a^ One mega-trial was clustered and therefore was accounted for as a smaller trial.

^b^ Comparisons with significant differences between mega-trials and smaller trials.

^c^ No information on the sample size of 1 of the smaller trials.

Detailed information with forest plots on all of the 35 meta-analyses^110,111,112,113,114,115,116,117,118,119,120,121,122,123,124,125,126,127,128,129,130,131,132,133,134,135,136,137,138^ appears in eAppendix 4 in Supplement 1. In the summary analysis, there was no noteworthy discrepancy observed between the results of the mega-trials and those of smaller trials (summary ROR, 1.00; 95% CI, 0.97-1.04; *I^2^* = 0.0; *P* for heterogeneity = .48) (eFigure 1 in Supplement 1). There were 2 instances when disagreement between the mega-trials and the respective smaller trials was beyond chance; the first^112^ was comparing ivabradine with placebo for major adverse cardiovascular event (ROR, 1.21; 95% CI, 1.00-1.47), and the second^126^ was a comparison of new adenosine diphosphate receptor agonist with clopidogrel for myocardial infarction (ROR, 0.83; 95% CI, 0.73-0.95).^,^

### Comparisons of Mega-Trials vs Smaller Trials: All-Cause Mortality

A total of 26 comparisons of mega-trials vs other trials were available.^112,113,114,115,118,119,122,127,128,130,133,134,136,138,139,140,141,142,143,144,145^ and 70 estimates coming from 65 unique mega-trials^29,32,33,34,35,37,38,39,40,41,42,43,44,45,46,47,49,50,51,52,53,54,56,57,58,59,60,61,62,64,65,66,67,69,70,72,73,74,76,77,78,79,80,81,82,83,85,87,88,89,92,93,94,95,96,99,101,102,103,104,105,106,107,109,152^ were considered in these comparisons (Table 3). The median (IQR) total number of participants in all of the mega-trials was 15 919 (12 524-18 857).

**Table 3.** Comparison of Results of Meta-Analyses of Mega-Trials and Smaller Trials for All-Cause Mortality

Meta-analysis	Mega-trials			Other smaller trials			Meta-analysis results, OR (LCI-UCI)	
	No.	Participants, No.	Participants, range per trial	No.	Participants, No.	Participants, range per trial	Mega-trials	Other smaller trials
Ennezat et al,^140^ 2023	2	30 573	12 705-17 802	14	51 672	505-9270	0.80 (0.75-1.01)	0.90 (0.82-0.99)
Chi et al,^115^ 2016	1	11 506	11 506	2	7340	2199-5141	0.89 (0.75-1.07)	0.89 (0.67-1.19)
Wang et al,^145^ 2019	1	20 702	20 702	3	5351	553-3090	0.94 (0.80-1.11)	1.03 (0.74-1.42)
Yu et al,^135^ 2022	5	80 889	12 513-25 871	18	35 548	101-8179	0.97 (0.92-1.03)	0.96 (0.86-1.07)
Bonney et al,^113^ 2022	2	43 501	15 970-27 531	6	22 119	2509-4143	0.95 (0.90-1.00)	0.93 (0.85-1.02)
Wang et al,^133^ 2022	6	120 270	12 546-39 876	4	15 287	2539-5713	0.99 (0.92-1.06)	0.90 (0.78-1.04)
Sardar et al,^143^ 2015	2	21 391	10 251-11 140	10	13 576	43-5238	1.08 (0.79-1.48)	0.99 (0.89-1.11)
Keum et al,^122^ 2022	2	31 105	18 177-12 928	4	6488	1015-2650	0.94 (0.87-1.02)	0.94 (0.84-1.04)
Riaz et al,^142^ 2019	4	72 479	11 092-30 449	4	3834	130-1612	1.02 (0.91-1.14)	1.25 (0.65-2.39)
Cheng et al,^114^ 2021	1	239 553	239 553	4	24 248	916-8067	0.69 (0.50-0.94)	0.90 (0.61-1.35)
Singh et al,^130^ 2009^a^	1	20 479	20 479	6	7093	242-4078	0.92 (0.82-1.02)	0.94 (0.77-1.15)
Ennezat et al,^140^ 2023	12	181 434	10 001-27 564	47	140 831	250-9270	0.93 (0.88-0.98)	0.90 (0.85-0.95)
Maagaard et al,^127^ 2020	2	30 019	10 917-19 102	13	3408	19-1277	1.05 (0.96-1.15)	0.68 (0.35-1.34)
Safi et al,^153^ 2019^a^	2	61 879	16 027-45 852	45	19 202	39-5778	0.85 (0.74-0.97)	0.87 (0.77-0.99)
Fanari et al,^119^ 2017	2	36 765	15 603-21 162	4	18 798	1822-9961	0.97 (0.87-1.07)	1.2 (0.98-1.47)
Niu et al^128^ 2022^b^	3	38 915	10 061-15 828	7	21 476	249-5522	0.96 (0.89-1.04)	1.21 (1.02-1.45)
Rados et al^141^ 2020	1	14 932	14 932	8	41 963	355-9901	0.84 (0.74-0.95)	0.89 (0.82-0.96)
Duncan et al,^118^ 2018	1	10 010	10 010	12	4071	20-1897	1.01 (0.72-1.44)	0.80 (0.56-1.13)
Zhuo et al,^138^ 2018	1	17 263	17 263	2	4831	1076-3755	0.94 (0.76-1.17)	1.06 (0.49-2.30)
Wanas et al,^144^ 2020	4	65 400	12 705-20 332	32	94 942	80-9794	1.00 (0.95-1.06)	1.01 (0.95-1.07)
Bae et al,^112^ 2016	1	h18 624	18 624	1	661	661	0.77 (0.67-0.88)	1.72 (0.50-5.96)
Rados et al,^141^ 2020	2	31 163	14 671-16 492	5	17 119	91-6979	1.08 (0.97-1.19)	0.95 (0.84-1.07)
Ali et al,^139^ 2024	3	37 886	10 142-17 160	8	42 188	1222-8246	0.93 (0.85-1.01)	0.84(0.76-0.93)
Yang et al,^134^ 2022	5	81 816	11 550-22 071	1	840	840	1.08 (0.99-1.18)	1.11 (0.79-1.58)
Yuan et al,^136^ 2018	2	33 602	15 342-18 278	1	3037	3037	0.81 (0.72-0.92)	0.96 (0.53-1.72)
Tsigkas et al,^132^ 2023^a^	1	15 968	15 698	7	25 236	1460-7119	0.82 (0.64-1.06)	0.92 (0.73-1.16)

Abbreviations: LCI, lower confidence interval; OR, odds ratio; UCI, upper confidence interval.

^a^ All-cause mortality was the primary outcome.

^b^ Comparison with significant differences between mega-trials and smaller trials.

The total number of smaller trials in these 26 meta-analyses was 268 (median [range] per meta-analysis, 6 [1-47] smaller trials). There was a median (IQR) of 1132 (250-4038) participants from smaller trials. Of the 268 smaller trials, 117 were published before or up to the year of the first mega-trial of the respective topic. In 5 meta-analyses,^132,139,140,141,144^ the cumulative number of participants in the other smaller trials exceeded the total number of participants in the mega-trials (Table 3). Comprehensive details and forest plots about the 26 meta-analyses appear in eAppendix 5 in Supplement 1.

In the summary analysis, no difference existed between the outcomes of the mega-trials and those of the smaller trials (summary ROR, 1.00; 95% CI, 0.97-1.04; *I^2^* = 0.0%; *P* for heterogeneity = .60) (eFigure 2 in Supplement 1). In one instance testing effects of anti-inflammatory vs placebo in patients with coronary artery diseases,^128^ the results differed beyond chance between mega-trials and the other smaller trials (ROR, 0.79; 95% CI, 0.65-0.97), with mega-trial showing no effect but meta-analysis of smaller trials showing an increased risk.

### Sensitivity Analyses

**Figure 2.**
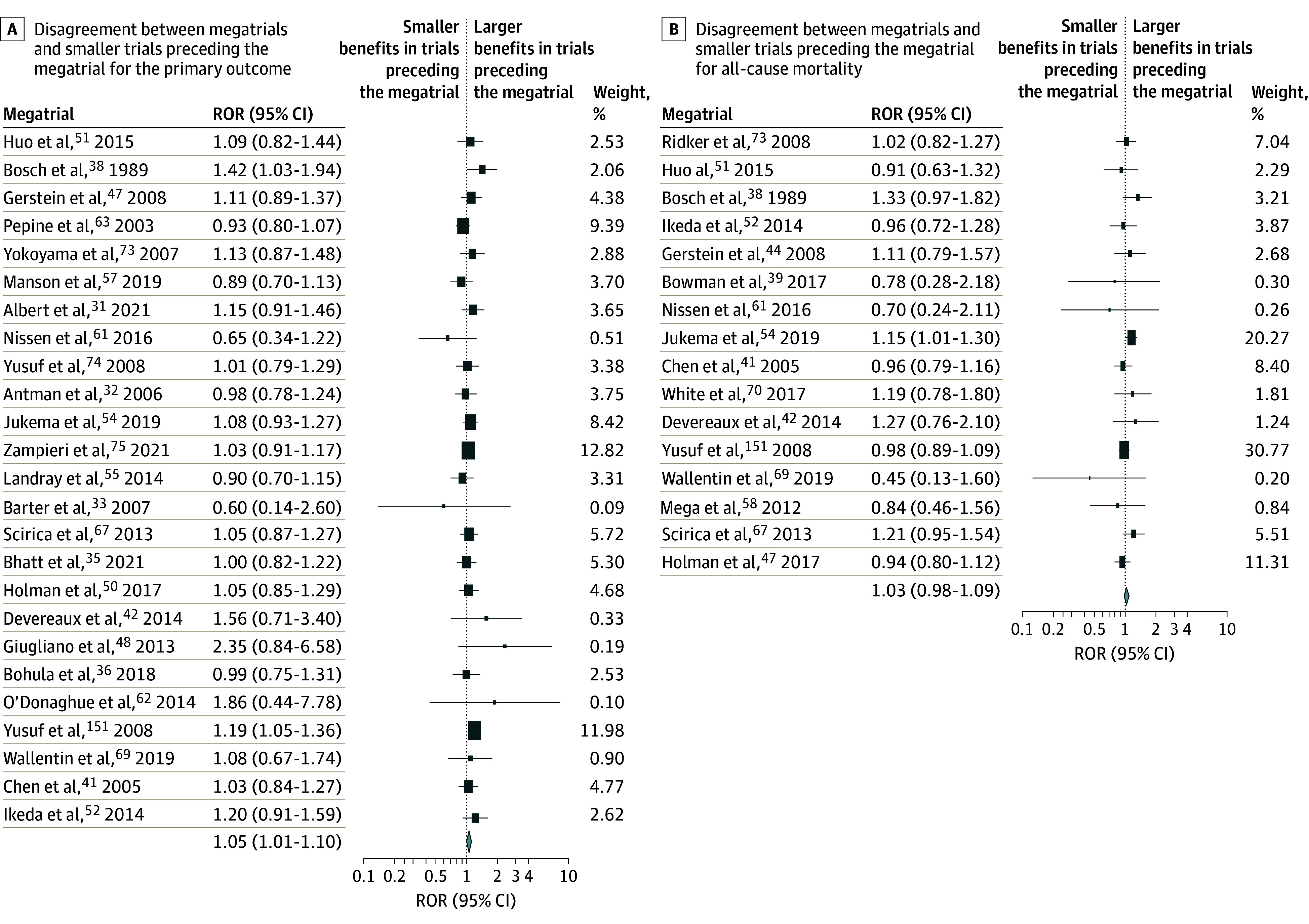
Disagreement Between Mega-Trials and Smaller Trials Preceding the Mega-Trial ROR indicates ratio of odds ratio.

Smaller trials showed significantly larger effects for the primary outcome when compared with mega-trials when they were published before the first megatrial (ROR, 1.05; 95% CI, 1.01-1.10), and similar direction but nonsignificant effect for all-cause mortality (ROR, 1.03; 95% CI, 0.98-1.09) (Figure 2, A and B). Results of smaller trials published before the mega-trial showed significantly higher benefits as compared with smaller trials published subsequently for primary outcome (ROR, 1.10; 95% CI, 1.04-1.18) and similar outcomes for all-cause mortality (ROR, 1.06; 95% CI, 0.98-1.15) (eFigure 3 in Supplement 1).

No difference was seen when results were pooled using fixed effects, having a threshold of 30 000 participants using HKSJ random effects. Other subgroup analyses and meta-regressions were also nonrevealing (eTable 3 and eFigures 4-13 in Supplement 1). No small-study effects were found for the meta-analyses for the primary outcome and 1 meta-analysis^140^ had a significant small-study effects result for all-cause mortality.

### Significance and Noninferiority Across All Mega-Trials

In total, we analyzed and/or described the results from 120 mega-trials. Of the 120, 41 showed a significant result for the primary outcome (33 of which favored intervention over control) and 22 showed a significant result for all-cause mortality ( and 18 of which favored intervention over control). For the 17 studies with noninferiority designs, 15 had reached noninferiority and 2 had significantly better results in the experimental group vs the control group for the primary outcome (Table 1 and eTable 1 and eTable 2 in Supplement 1).

## Discussion

Overall, this meta-analysis of mega-trials found that outcomes from meta-analyses of other smaller clinical trials aligned on average with those of mega-trials in the clinical studies that we examined. This finding could be partly explained by the relatively large sample size of the smaller trials. However, mega-trials tended to have less favorable results than the smaller trials that preceded them timewise, and smaller trials published after the mega-trials tended to have less favorable results than the smaller trials published before the mega-trials and aligned with the mega-trials. Most mega-trials do not show statistically significant benefits for the primary outcome of interest, and statistically significant benefits for mortality are rare. Mega-trials are not available for most medical studies. Given that small trials and their meta-analyses may give unreliable, inflated estimates of benefit, mega-trials, or at least substantially large trials with sufficient power, may need to be considered and performed more frequently.

The diminished benefits in late smaller trials vs early small trials were also consistent with prior meta-research studies^146^ that have shown that the reported effects of interventions change over time, with wider oscillations of results in early studies. It has been observed that it is more frequent for treatment effects to decrease rather than increase over time.^147,148,149^ In our examined studies, the mega-trials may have corrected some inflated effects seen in the earlier trials that preceded them. Then, the subsequent trials might have been more aligned with what the mega-trials had shown because the mega-trials are likely to have been considered very influential.

Previous meta-research assessments have shown different levels of agreement between the results of meta-analyses of smaller trials and large clinical trials. For example, Cappelleri et al^11^ reported compatible results of meta-analysis of smaller studies with the results of large trials, although discrepancies in their results were found in up to 10% of the cases. However, other meta-studies on this topic^13^ showed larger differences with a discrepancy rate of up to 39%. These previous studies used a definition of a large trial having enrolled 1000 participants or more. In contrast, we used a sample size of 10 000 participants to define a mega-trial, and therefore had a larger power to detect effects.

### Limitations

This study has limitations. Several early mega-trials are not included in the ClinicalTrials.gov registry. Nevertheless, we were able to identify several of these trials because they were included in the meta-analyses of other mega-trials, and they were considered in our calculations.

Our comparative results vs smaller trials still did not include all mega-trials, because for some mega-trials retrieved in ClinicalTrials.gov, we found no relevant meta-analysis where they had been included. However, we did examine the main conclusions of these mega-trials and they also had low rates of statistically significant results. Therefore, we can conclude that mega-trials in general tend to give negative results for tested interventions.

Mega-trials may have, on average, more pragmatic designs than smaller trials. The different eligibility criteria and different populations of participants enrolled in mega-trials vs smaller trials may create differences in effect sizes. Addressing such differences in case-mix heterogeneity would require individual-level data.

Mega-trials are unlikely to be launched unless there is genuine equipoise. Nevertheless, the low rate of significant benefits, as opposed to the much higher rates of favorable results seen in typical phase 3 trials, is remarkable.^150^ Previous research found more favorable results in industry-funded research.^150,151^ Finally, our analyses depend on the accuracy and quality of data extracted from the included meta-analyses.

## Conclusions

In this meta-research analysis, meta-analyses of smaller studies showed, in general, comparable results with mega-trials, but smaller trials published before the mega-trials gave more favorable results than the mega-trials. Mega-trials are done very sparingly to date, but it would be beneficial to add more of these trials to the clinical research armamentarium.^152,153^

## Notes

Supplement 1. **eAppendix 1.** Search Strategy **eAppendix 2.** Data Extraction **eTable 1.** The Composite Primary Outcome and Effect Estimates of Mega-Trials Identified by Our Search but Analyzed Only for a Subset of the Primary Outcome **eAppendix 3.** Mega-Trials Not Included in Meta-Analyses **eTable 4.** Characteristics of Mega-Trials Identified by Our Search but Had No Eligible Meta-Analysis **eTable 2.** Characteristics of the Additional Identified Mega-Trials That Have Not Been Identified by Our Search **eAppendix 4.** Meta-Analyses of Mega-Trials vs Smaller Trials for the Primary Outcome **eFigure 1.** Agreement Between Mega-Trials and Smaller Trials for Primary Outcome: Random Effects (DerSimonian Laird) **eAppendix 5.** Meta-Analyses of Mega-Trials vs Smaller Trials for All-Cause Mortality **eFigure 2.** Agreement Between Mega-Trials and Smaller Trials for All-Cause Mortality: Random Effects (DerSimonian Laird) **eFigure 3.** Agreement Between Smaller Trials Prior and After the Publication of the First Mega-Trial **eTable 3.** Results of Uni- and Multivariable Meta-Regression **eFigure 4.** Agreement Between Mega-Trials and Smaller Trials With 1/5 of the Least Weighted Mega-Trial **eFigure 5.** Agreement Between Mega-Trials and Smaller Trials With 1/10 of the Least Weighted Megatrial **eFigure 6.** Agreement Between Mega-Trials and Smaller Trials, Pooling the Results Using Fixed Effects **eFigure 7.** Agreement Between Mega-Trials and Smaller Trials, Pooling the Results Using Random Effects – HKSJ Method **eFigure 8.** Agreement Between Mega-Trials and Smaller Trials Stratified to Blinding **eFigure 9.** Agreement Between Mega-Trials and Smaller Trials Stratified to Intervention Type **eFigure 10.** Agreement Between Mega-Trials and Smaller Trials Stratified to Specialty **eFigure 11.** Agreement Between Mega-Trials and Smaller Trials Stratified to Heterogeneity **eFigure 12.** Agreement Between Trials With More Than 30,000 Participants and Smaller Trial for the Primary Outcome **eFigure 13.** Agreement Between Mega-Trials When More Than One Was Present in a Meta-Analysis–Primary Outcome
eReferences.

Supplement 2.
Data Sharing Statement
