# Supplementary material for: Agreement Between Mega-Trials and Smaller Trials: A Systematic Review and Meta-Research Analysis
Source: JAMA Netw Open. 2024 Sep 6;7(9):e2432296. doi: 10.1001/jamanetworkopen.2024.32296 (PMC11380108; doi:10.1001/jamanetworkopen.2024.32296)
Supplement: Supplement 1. — eAppendix 1. Search Strategy eAppendix 2. Data Extraction eTable 1. The Composite Primary Outcome and Effect Estimates of Mega-Trials Identified by Our Search but Analyzed Only for a Subset of the Primary Outcome eAppendix 3. Mega-Trials Not Included in Meta-Analyses eTable 4. Characteristics of Mega-Trials Identified by Our Search but Had No Eligible Meta-Analysis eTable 2. Characteristics of the Additional Identified Mega-Trials That Have Not Been Identified by Our Search eAppendix 4. Meta-Analyses of Mega-Trials vs Smaller Trials for the Primary Outcome eFigure 1. Agreement Between Mega-Trials and Smaller Trials for Primary Outcome: Random Effects (DerSimonian Laird) eAppendix 5. Meta-Analyses of Mega-Trials vs Smaller Trials for All-Cause Mortality eFigure 2. Agreement Between Mega-Trials and Smaller Trials for All-Cause Mortality: Random Effects (DerSimonian Laird) eFigure 3. Agreement Between Smaller Trials Prior and After the Publication of the First Mega-Trial eTable 3. Results of Uni- and Multivariable Meta-Regression eFigure 4. Agreement Between Mega-Trials and Smaller Trials With 1/5 of the Least Weighted Mega-Trial eFigure 5. Agreement Between Mega-Trials and Smaller Trials With 1/10 of the Least Weighted Megatrial eFigure 6. Agreement Between Mega-Trials and Smaller Trials, Pooling the Results Using Fixed Effects eFigure 7. Agreement Between Mega-Trials and Smaller Trials, Pooling the Results Using Random Effects – HKSJ Method eFigure 8. Agreement Between Mega-Trials and Smaller Trials Stratified to Blinding eFigure 9. Agreement Between Mega-Trials and Smaller Trials Stratified to Intervention Type eFigure 10. Agreement Between Mega-Trials and Smaller Trials Stratified to Specialty eFigure 11. Agreement Between Mega-Trials and Smaller Trials Stratified to Heterogeneity eFigure 12. Agreement Between Trials With More Than 30,000 Participants and Smaller Trial for the Primary Outcome eFigure 13. Agreement Between Mega-Trials When More Than One Was Present [file jamanetwopen-e2432296-s001.pdf]

Supplemental Online Content

Kastrati L, Raeisi-Dehkordi H, Llanaj E, et al. Agreement between mega-trials and smaller trials: a meta-research study. *JAMA Netw Open*. 2024;7(9):e2432296. doi:10.1001/jamanetworkopen.2024.32296

eAppendix 1. Search Strategy
eAppendix 2. Data Extraction
eTable 1. The Composite Primary Outcome and Effect Estimates of Mega-Trials Identified by Our Search but Analyzed Only for a Subset of the Primary Outcome
eAppendix 3. Mega-Trials Not Included in Meta-Analyses
eTable 4. Characteristics of Mega-Trials Identified by Our Search but Had No Eligible Meta-Analysis
eTable 2. Characteristics of the Additional Identified Mega-Trials That Have Not Been Identified by Our Search
eAppendix 4. Meta-Analyses of Mega-Trials vs Smaller Trials for the Primary Outcome
eFigure 1. Agreement Between Mega-Trials and Smaller Trials for Primary Outcome: Random Effects (DerSimonian Laird)
eAppendix 5. Meta-Analyses of Mega-Trials vs Smaller Trials for All-Cause Mortality
eFigure 2. Agreement Between Mega-Trials and Smaller Trials for All-Cause Mortality: Random Effects (DerSimonian Laird)
eFigure 3. Agreement Between Smaller Trials Prior and After the Publication of the First Mega-Trial
eTable 3. Results of Uni- and Multivariable Meta-Regression
eFigure 4. Agreement Between Mega-Trials and Smaller Trials With 1/5 of the Least Weighted Mega-Trial
eFigure 5. Agreement Between Mega-Trials and Smaller Trials With 1/10 of the Least Weighted Megatrial
eFigure 6. Agreement Between Mega-Trials and Smaller Trials, Pooling the Results Using Fixed Effects
eFigure 7. Agreement Between Mega-Trials and Smaller Trials, Pooling the Results Using Random Effects–HKSJ Method
eFigure 8. Agreement Between Mega-Trials and Smaller Trials Stratified to Blinding
eFigure 9. Agreement Between Mega-Trials and Smaller Trials Stratified to Intervention Type
eFigure 10. Agreement Between Mega-Trials and Smaller Trials Stratified to Specialty
eFigure 11. Agreement Between Mega-Trials and Smaller Trials Stratified to Heterogeneity
eFigure 12. Agreement Between Trials With More Than 30,000 Participants and Smaller Trial for the Primary Outcome
eFigure 13. Agreement Between Mega-Trials When More Than One Was Present in a Meta-Analysis–Primary Outcome
eReferences.

This supplemental material has been provided by the authors to give readers additional information about their work.

© 2024 Kastrati L et al. *JAMA Network Open.*

eAppendix 1. Search Strategy

Using the filters for phase 3 and 4 trials and sample size we screened clinical trials registered for clinicaltrials.gov. We iterated the process for each listed country separately. After identifying the eligible clinical trials, we searched the first primary publication for these trials that included any primary outcome(s) registered in clinicaltrials.gov. If no primary publication was registered in clinicaltrials.gov, we searched PubMed.

Next, in PubMed, we used the option "cited by" and selected further the option "meta-analysis" to identify meta-analyses that had cited the papers of interest. If more than one meta-analysis was identified, we screened them starting with the most recently indexed one and moving backward until a suitable meta-analysis was found that included the mega-trial results in calculations of a summary effect for a primary endpoint of the mega-trial. If summary effect calculations for multiple primary endpoints of the mega-trial were presented in an eligible meta-analysis, we prioritized binary over continuous outcomes; and the primary outcome with the largest number of events in the mega-trial.

When information on all-cause mortality was not available in the eligible meta-analysis for primary outcome, we similarly screened meta-analyses that had cited the earliest main publication of the mega-trials backward in time until the 10th meta-analysis. The same process in PubMed was used to identify citing meta-analyses.

Whenever a mega-trial included more than one active arm versus control, e.g. in a three-arm trial, or two or more different comparisons, e.g. in factorial design, we considered each eligible comparison separately and tried to identify respective meta-analyses.

Clinicaltrials.gov searches were last updated on 10/01/2023. PubMed searches were last updated on June 2023 by independent screeners and were done in duplicate.

© 2024 Kastrati L et al. *JAMA Network Open.*

eAppendix 2. Data Extraction

For each selected meta-analysis, we extracted the results of RCTs included in the summary effect estimate that incorporated the effect estimate of the mega-trial. For each selected meta-analysis, we recorded the first author's name, publication year, eligible endpoint, comparison of intervention versus control, type of masking, topic, and type of intervention. For each trial in each eligible meta-analysis, we recorded the first author's name or acronym, publication year, total sample size, 2x2 table (or log (odds ratio) and variance thereof, if 2x2 table was not provided) for dichotomized outcomes and ~~standardized mean difference (and variance thereof) for continuous outcomes~~. We also extracted information, whenever available, on the risk of bias assessments for each included trial based on Cochrane risk of bias tools (original, revised, and version 2).

All data extractions were performed in duplicate (except mega-trial identification), and differences were settled by discussion. For any unsettled discrepancies, a third senior reviewer was invited to arbitrate.

Mega-trials (and their corresponding meta-analyses) that compare two active and overlapping interventions were analyzed as follows: if one intervention is a subset of the other, the subset intervention was considered as the control arm (e.g. in a trial comparing X+Y+Z to X+Y, X+Y was the control arm). If the interventions were not subsets of each other (e.g., X versus Y), the intervention that was approved by the FDA first was considered as the control arm.

© 2024 Kastrati L et al. *JAMA Network Open.*

eTable 1. The Composite Primary Outcome and Effect Estimates of Mega-Trials Identified by Our Search but Analyzed Only for a Subset of the Primary Outcome

MEGA-TRIAL	Primary outcome	Intervention	Control	Primary outcome OR (95% CI)
**ACCOMPLISH 2008 [1]**	MACE	ACEi/ARBs+CCB	Other combinations	0.8 (0.72-0.9)
**GLOBAL LEADER 2018 [2]**	All-cause mortality or new Q-wave myocardial infarction	Very short duration of antiplatelet therapy	>3 months antiplatelet therapy	0.87 (0.75–1.01)
**NISSEN 2016 [3]**	Cardiovascular death	Celecoxib	Naproxen	0.9 (0.71-1.15)
		Celecoxib	Ibuprofen	0.81 (0.65-1.02)
**EXTRACT TIMI 2006 [4]**	Death or nonfatal recurrent myocardial infarction	Enoxaparin	Heparin	0. 9 (0.8-1.01)
**BEAUTIFUL 2008 [5]**	MACE	Ivabradine	Placebo	1.00 (0.91-1.10)
**HPS-2 THRIVE 2004 [6]**	MACE	Niacin-laropiprant	Placebo	0.96 (0.9-1.03)
**ILLUMINATE 2007 [7]**	MACE	Torcetrapib+Atorvastatatin	Atorvastatin	1.25 (1.09-1.44)
**PLATO 2009 [8]**	MACE	Ticagrelor	Clopidogrel	0.85 (0.77-0.93)
**CURRENT OASIS 7 2010[9]**	MACE	Double dose clopidogrel	Standard dose	0.85 (0.73-0.98)
**POISE-2 2014 [10]**	MACE	Clonidine	Placebo	1.08 (0.93-1.26)
**ENGAGE TIMI AF [11]**	MACE	High dose Edoxaban	Warfarin	0.89 (0.83-0.96)
		Low dose Edoxaban		0.83 (0.77-0.9)
**SOLID TIMI 52 2014 [12]**	MACE	Darapladib	Placebo	1.00 (0.91-1.09)
**ONTARGET 2008[13]**	MACE	Ramipril	Telmisartan	1.01 (0.94-1.09)
**REVEAL 2017 [14]**	MACE	Anacetrapib	Placebo	0.91 (0.85-0.97)
**ARRIVE 2018 [15]**	MACE	Aspirin	Placebo	0.96 (0.81-1.13)
**ACCELERATE 2017 [16]**	MACE	Evacetrapib	Placebo	1.01 (0.90-1.10)
**Dal-OUTCOME 2012 [17]**	MACE	Dalcetrapib	Placebo	1.04 (0.92-1.16)
**TRITON TIMI 2007 [18]**	MACE	Prasugrel	Clopidogrel	0.81 (0.73-0.9)
**CHAMPION 2016 [19]**	MACE	Cangrelor	Clopidogrel	0.83 (0.67-1.01)

MACE- Major Adverse Cardiovascular Events,

© 2024 Kastrati L et al. *JAMA Network Open.*

eAppendix 3. Mega-Trials Not Included in Meta-Analyses

Of the 38 mega-trials that were otherwise eligible but for which we could not retrieve any meta-analysis that included them, 9/38 had a statistically significant benefit at $p<0.05$ for the primary outcome (all favoring the intervention) and 5/38 had significant results for all-cause mortality (all favoring intervention).

eTable 4. Characteristics of Mega-Trials Identified by Our Search but Had No Eligible Meta-Analysis

MEGA-TRIAL	Primary outcome	Intervention	Control	Primary Outcome	All-Cause Mortality	NonInferiority
**COSMOS 2022 [51]**	MACE	Cocoa Extract	Placebo	Non-Significiant	Non-Significant	-
**NAITRE 2022 [52]**	All-cause Mortaility	Azithromycin	Placebo	Non-Significiant	Non-Significiant	-
**ARISTOTLE 2011 [53]**	Stroke or systemic embolism	Apixaban	Warfarin	Significant	Significant	Noninferiority
**THEMIS-PCI 2019 [54]**	MACE	Ticargrelor	Placebo	Significiant	Non-Significiant	-
**RE-LY 2010 [55]**	MACE	Dabigatran	Warfarin	Non-significant	Non-Significant	Noninferiority
**SOCRATES 2016 [56]**	MACE	Ticargrelor	Placebo	Non-significant	Non-significant	-
**TAO 2013 [57]**	All-cause mortality or Myocardial Infarction	Otamixaban	Unfractioned Heparin + Eptifibatide	Non-significant	Non-significant	-Superiority
**THALES 2020 [58]**	Stroke or Death	Ticargrelor-Aspirin	Aspirin	Significant	Non-significant	Superiority
**COPD 2018 [59]**	Rate of severe COPD	Triple Therapy	Duo-Therpy	Significant	Significant	-
**TIOSPIR 2013 [60]**	Death and COPD	Tiotropium Respimat	Tiotropium HandiHaler	Non-significant	Non-significant	Noninferiority
**NISSEN 2023 [61]**	MACE	Bempedoic Acid	Placebo	Significant	Significant	-
**AUSTRI 2016 [62]**	Serious asthma-related event	Fluticasone-Salmeterol	Flucitasone Alone	Non-Significant	Non-Significant	Noninferiority
**SUMMIT 2016 [63]**	All-cause mortality	Fluticasone furate Vilaterol Combination	Placebo	Non-Significant	Non-Significant	-
**MARINER 2018 [64]**	Composite of any symptomatic VTE	Rivaroxaban	Placebo	Non-Significant	Non-Significant	Superiority
**STAR 2006 [65]**	Invasive breast cancer	Tamoxifen	Raloxifene	Non-Significant	Non-Significant	-
**EUCLID 2017 [66]**	MACE	Ticargrelor	Clopidogrel	Non-Significant	Non-Significant	Superiority
**Peters 2016 [67]**	Serious asthma-related event	Budesonide + Formoterol	Budesonide	Non-Significant	Non-Significant	Non-inferiority
**SELECT 2009 [68]**	Prostate cancer	Selenium /+ Vitamin E	Placebo	Non-Significant	Non-Significant	-
**RUTH 2006 [69]**	Coronary events and invasive breast cancer	Raloxifene	Placebo	Non-Significant	Non-Significant	-
**Christen 2015 [70]**	Incident Cataract	Selenium	Vitamin E	Non-Significant	NA	-
**SCOUT 2010 [71]**	MACE	Sibutramine	Placebo	Non-Significant	Non-Significant	-
**WOMAN 2017 [72]**	All-cause mortality or hysterectomy	Tranexamic acid	Placebo	Non-Significant	Non-Significant	-
**Chandramohan 2019 [73]**	Death or admission not due to trauma	Malaria chemoprevention + Azithromycin	Malaria chemoprevention + Placebo	Non-Significant	Non-Significant	-
**CRASH-3 2019 [74]**	Head injury related death	Tranexamic acid	Placebo	Non-Significant	Non-Significant	-
**CRASH-2 2010 [75]**	Death in hospital within 4 weeks of injury	Tranexamic acid	Placebo	Significant	Significant	-
**HEAT 2015 [76]**	Time to hospitalization or death due to peptic ulcer bleeding	Clarithromycin, metronidazole and lansoprazole	Placebo	Non-Significant	Non-Significant	-

© 2024 Kastrati L et al. *JAMA Network Open.*

**HALT-IT 2020 [77]**	Death due to bleeding	Tranexamic Acid	Placebo	Non-Significant	Non-Significant	-
**BRUNVOLL 2022 [78]**	4 coprimary outcomes related to respiratory infections	Cod liver oil	Placebo	Non-Significant	NA	-
**CRESCENDO 2010 [79]**	MACE	Rimonabant	Placebo	Non-Significant	Non-Significant	-
**ROC 2011[80]**	Survival to discharge	Early analysis of cardiac rhythm	Late analysis of cardiac rhythm	Non-Significant	NA	-
**PROMINENT 2022 [81]**	MACE	Pemafibrate	Placebo	Non-Significant	Non-Significant	-
**Sazawal 2016 [82]**	Mortality	Chlorhexidine	Dry cord care	Non-Significant	Non-Significant	-
**Ishani 2022 [83]**	MACE	Chlorthalidone	Hydrochlorthiazide	Non-Significant	Non-Significant	Superiority
**ZODIAC 2011 [84]**	Non-suicide mortality	Ziprasidone	Olanzapine	Non-Significant	Non-Significant	-
**NORDIC 2016 [85]**	Participation in colonoscopy screening, cancer and adenoma yield, and participant experience	Colonoscopy	No Screening	Not Clear	NA	-
**Hygia 2020 [86]**	MACE	Antihypertensive drugs in the morning	Antihypertensive drugs in the night	Significant	Significant	-
**Hansen 2012 [87]**	Difference in physical activity	Web-based physical activity promotion	No intervention	Significant	NA	-
**ROCKET AF 2011 [88]**	Stroke or Systemic Embolism	Rivaroxamban	Warfarin	Significant	Non-Significant	Noninferiority

MACE- Major Adverse Cardiovascular Events
COPD- Chronic Obstructive Pulmonary Disease

© 2024 Kastrati L et al. *JAMA Network Open.*

eTable 2. Characteristics of the Additional Identified Mega-Trials That Have Not Been Identified by Our Search

Mega-Trial	Primary Outcome	Intervention	Control	Primary Outcome OR (95% CI)	All-Cause Mortality OR (95% CI)	Risk of Bias
**VALUE 2004 [20]**	MACE	Valsartan	Amlodipin	1.04 (0.94-1.15)	1.04 (0.94-1.14)	Low
**ATBC 2003 [21]**	Site-specific cancer incidence and total and cause-specific mortality and calendar time-specific risk for lung cancer incidence and total mortality.	Beta carotene	Placebo	Only one of sixteen outcomes had significant findings	Not significant Multiple outcome estimates	Low
**Omenn 1996. [22]**	Incidence of lung cancer	Beta carotene and Vitamin A	Placebo	1.28 (1.05-1.57)	1.17 (1.03-1.33)	Low
**Hennekens 1996 [23]**	Malignant neoplasms and cardiovascular disease	Beta carotene	Placebo	0.98 (0.91-1.06)	1.01 (0.92-1.11)	Low
**Goodman 2004 [24]**	Lung cancer	Beta carotene	Placebo	1.12 (0.97-1.31)	1.08 (0.99-1.17)	Low
**ISIS-1 1986 [25]***	Vascular Mortality	Atenolol	Control	0.84 (0.72-0.99)	0.85 (0.73-0.99)	High
**REAL CAD 2018 [26] ***	MACE	High dose Pitavastatin	Low dose Pitavastatin	0.81 (0.69-0.95)	0.81 (0.68-0.98)	High
**SEARCH 2010 [27]**	MACE	80mg simvastatin	20mg simvastatin	0.94 (0.88-1.01)	0.99 (0.91-1.09)	Low
**TNT 2005 [28]**	MACE	80mg simvastatin	10mg simvastatin	0.78 (0.69-0.89)	1.01 (0.85-1.19)	Low
**ASCOT LLA 2003 [29]***	Non-fatal myocardial infarction and fatal CHD	Atorvastatin	Placebo	0.64 (0.5-0.83)	0.87 (0.71-1.06)	Low
**ALLHAT 2002 [30]***	Fatal CHD or nonfatal myocardial infarction	Amlodipine<br>Lisinopril	Chlorthalidone	0.98 (0.9-1.07)<br>0.99 (0.91-1.08)	0.96 (0.89-1.02)<br>1.00 (0.94-1.08)	High
**Lacroix 2009 [31]**	Mortality	Calcium + Vitamine D	Placebo	0.91 (0.83-1.01)	0.91 (0.83-1.01)	Low
**HOT 1998 [32]**	MACE	Aspirin	Placebo	0.85 (0.73-0.99)	0.93 (0.79-1.09)	Low
**DeKoning 2020 [33]***	Lung Cancer Mortality	Screening	Control	0.74 (0.-61-0.90)	0.98 (0.89-1.08)	High
**GISSI-P 1999 [34]***	MACE	n-3 PUFA<br>vitamin E<br>both	Placebo	0.90 (0.82–0.99)<br>0.95 (0.86–1.05)<br>0.86 (0.74–0.99)	0.86 (0.76–0.97)<br>0.92 (0.82–1.04)	Low
**Leppälä 2000 [35]**	Stroke mortality	α-Tocopherol<br>β-Carotene<br>both	Placebo	Non-significant	NA	Low
**Lee 1999 [36]**	Cancer and cardiovascular mortality	β-Carotene	Placebo	1.03 (0.89-1.18)<br>1.14 (0.87-1.49)	1.07 (0.74-1.56)	High
**ASPREE 2018 [37]**	Death, dementia, or persistent physical disability	Aspirin	Placebo	1.01 (0.98-1.11)	1.14 (1.01-1.29)	Low

© 2024 Kastrati L et al. *JAMA Network Open.*

**WHS 2005 [38]**	MACE	Aspirin	Placebo	0.91 (0.8-1.03)	0.95 (0.85-1.06)	Low
**CAPPP 1999 [39]***	MACE	Captopril	Conventional antihypertensives	1.05 (0.9-1.22)	0.93 (0.76-1.14)	Low
**ASCOT-BPLA 2005 [40]***	MACE	Amlodipine	Atenolol	0.9 (0.79-1.02)	0.89 (0.81-0.99)	Low
**CONVINCE 2003 [41]**	MACE	Verapamil	Atenolol or Hydrochlorthiazide	1.02 (0.88-1.18)	1.08 (0.93-1.26)	Low
**NORDIL 2000 [42]***	MACE	Diltiazem	Diuretic and Beta-blockers	1.00 (0.87-1.15)	1.00 (0.83-1.20)	Low
**ADVANCE 2008 [43]***	MACE and Major microvascular events	Intensive glucose control	Standard control	0.88 (0.8-0.97)	0.92 (0.81-1.05)	High
**WHI 2006 [44]**	Colorectal Cancer	Calcium	Placebo	1.08 (0.86-1.34)	0.91 (0.83-1.01)	Low
**IMPROVE IT 2015 [45]**	MACE	Ezetimibe + Statin	Statin	0.94 (0.89-0.99)	0.99 (0.91-1.07)	Low
**FOURIER 2017 [46]**	MACE	Evolocumab	Placebo	0.85 (0.79-0.92)	1.04 (0.91-1.19)	Low
**TECOS 2015 [47]^**	MACE	Sitagliptin	Placebo	0.98 (0.88-1.09)	1.01 (0.9-1.14)	Low
**STRENGTH 2020 [48]**	MACE	Omega-3	Corn oil	0.99 (0.9-1.09)	1.13 (0.97-1.42)	Low
**MRC/BHF 2002[49]**	MACE	Vitamins	Placebo	0.99 (0.93-1.06)	1.03 (0.92-1.17)	Low
**MRC/BHF 2002[50]**	All-cause Mortality	Simvastatin	Placebo	0.87 (0.79-0.93)	0.87 (0.79-0.93)	Low

OR– Odds Ratio, **CI**- Confidence Intervals, **ACEi-** Angiotensin Converting Enzyme inhibitors ,**ARBs-** Angiotensin Receptor Blockers
MACE- Major Adverse Cardiovascular Events, **PUFA-** Polyunsaturated Fatty Acids
Trials denoted with * had an open-label design
Trials denoted with ^ were designed to show non-inferiority

© 2024 Kastrati L et al. *JAMA Network Open.*

eAppendix 4. Meta-Analyses of Mega-Trials vs Smaller Trials for the Primary Outcome

1. Aberle 2011

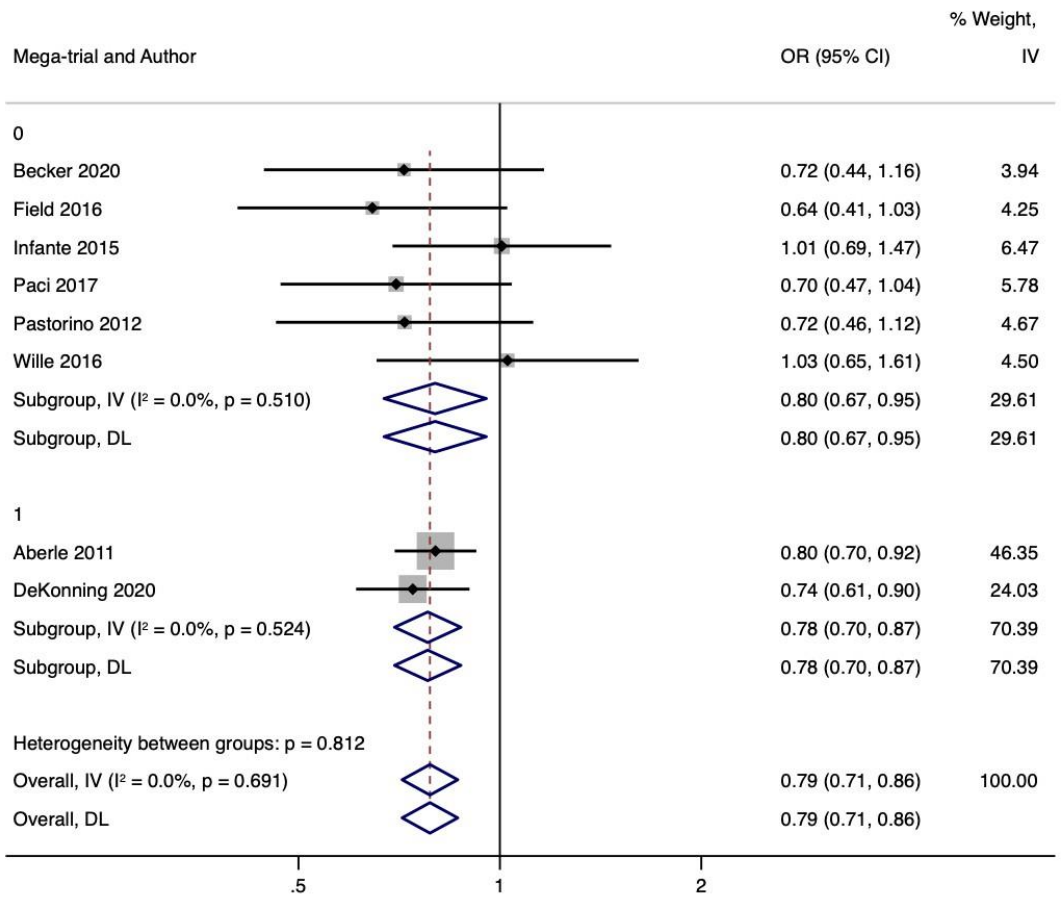

© 2024 Kastrati L et al. *JAMA Network Open.*

2. ACCOMPLISH 2008

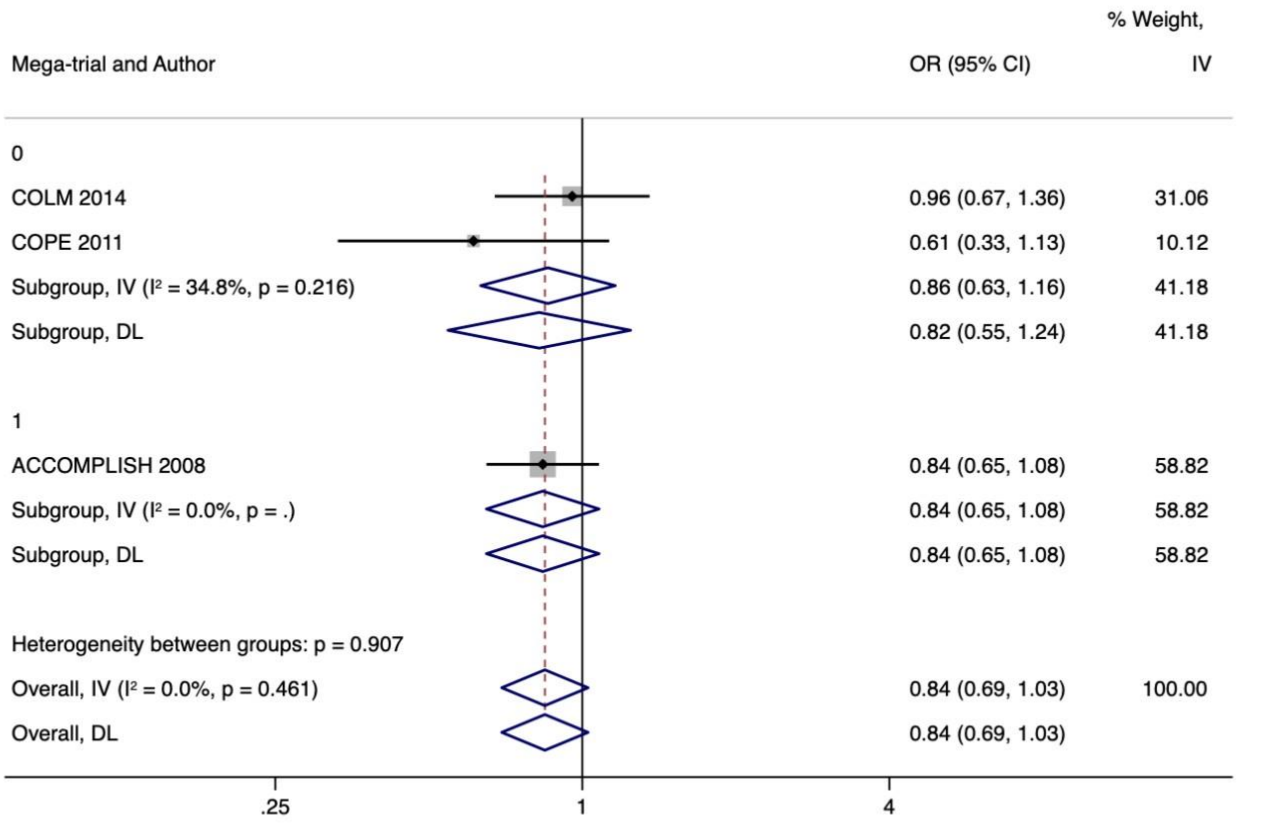

© 2024 Kastrati L et al. *JAMA Network Open.*

3. CSPTT 2015

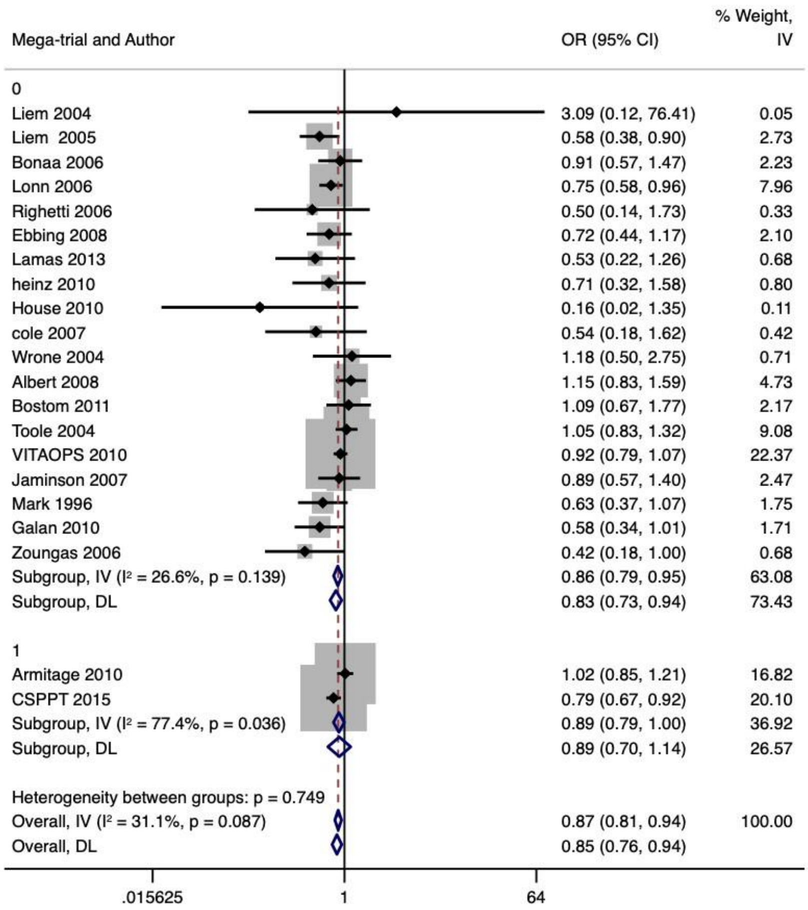

© 2024 Kastrati L et al. *JAMA Network Open.*

4. ORIGIN 2012

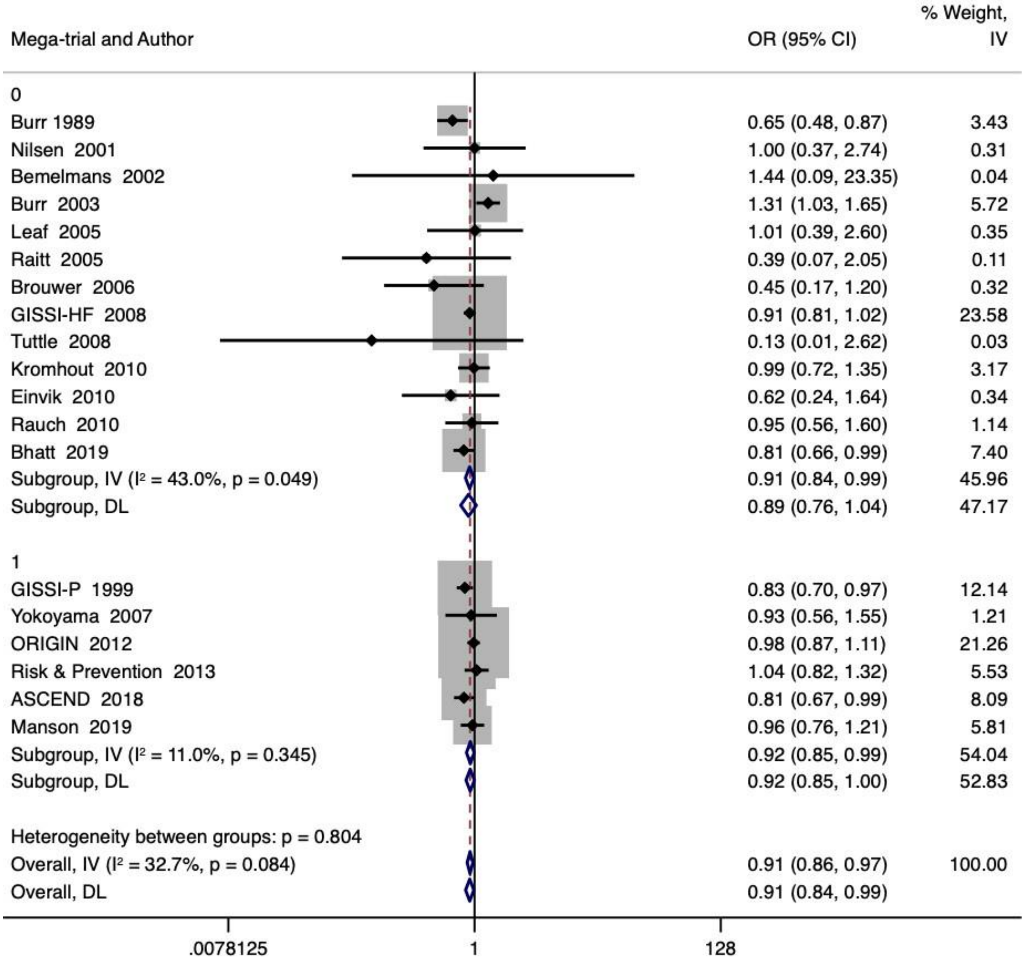

© 2024 Kastrati L et al. *JAMA Network Open.*

5. GLOBAL LEADERS 2018

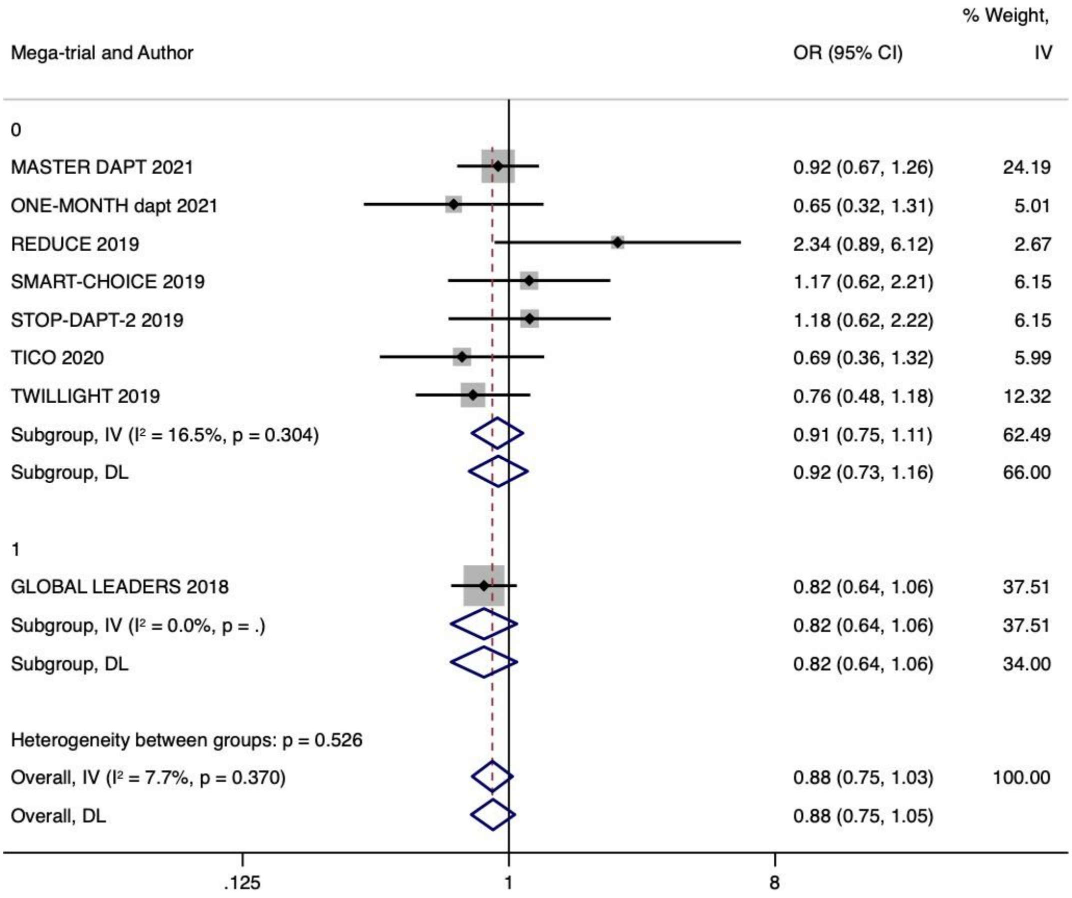

© 2024 Kastrati L et al. *JAMA Network Open.*

6. ACCORD 2008

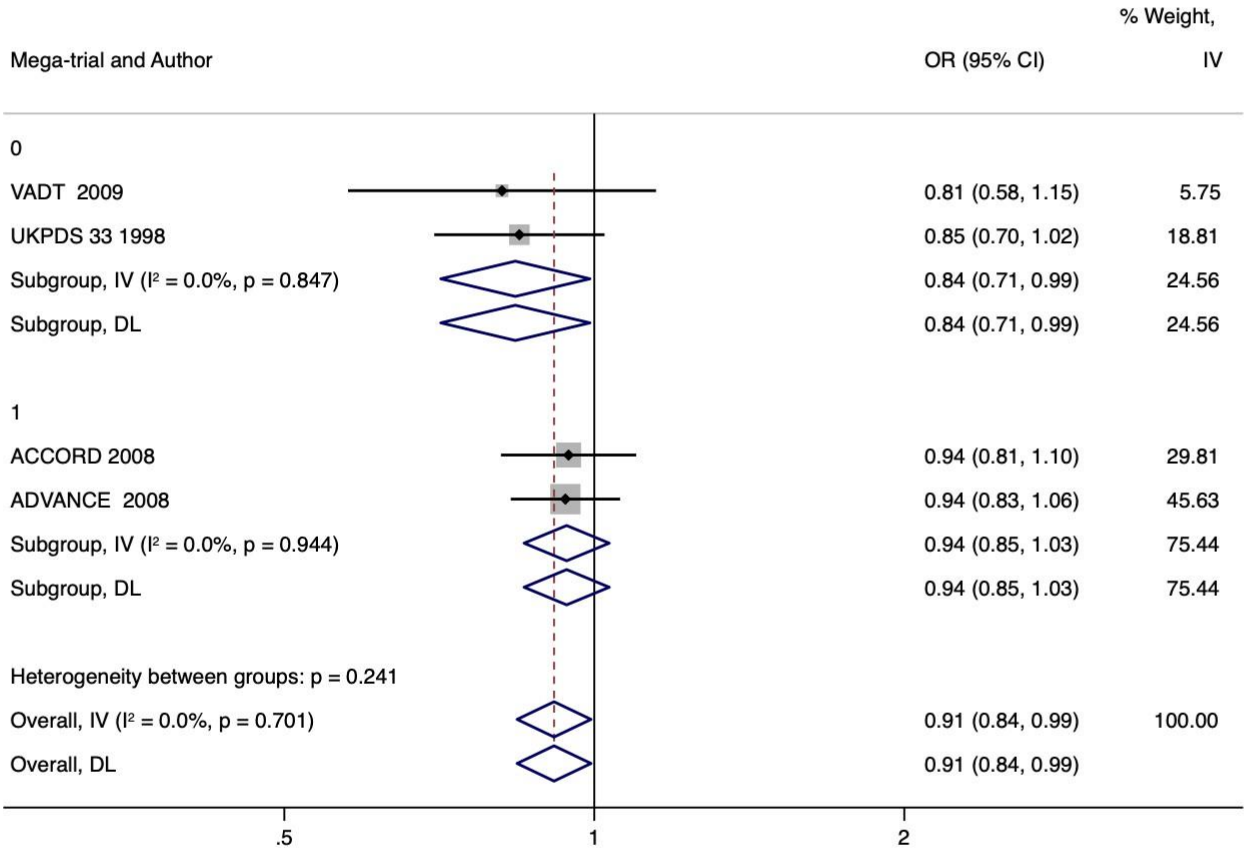

© 2024 Kastrati L et al. *JAMA Network Open.*

7. INVEST 2003

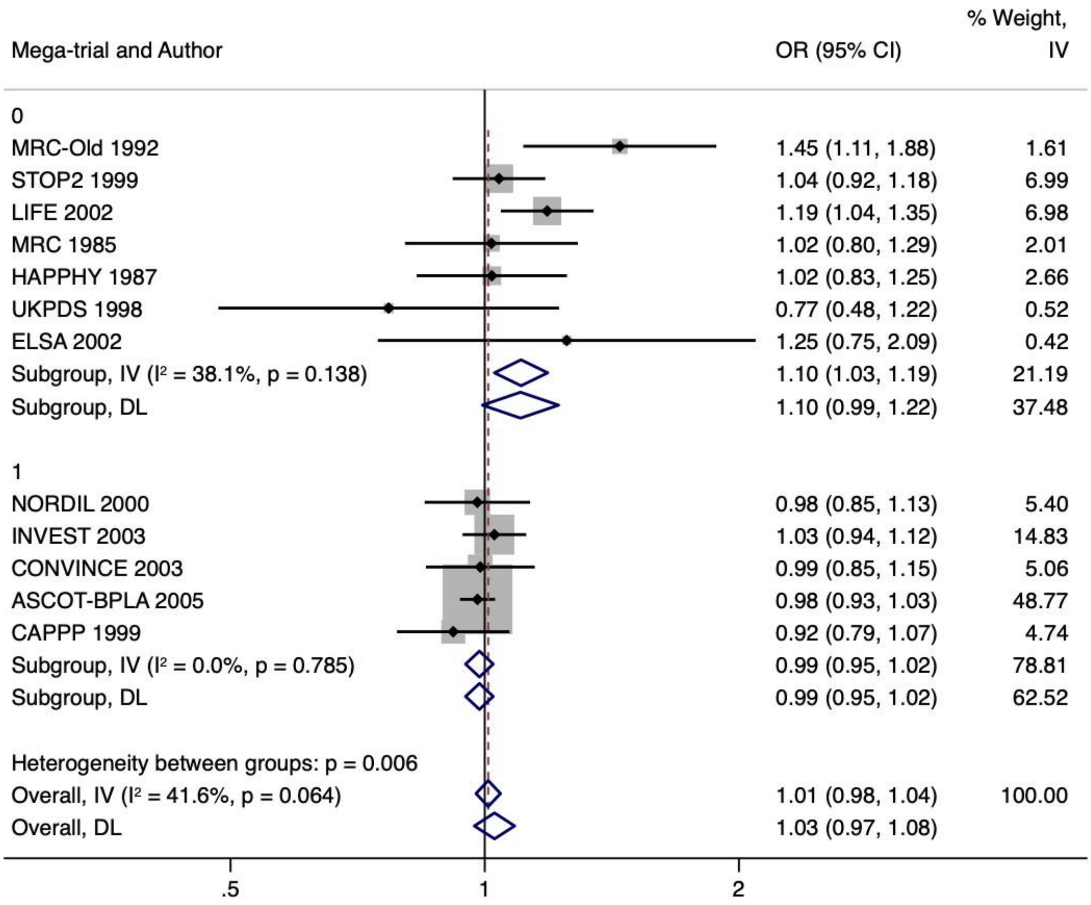

© 2024 Kastrati L et al. *JAMA Network Open.*

8. JELIS 2007

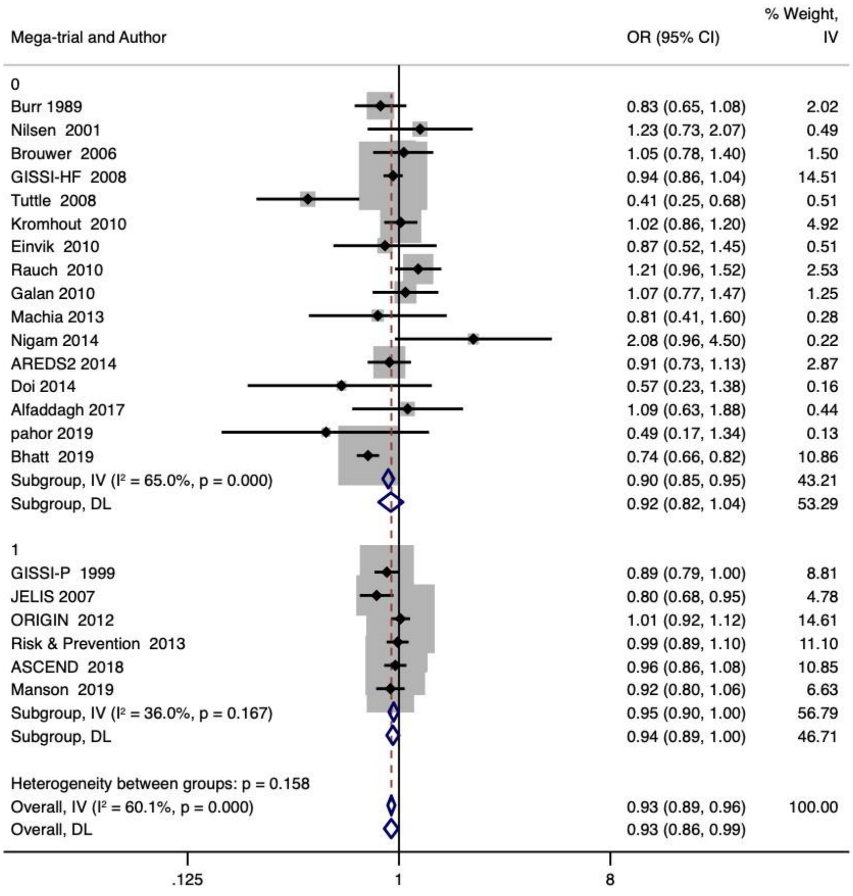

© 2024 Kastrati L et al. *JAMA Network Open.*

9. JPPP 2014

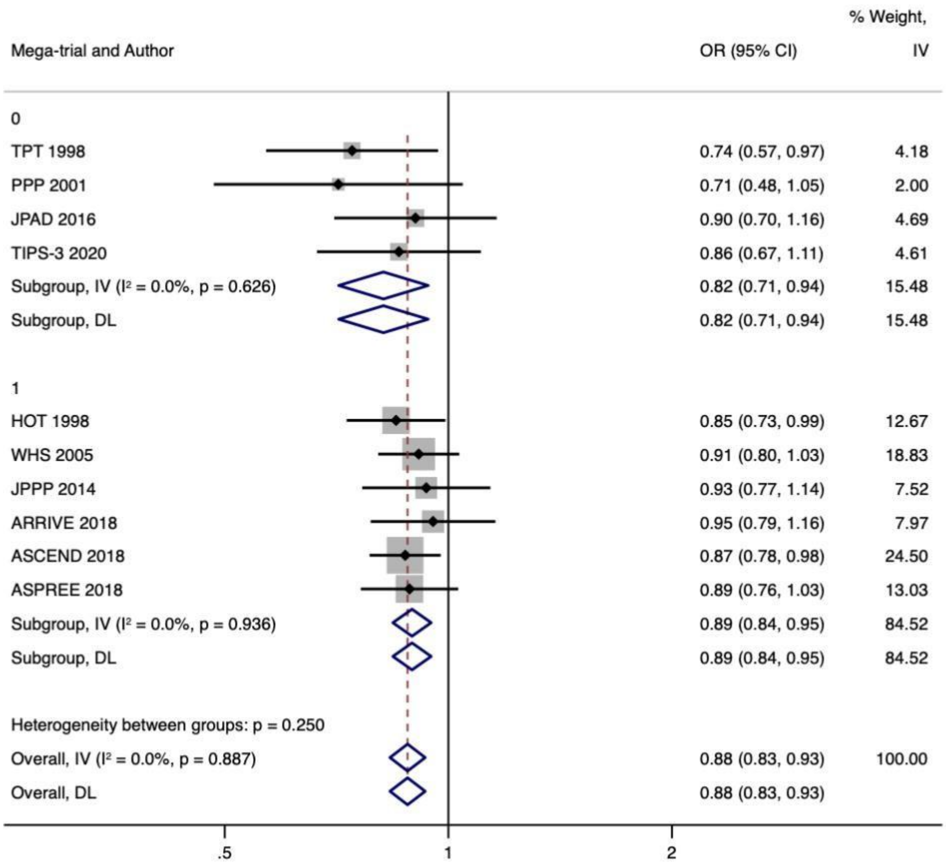

© 2024 Kastrati L et al. *JAMA Network Open.*

10. VITAL 2019

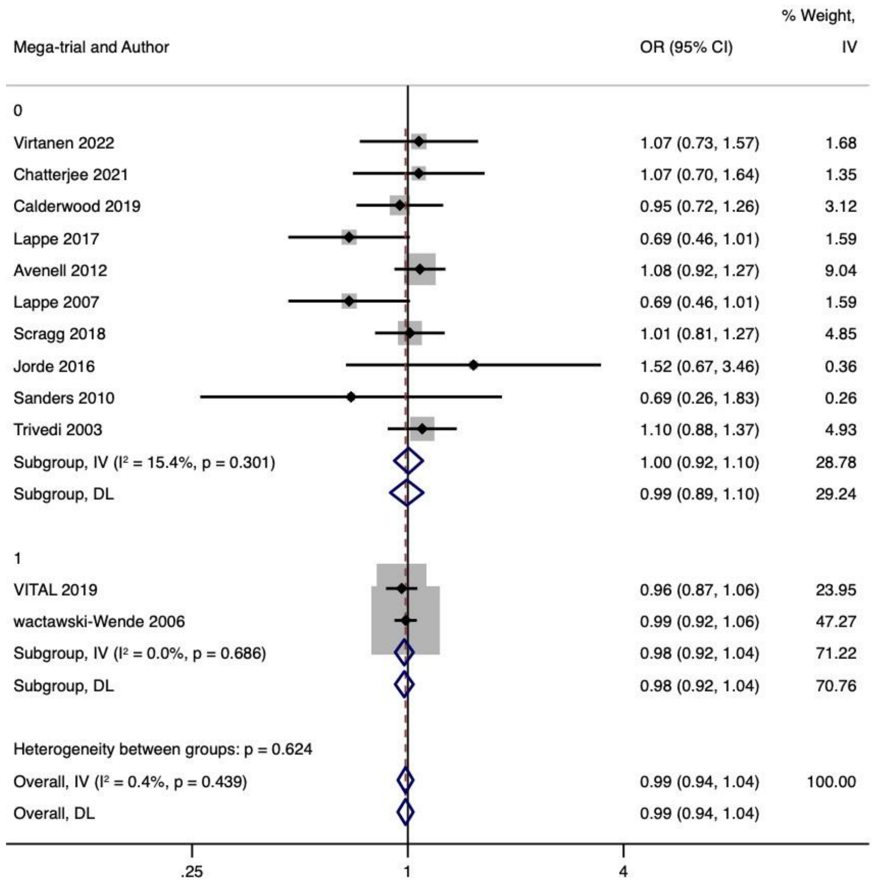

© 2024 Kastrati L et al. *JAMA Network Open.*

11. Albert 2021

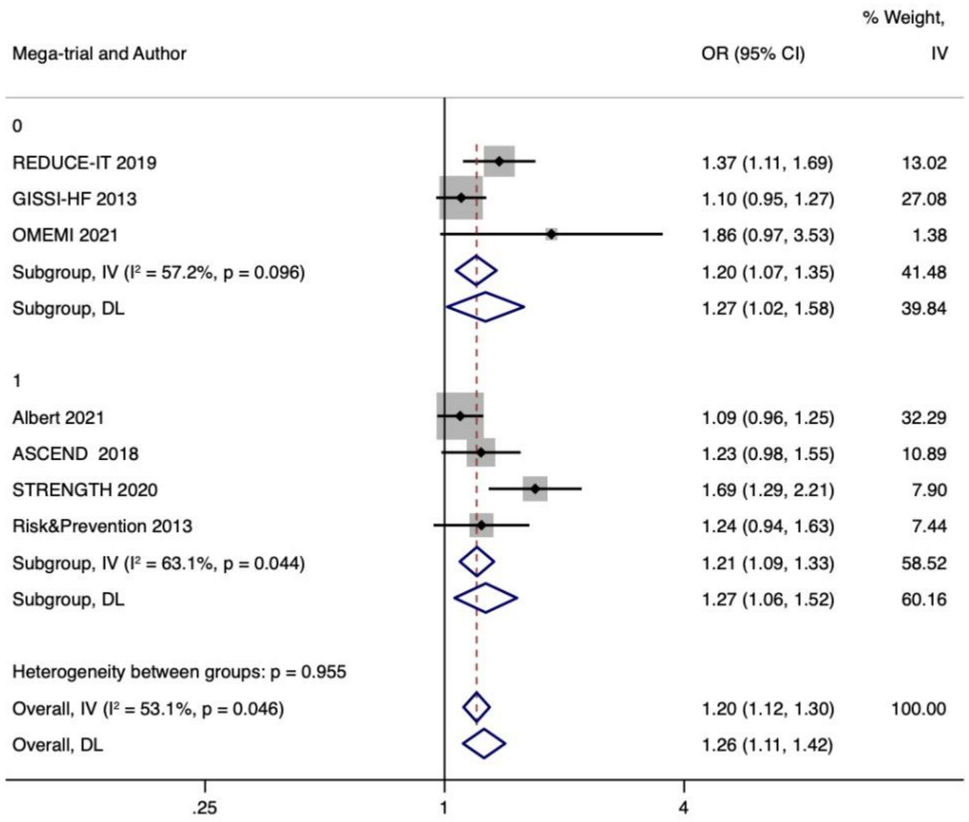

© 2024 Kastrati L et al. *JAMA Network Open.*

12. NISSEN 2016

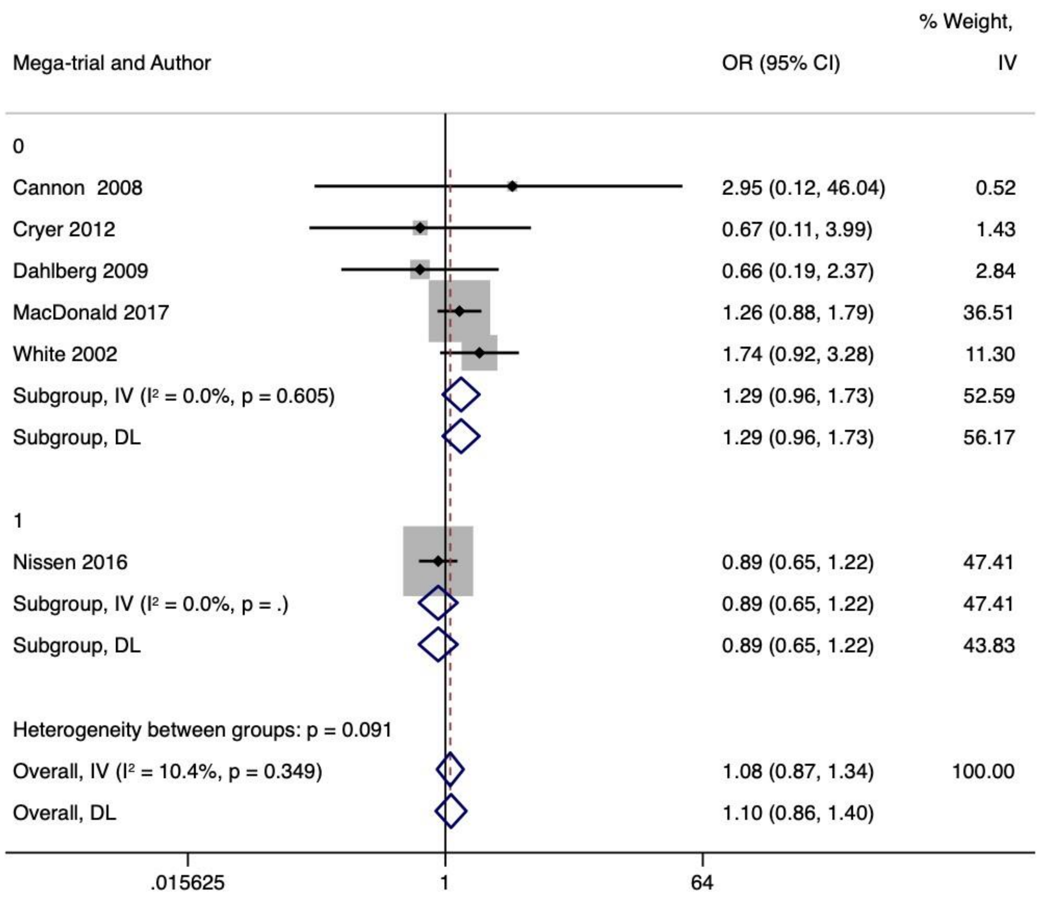

© 2024 Kastrati L et al. *JAMA Network Open.*

13. PROFESS 2008

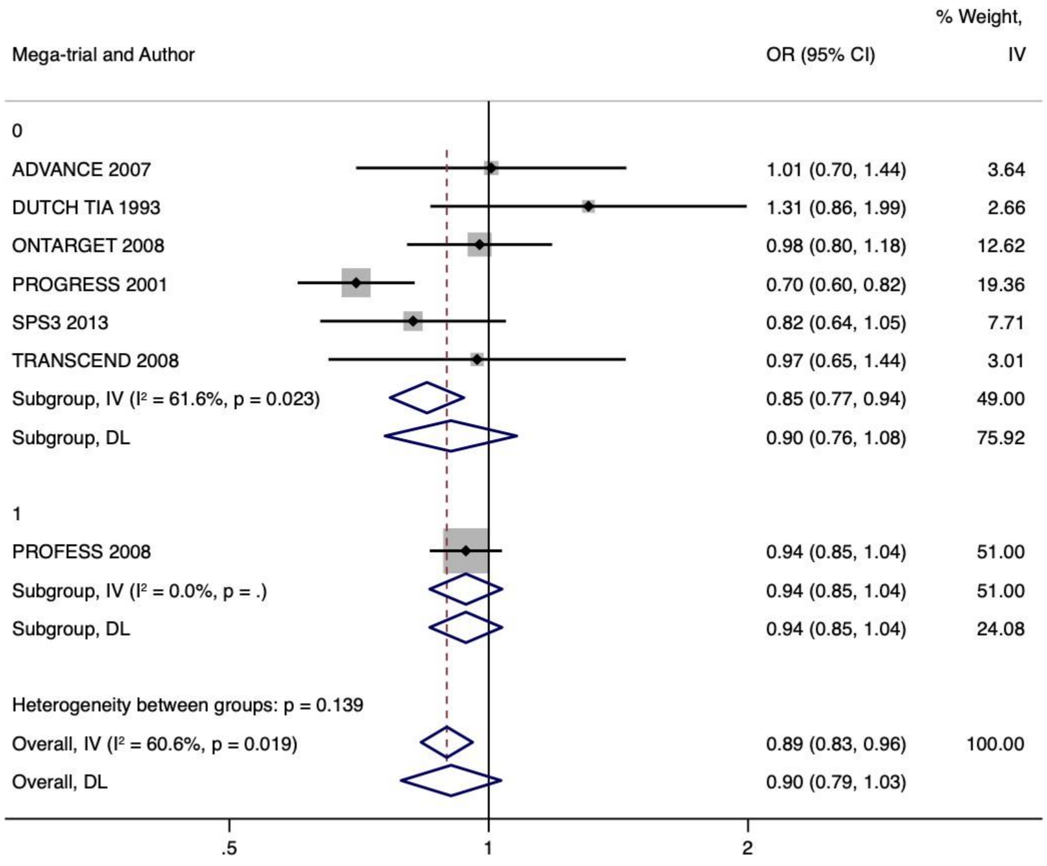

© 2024 Kastrati L et al. *JAMA Network Open.*

14. EXTRACT TIMI 25 2006

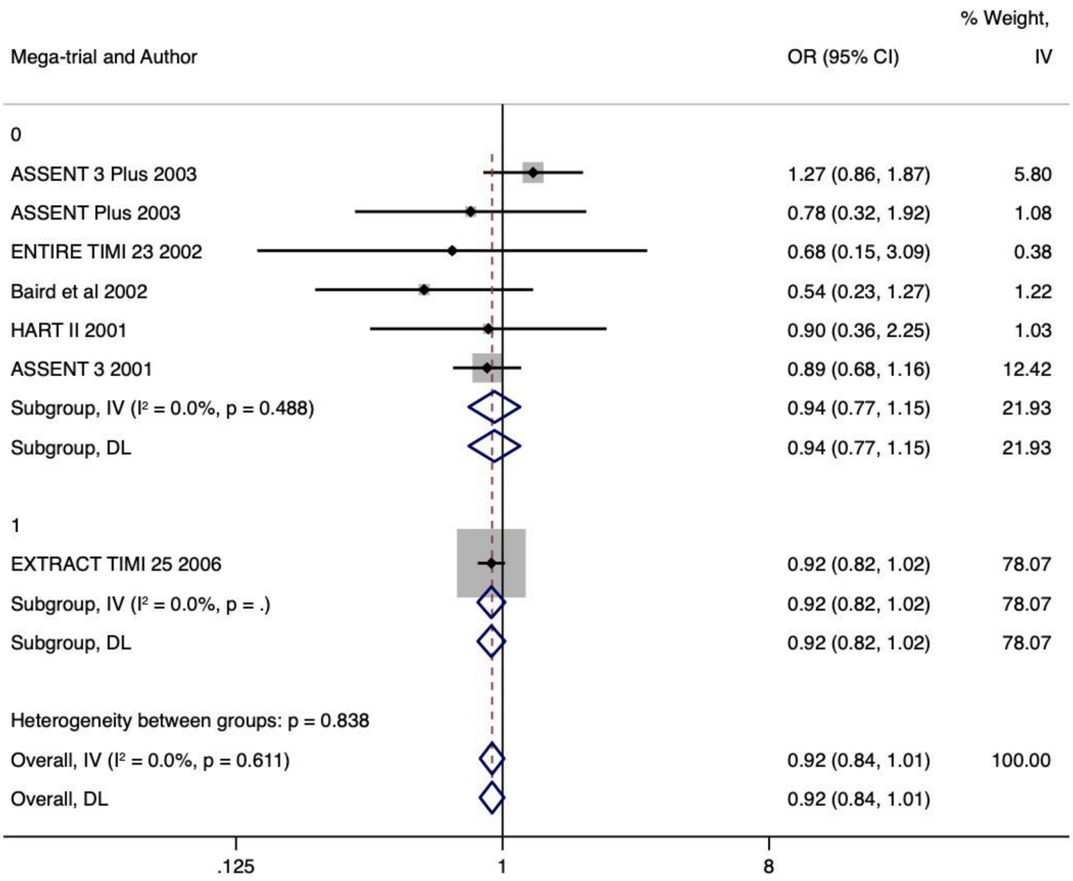

© 2024 Kastrati L et al. *JAMA Network Open.*

15. ODYSSEY OUTCOMES 2019

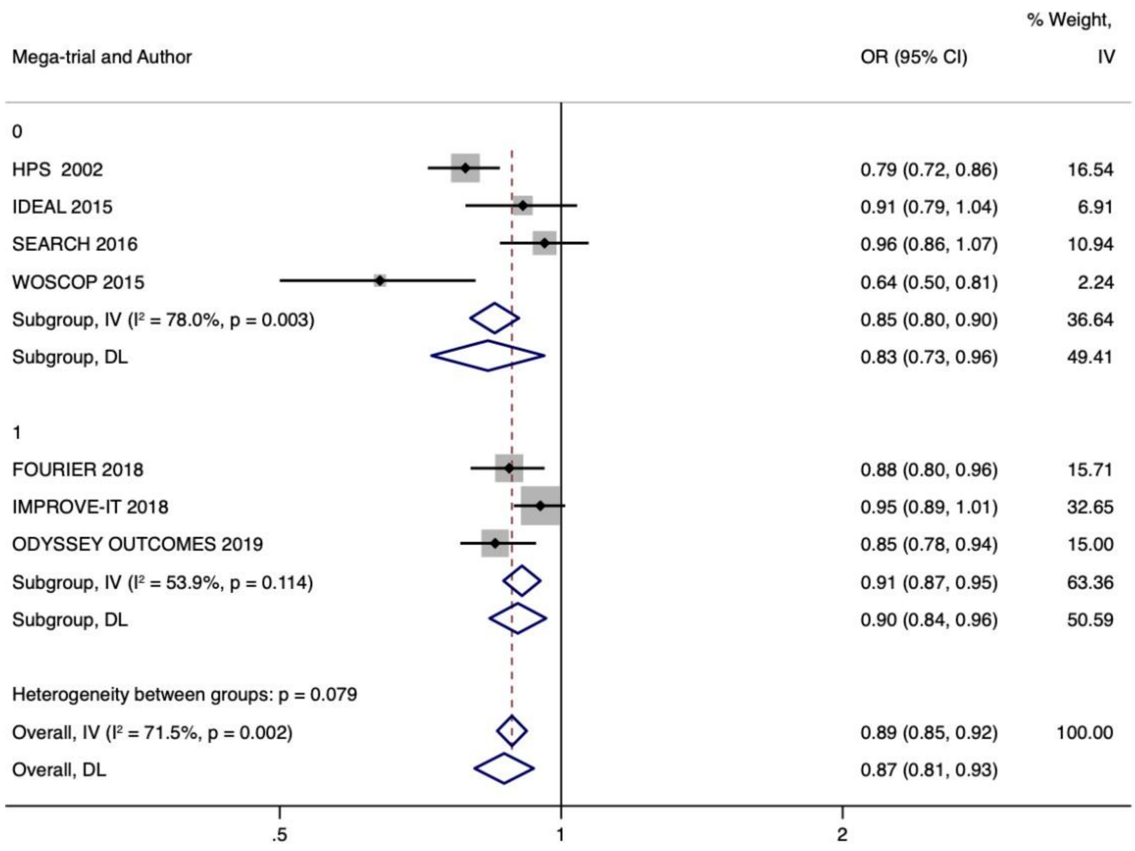

© 2024 Kastrati L et al. *JAMA Network Open.*

16. BaSICS 2021

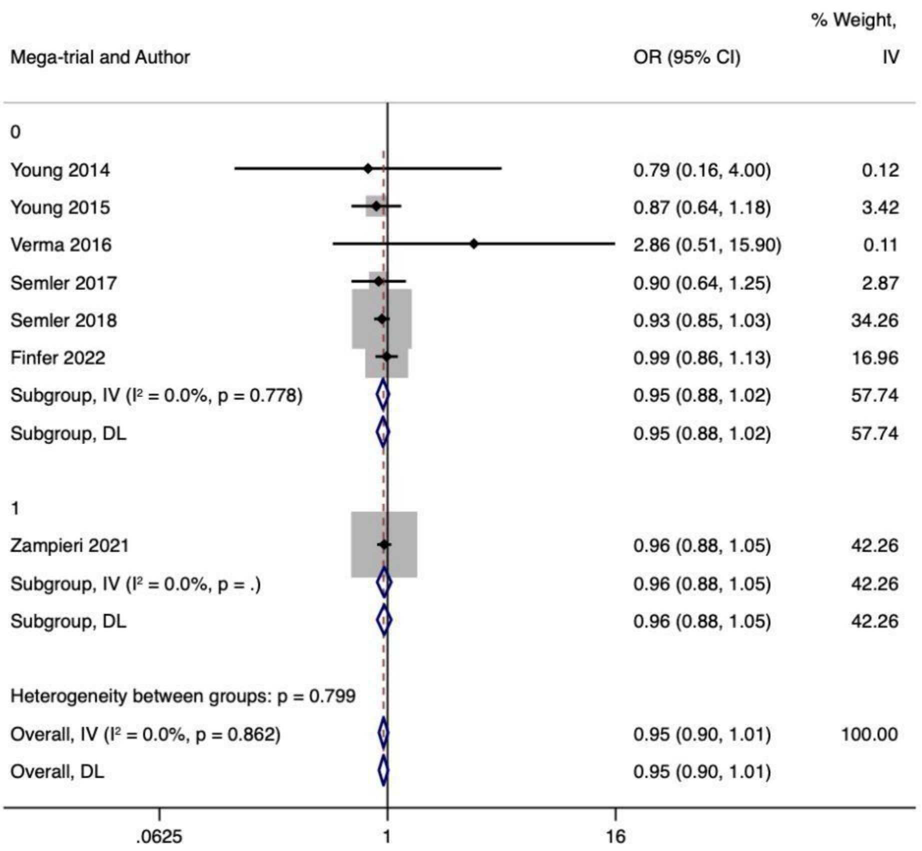

© 2024 Kastrati L et al. *JAMA Network Open.*

17. BEAUTIFUL trial 2008

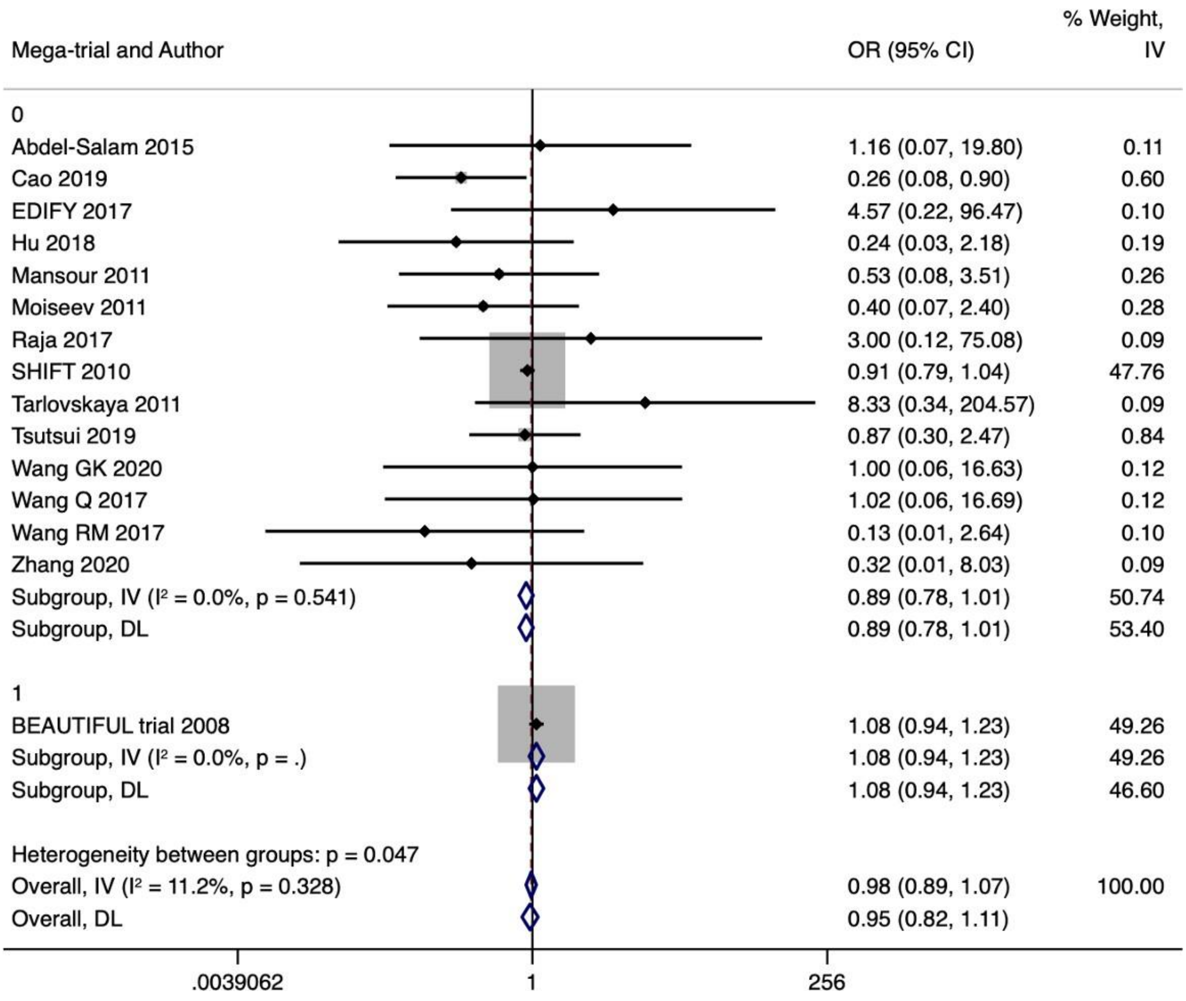

© 2024 Kastrati L et al. *JAMA Network Open.*

18. SU.VI.MAX 2004

Mega-trial and Author	OR (95% Cl)	% Weight, IV
0		
Cook2007	1.02 (0.91, 1.14)	14.89
Kataja-Tuomola 2010	1.40 (0.95, 2.05)	1.30
Catalano 2007	1.53 (0.67, 3.51)	0.28
Rapola 1998	1.10 (0.78, 1.55)	1.63
Rapola 1997	1.19 (0.87, 1.62)	1.98
Subgroup, IV (1^2 = 0.0%, p = 0.448)	1.07 (0.97, 1.18)	20.08
Subgroup, DL	1.07 (0.97, 1.18)	20.95
Hennekens 1996	0.99 (0.91, 1.09)	22.09
SU.VI.MAX 2004	0.98 (0.77, 1.24)	3.31
Lee 1999	1.14 (0.87, 1.49)	2.70
Leppiilii 2000	1.18 (1.00, 1.39)	7.17
Collins 2002	1.00 (0.93, 1.06)	44.66
Subgroup, IV (1^2 = 12.4%, p = 0.335)	1.01 (0.97, 1.06)	79.92
Subgroup, DL	1.02 (0.96, 1.08)	79.05
Heterogeneity between groups: p = 0.332		
Overall, IV (1^2 = 2.2%, p = 0.419)	1.02 (0.98, 1.07)	100.00
Overall, DL	1.03 (0.98, 1.07)	

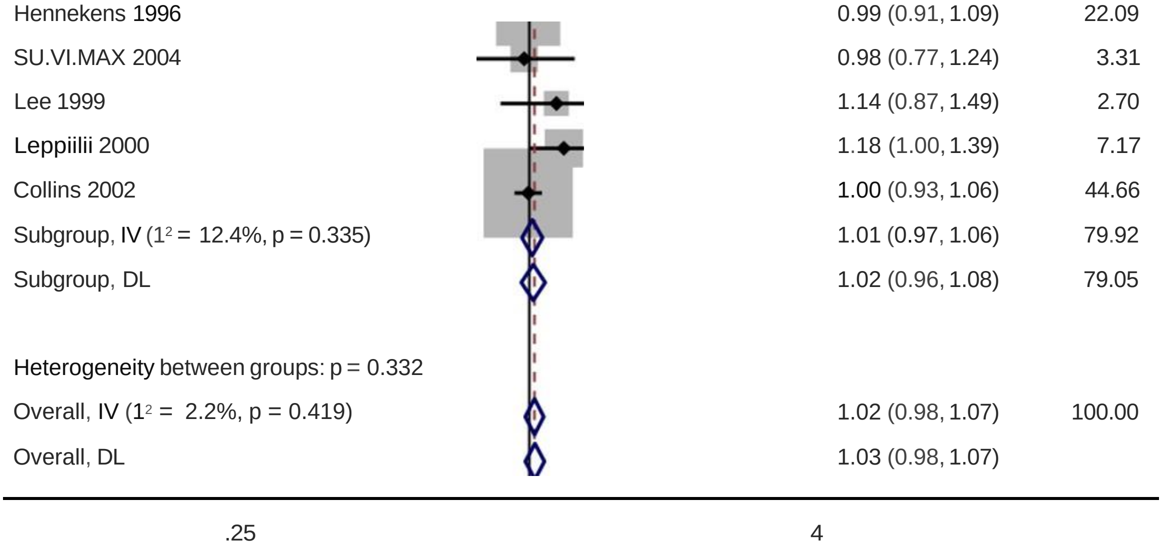

© 2024 Kastrati L et al. *JAMA Network Open.*

19. HPS2-THRIVE 2014

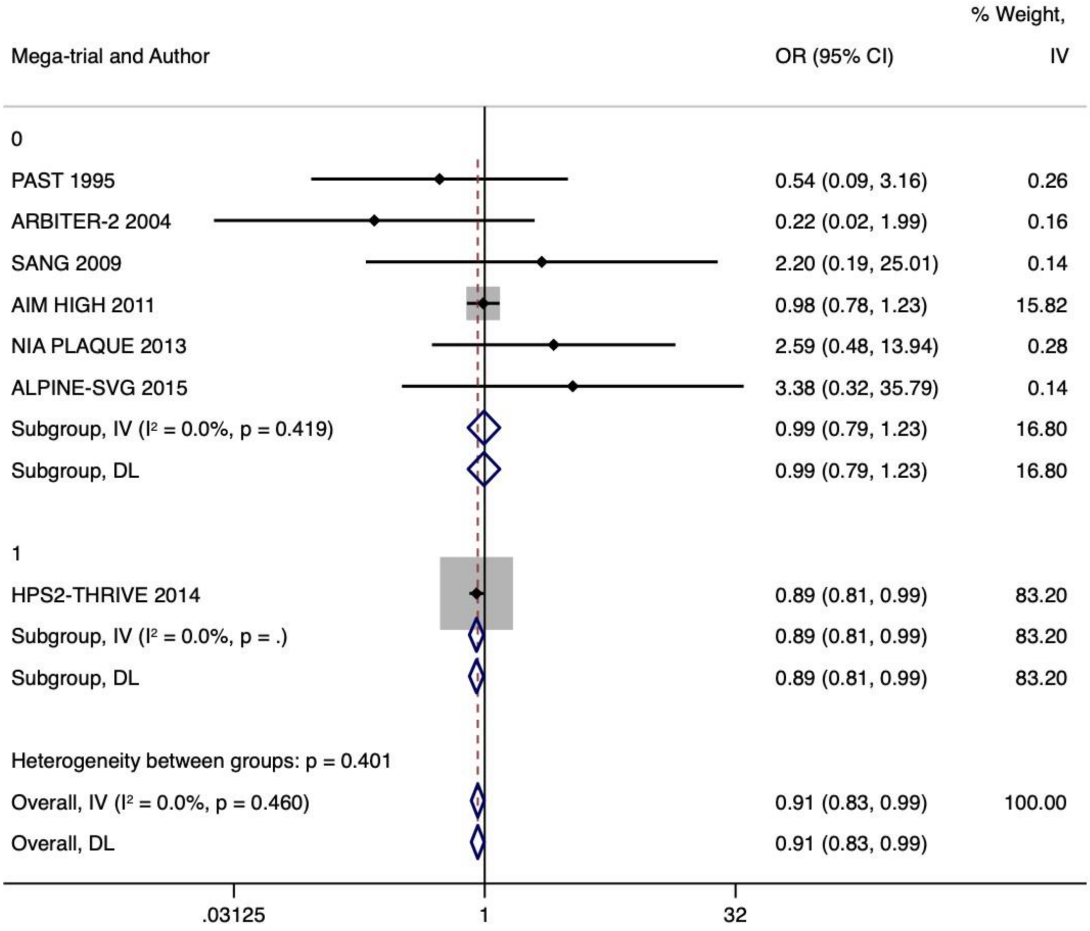

© 2024 Kastrati L et al. *JAMA Network Open.*

20. ILLUMINATE 2007

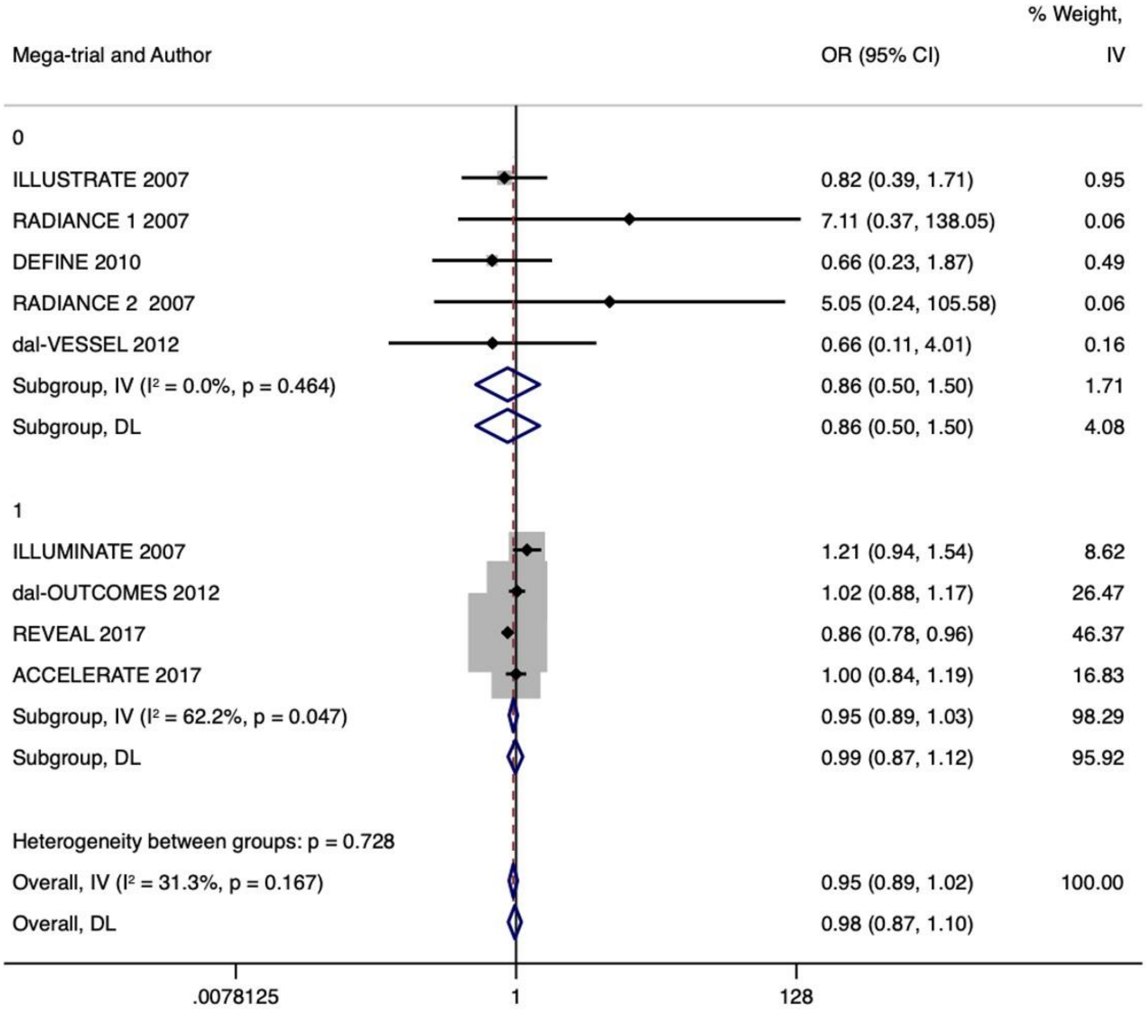

© 2024 Kastrati L et al. *JAMA Network Open.*

21. SAVOR-TIMI 53 2013

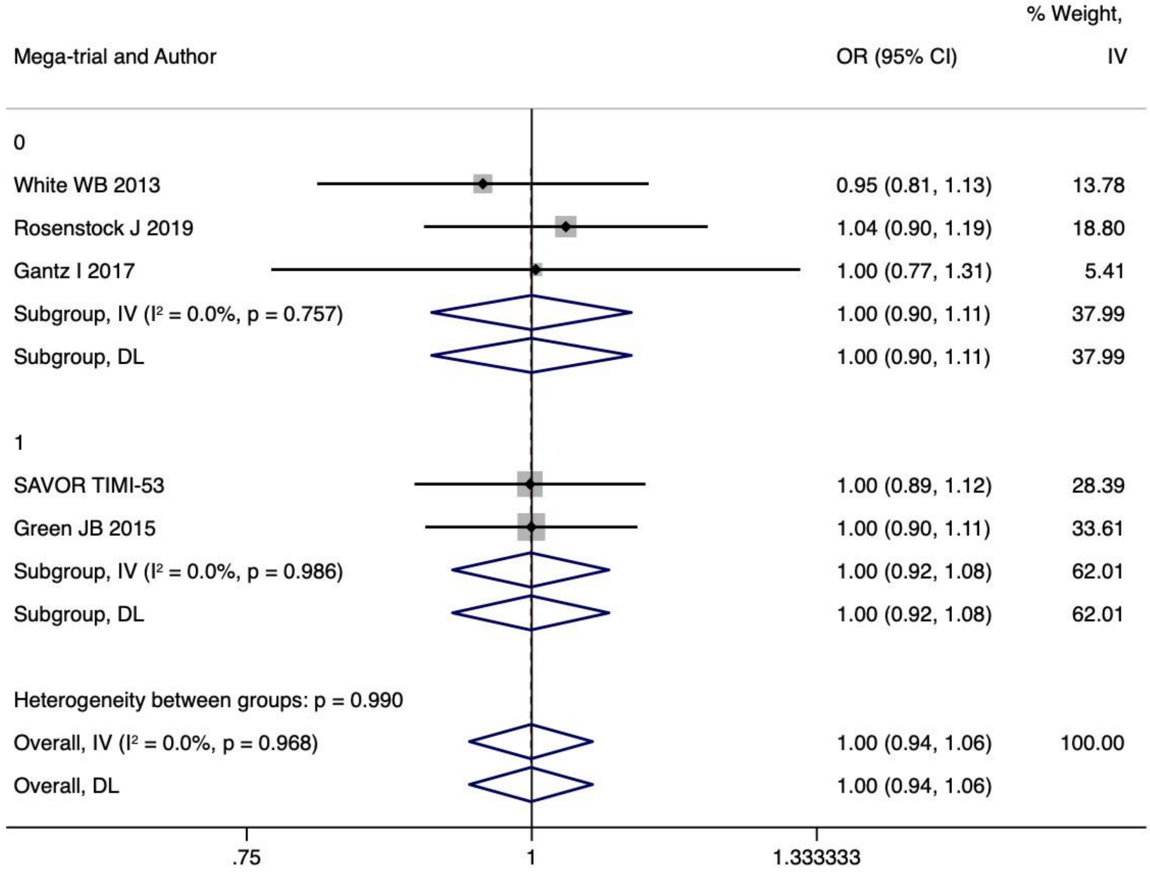

© 2024 Kastrati L et al. *JAMA Network Open.*

22. SCORED 2021

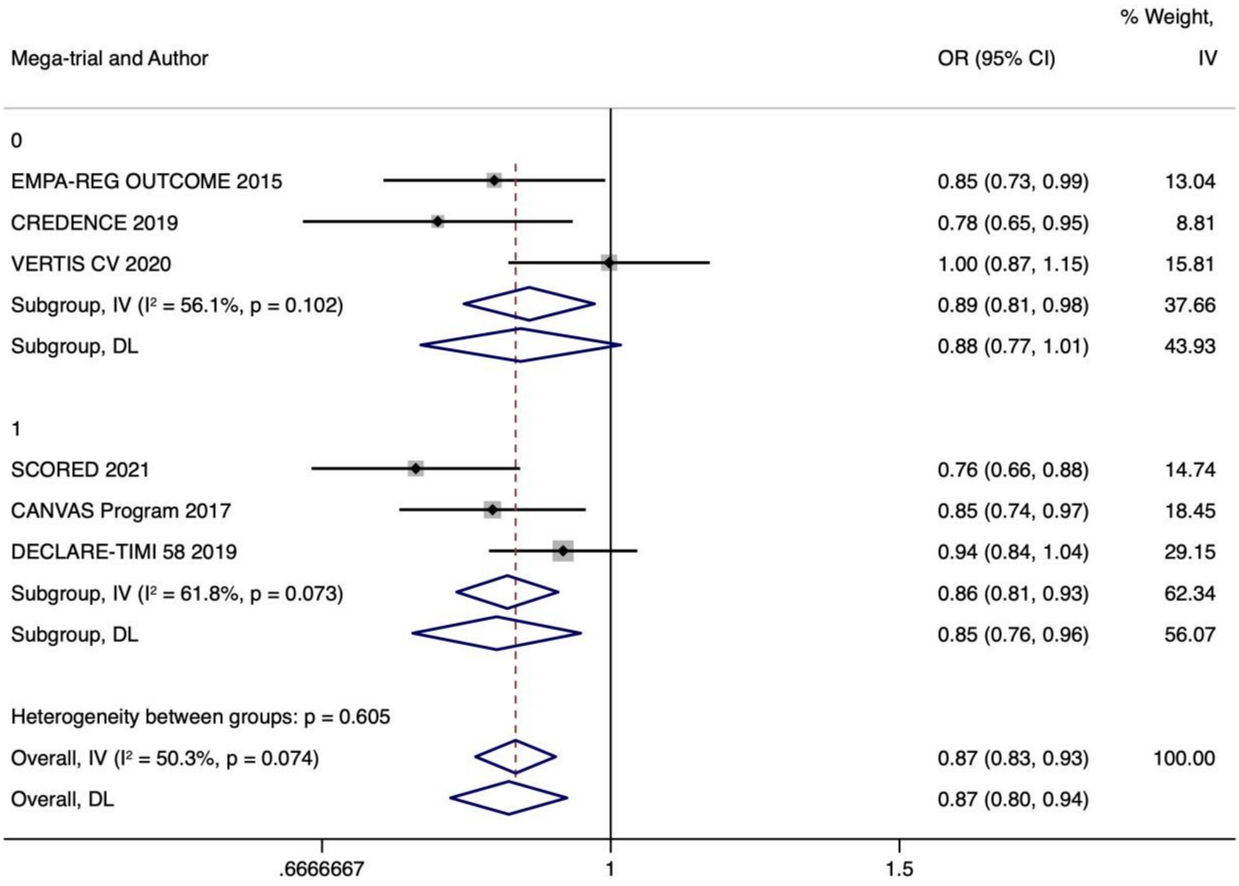

© 2024 Kastrati L et al. *JAMA Network Open.*

23. STABILITY 2017

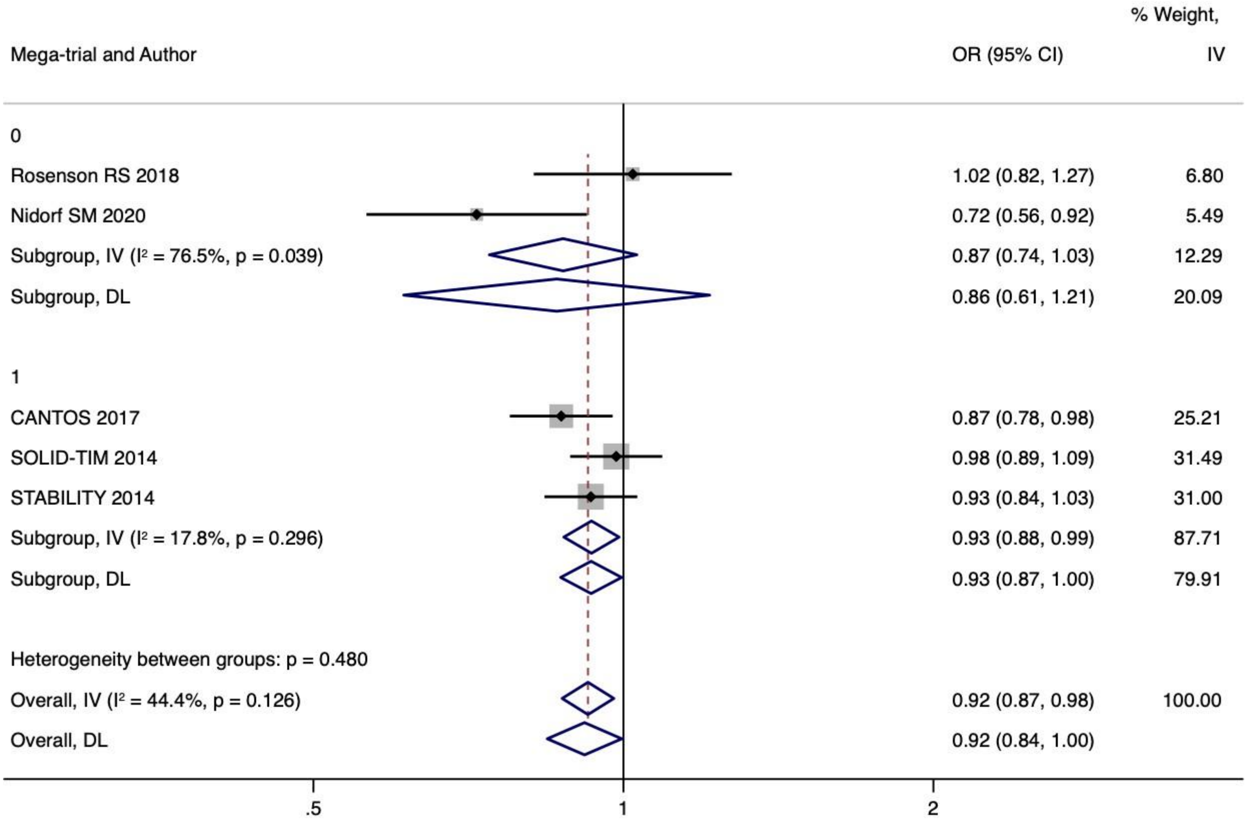

© 2024 Kastrati L et al. *JAMA Network Open.*

24. EXCSCEL 2017

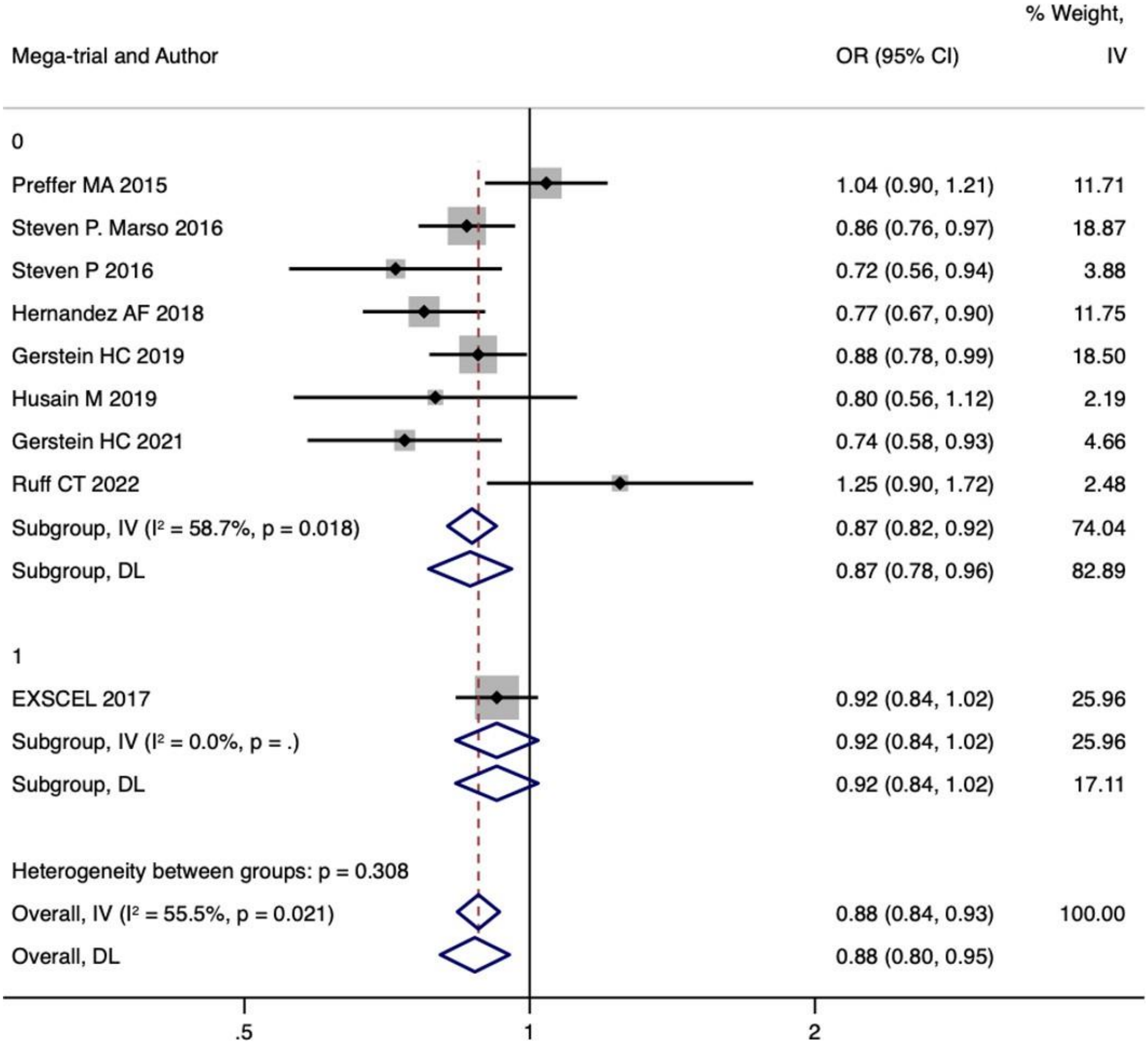

© 2024 Kastrati L et al. *JAMA Network Open.*

25. CURRENT OASIS 7 2010

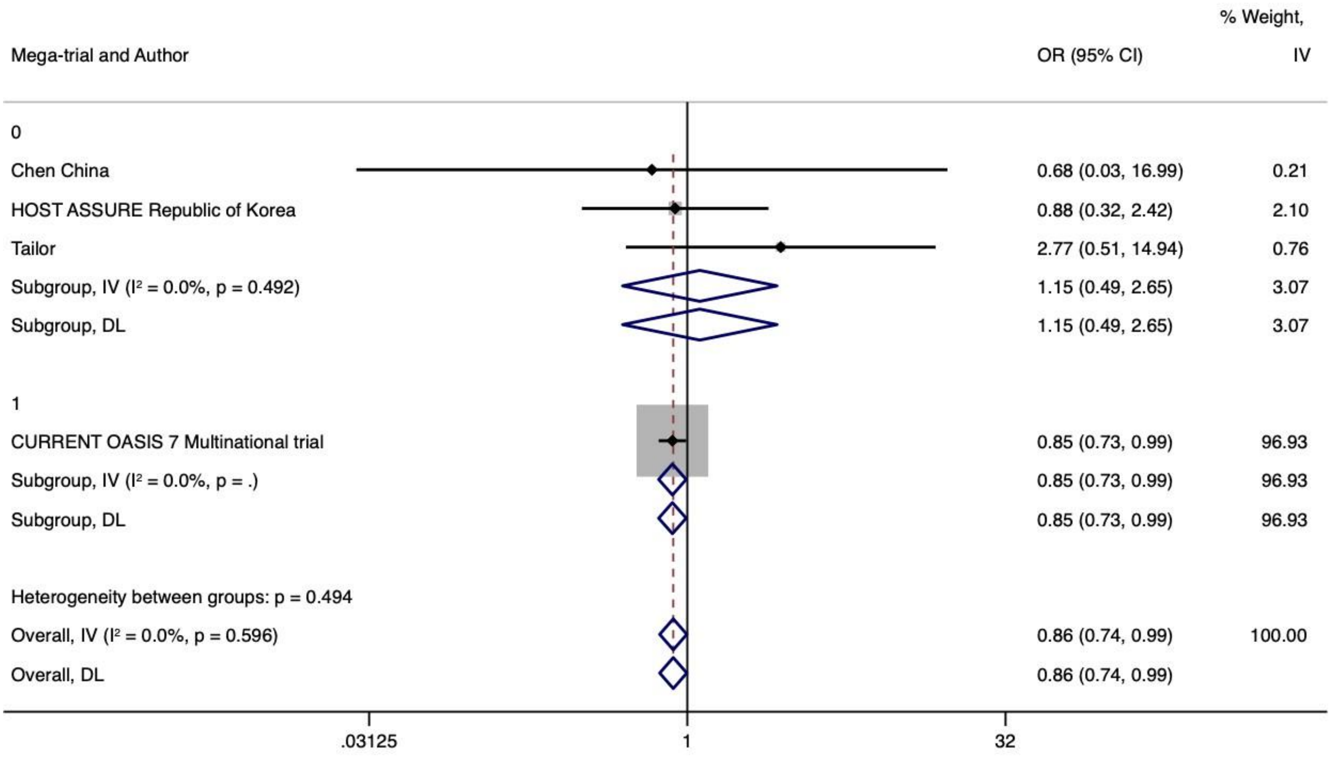

© 2024 Kastrati L et al. *JAMA Network Open.*

26. POISE-2 2014

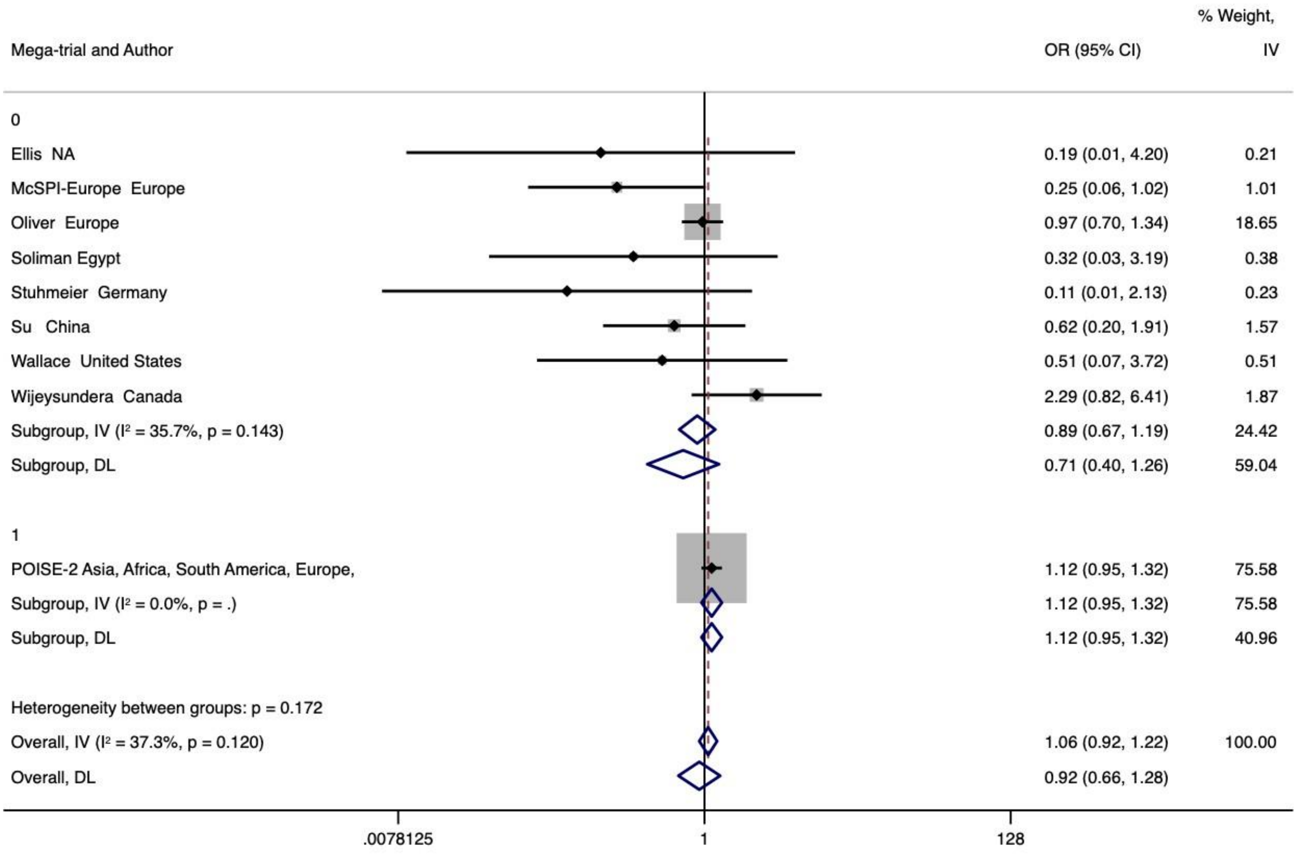

© 2024 Kastrati L et al. *JAMA Network Open.*

27. CHARISMA 2006

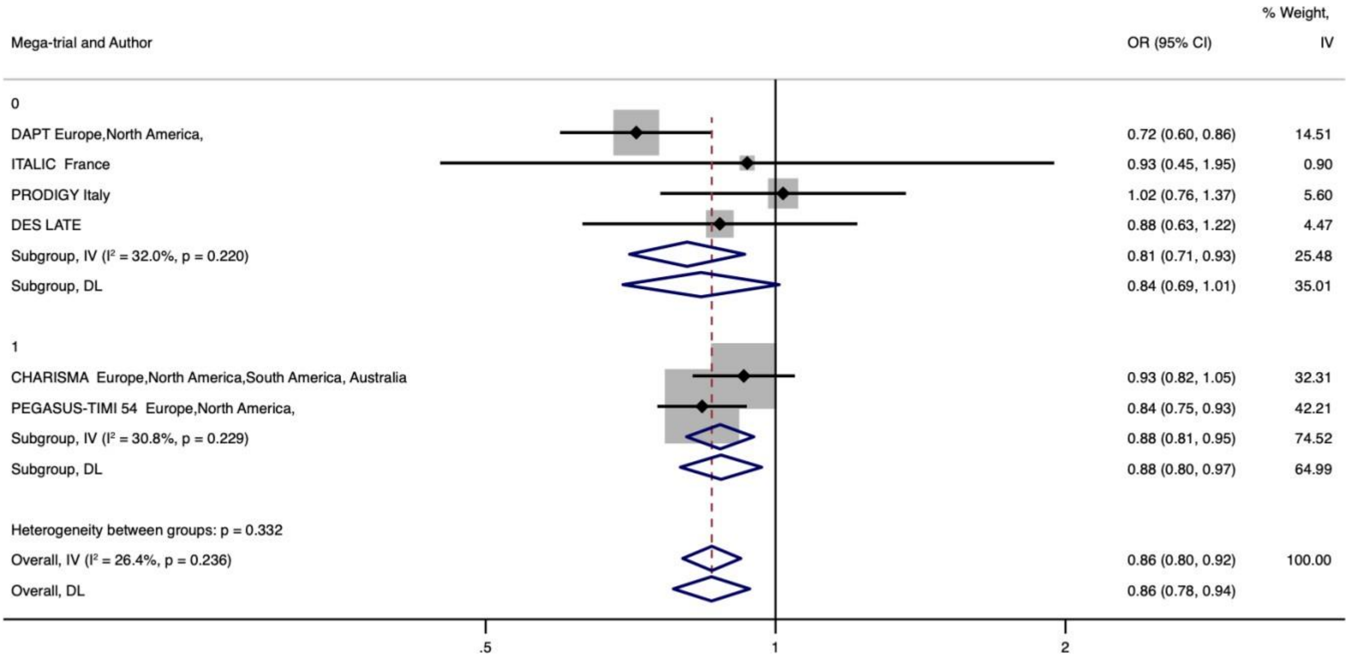

© 2024 Kastrati L et al. *JAMA Network Open.*

28. ENGAGE TIMI 2013

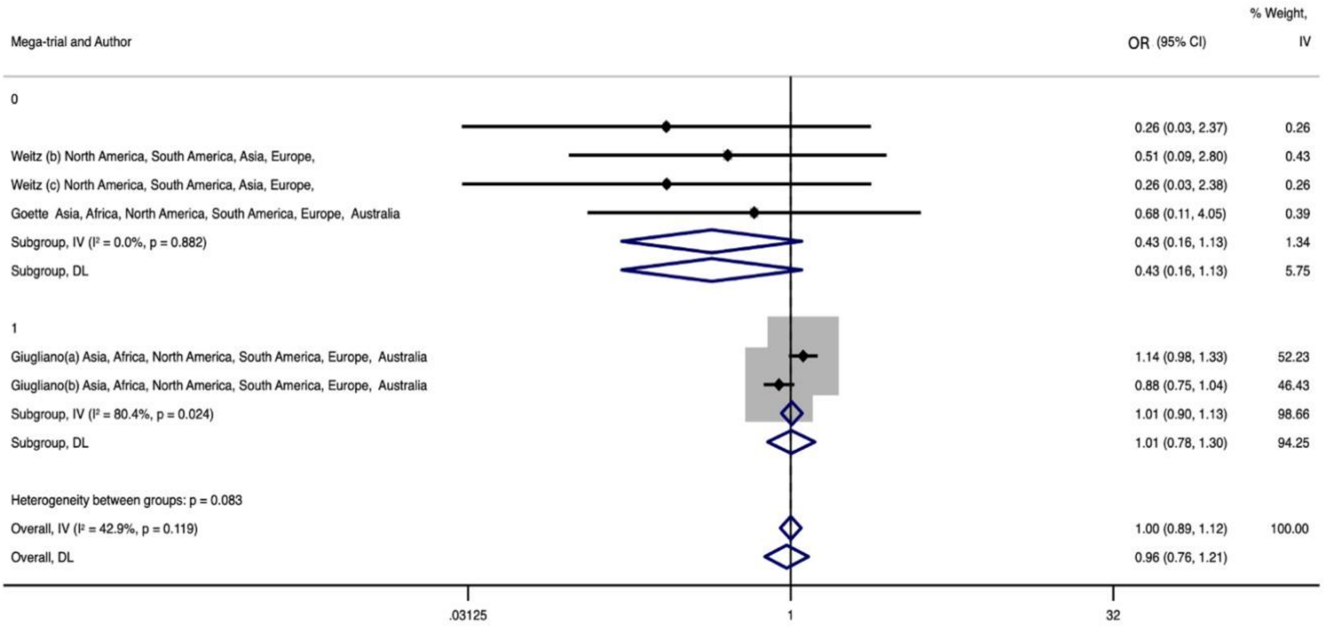

© 2024 Kastrati L et al. *JAMA Network Open.*

29. ATLAS 2012

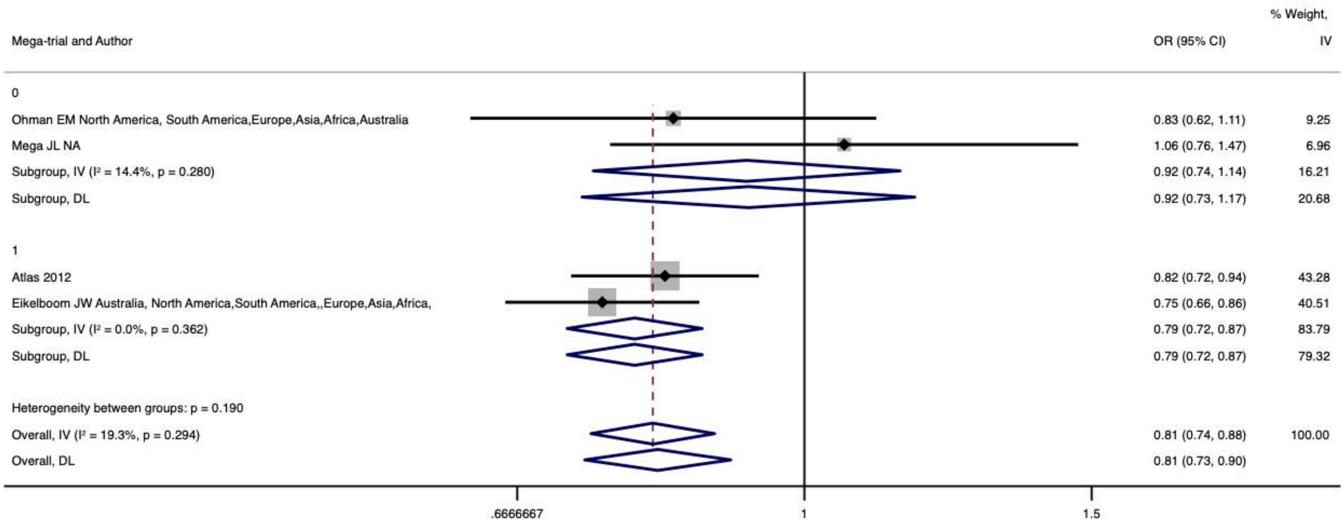

© 2024 Kastrati L et al. *JAMA Network Open.*

30. CAMELLIA-TIMI 2018

Mega-trial and Author	OR(95%CI)	%Weight, IV
0		
le Roux 2017	0.50 (0.03, 7.95)	0.19
greenway 2010	1.50 (0.06, 36.67)	0.14
Nissen 2016	0.96 (0.75, 1.23)	24.11
Apovian 2013	1.49 (0.06, 36.60)	0.14
Bakris 2002	1.85 (0.17, 20.16)	0.26
Allison 2012	2.05 (0.08, 50.24)	0.14
Davies 2015	1.00 (0.09, 11.02)	0.26
Gadde 2011	5.99 (0.32, 111.17)	0.17
Hollander 2013	1.53 (0.06, 37.28)	0.14
Fidler 2011	4.66 (0.24, 90.25)	0.17
Subgroup, IV (I'=0.0%, p = 0.944)	1.00 (0.78, 1.27)	25.73
Subgroup, DL	1.00 (0.78, 1.27)	25.73
CAMELLIA TIMI 2018	0.99 (0.86, 1.14)	74.27
Subgroup, IV (I'=0.0%, p = .)	0.99 (0.86, 1.14)	74.27
Subgroup, DL	0.99 (0.86, 1.14)	74.27
Heterogeneity between groups: p = 0.968		
Overall, IV (1^2 = 0.0%, p = 0.969)	0.99 (0.88, 1.12)	100.00
Overall, DL	0.99 (0.88, 1.12)	

.0078125 128

© 2024 Kastrati L et al. *JAMA Network Open.*

31. SOLID-TIMI 52 2014

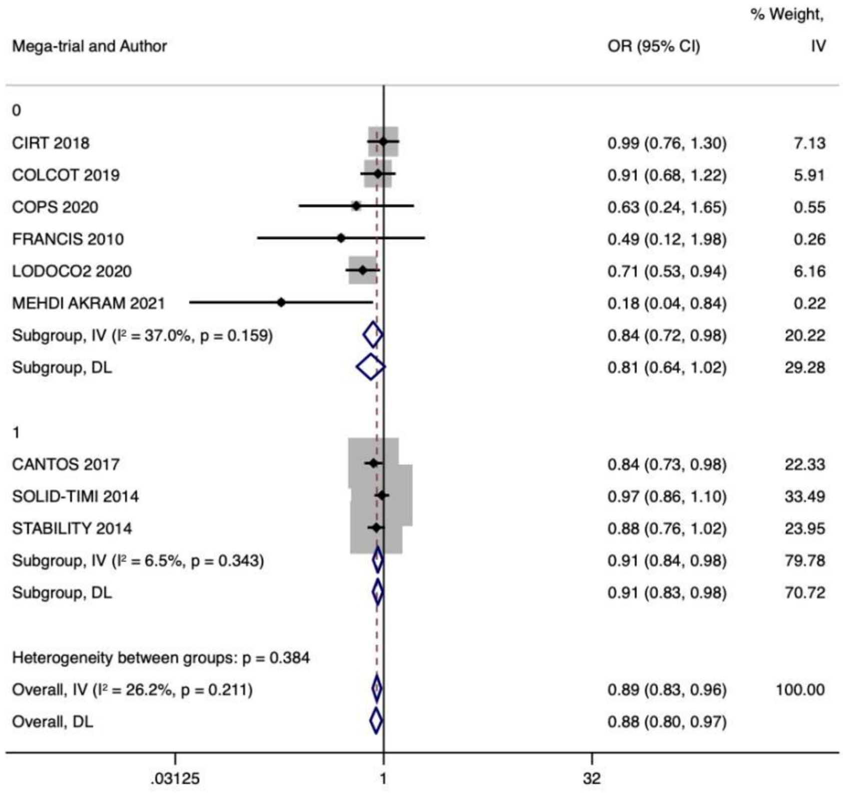

© 2024 Kastrati L et al. *JAMA Network Open.*

32. ONTARGET 2008

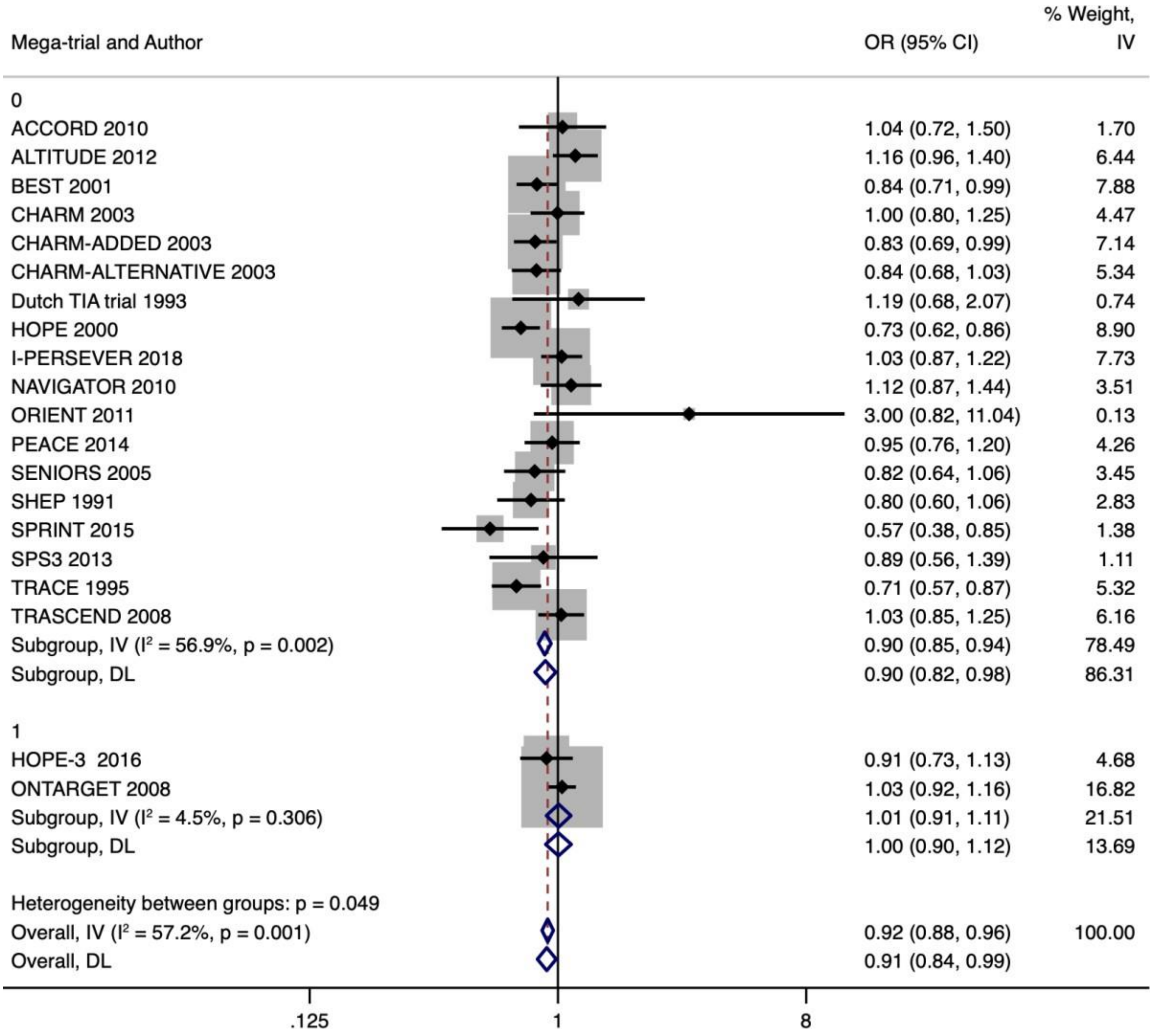

© 2024 Kastrati L et al. *JAMA Network Open.*

33. Wallentin 2019

Mega-trial and Author	OR (95%CI)	%Weight, IV
0		
JUMBO-TIMI 2005	0.71 (0.40, 1.24)	1.27
DISPERSE-2 2007	0.78 (0.36, 1.68)	0.67
CHAMPION PCI 2009	1.09 (0.91, 1.29)	13.05
CHAMPION-PLATFORM 2009	0.92 (0.74, 1.13)	8.99
INNOVATE-PC! 2012	1.82 (0.37, 8.82)	0.16
TRILOGY ACS 2012	0.88 (0.73, 1.07)	11.31
Subgroup, IV (1^2 = 0.0%, p = 0.435)	0.95 (0.86, 1.06)	35.46
Subgroup, DL	0.95 (0.86, 1.06)	45.53
TRITON-TIMI 2007	0.75 (0.66, 0.85)	26.05
PLATO 2009	0.84 (0.74, 0.95)	26.98
CHAMPION PHOENIX 2013	0.81 (0.67, 0.97)	11.51
Subgroup, IV (1^2 = 0.0%, p = 0.427)	0.79 (0.73, 0.86)	64.54
Subgroup, DL	0.79 (0.73, 0.86)	54.47
Heterogeneity between groups: p = 0.006		
Overall, IV (1^2 = 42.8%, p = 0.082)	0.85 (0.80, 0.90)	100.00
Overall, DL	0.86 (0.78, 0.95)	

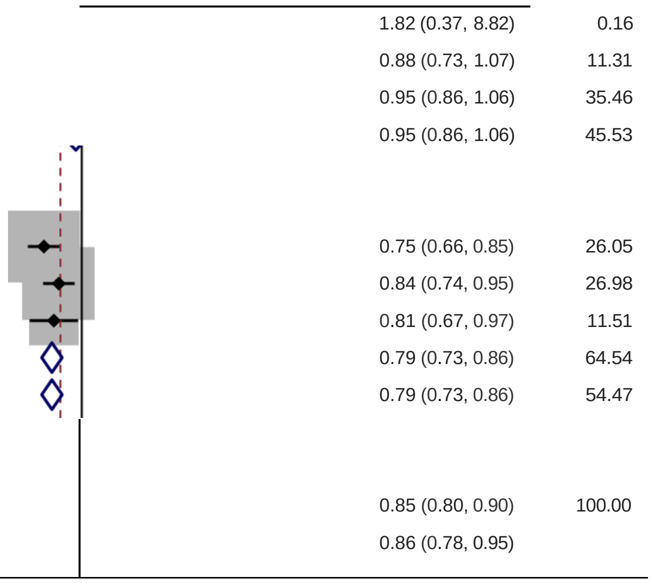

.125 8

© 2024 Kastrati L et al. *JAMA Network Open.*

34. COMMIT 2005

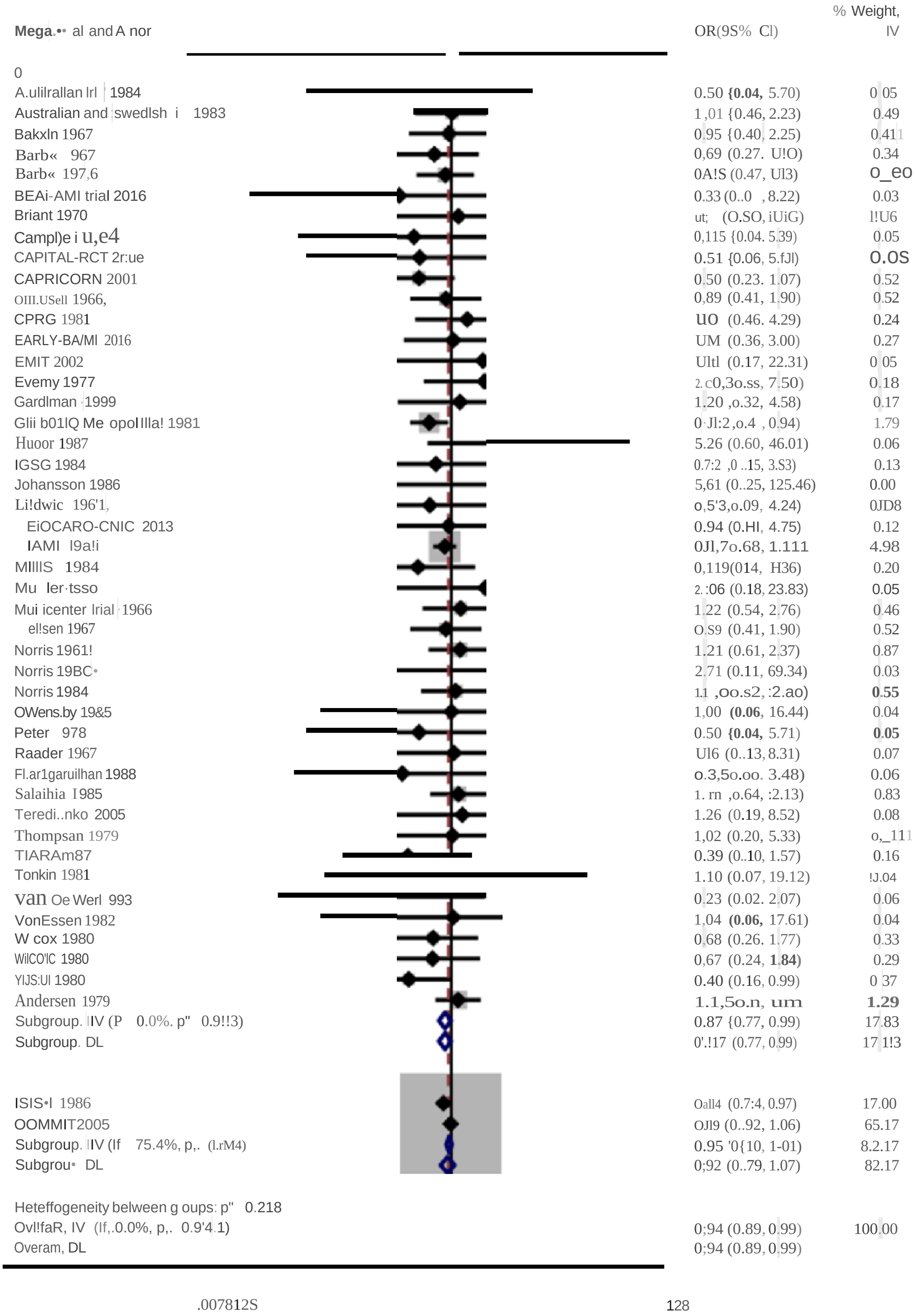

© 2024 Kastrati L et al. *JAMA Network Open.*

35. SIGNIFY 2014

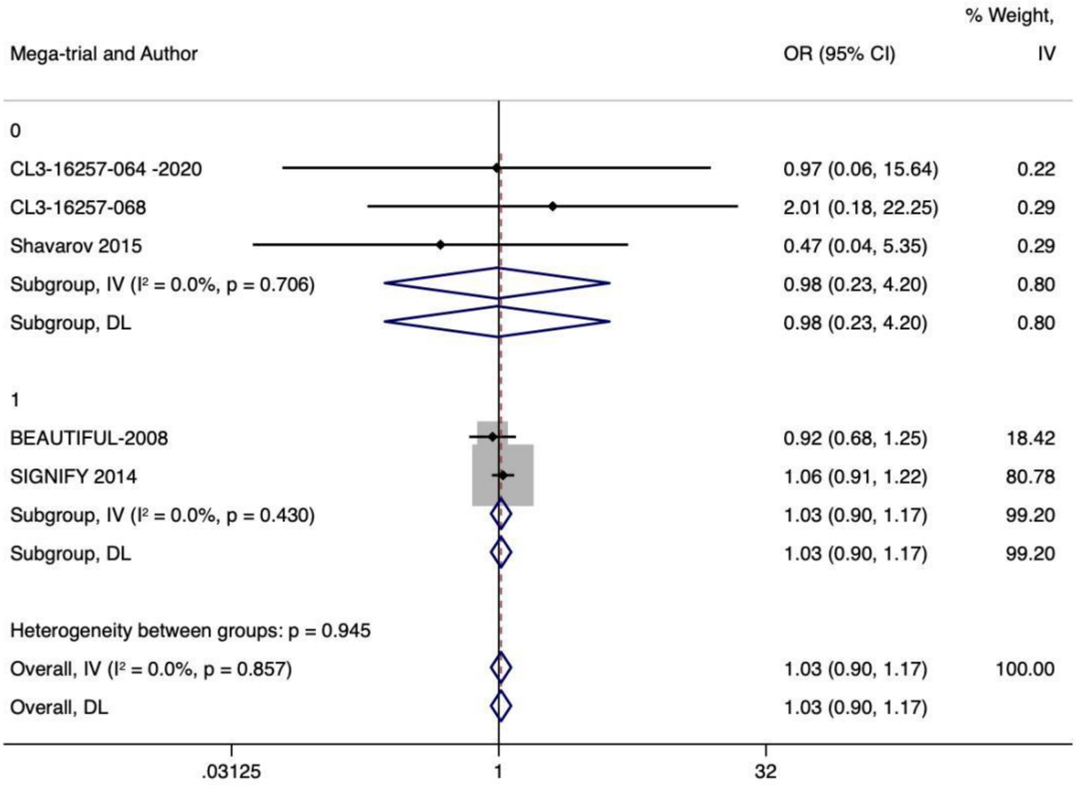

© 2024 Kastrati L et al. *JAMA Network Open.*

eFigure 1. Agreement Between Mega-Trials and Smaller Trials for Primary Outcome: Random Effects (DerSimonian Laird)

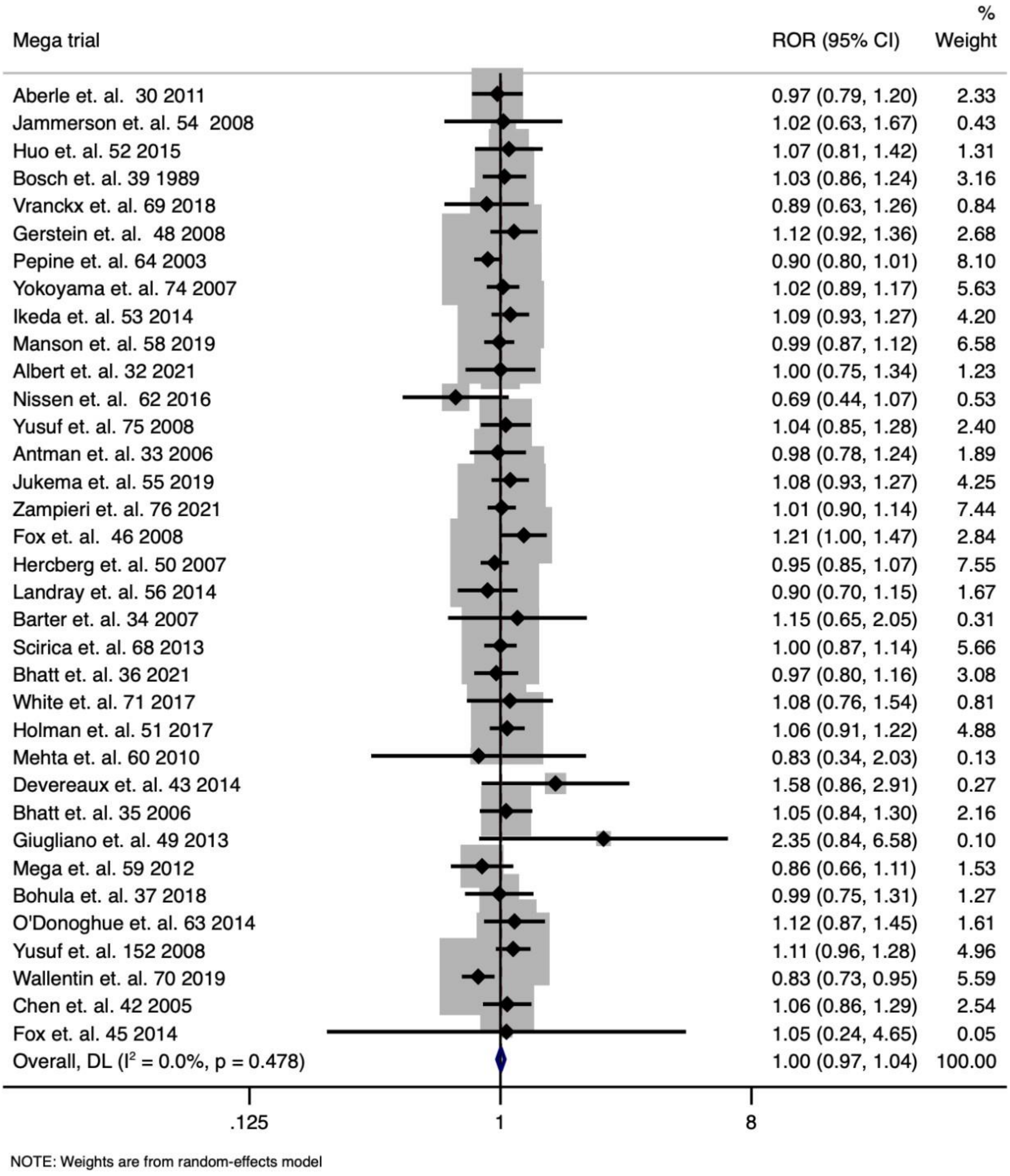

© 2024 Kastrati L et al. *JAMA Network Open.*

eAppendix 5. Meta-Analyses of Mega-Trials vs Smaller Trials for All-Cause Mortality

1. **JUPITER 2008**

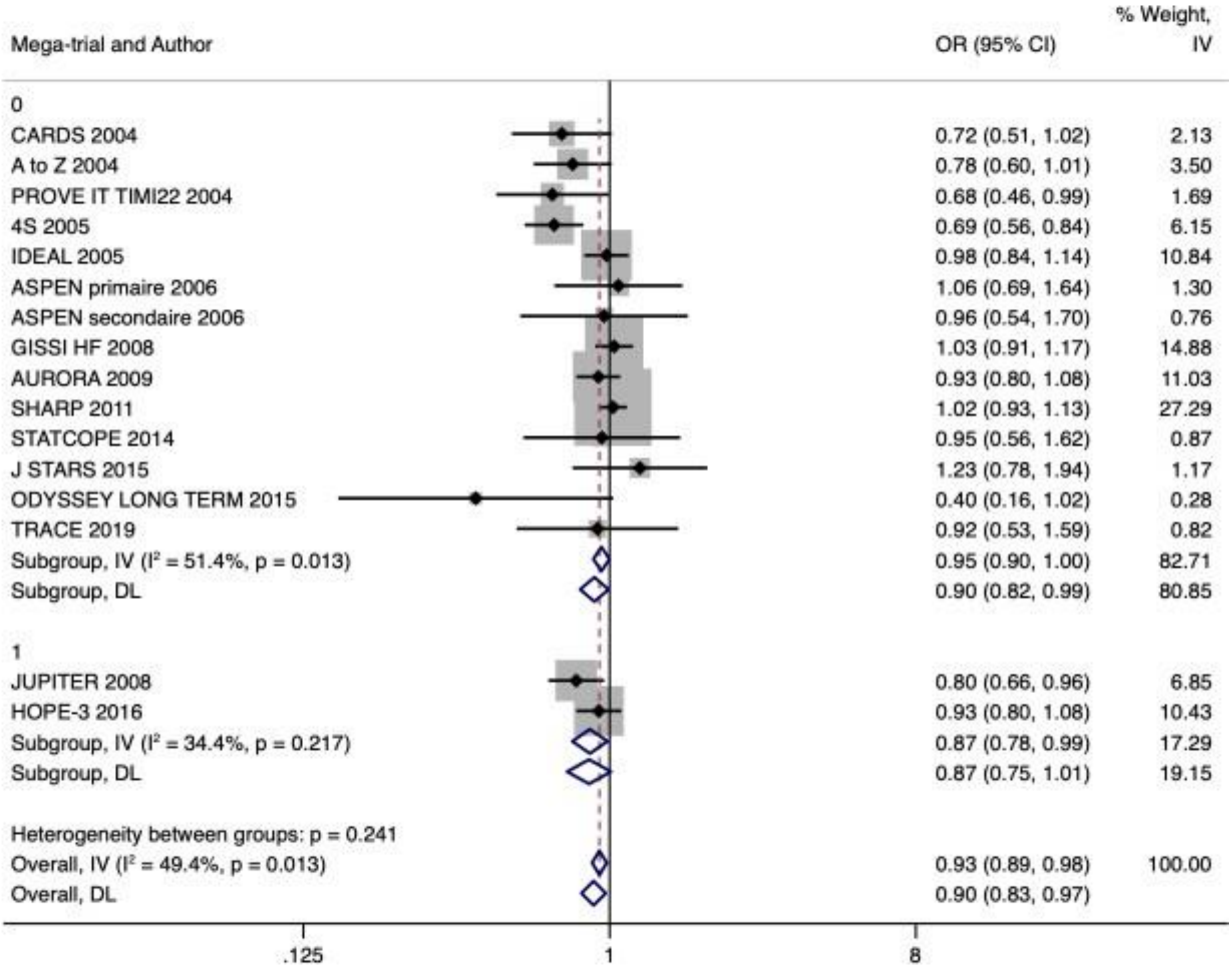

© 2024 Kastrati L et al. *JAMA Network Open.*

2. ACCOMPLISH 2008

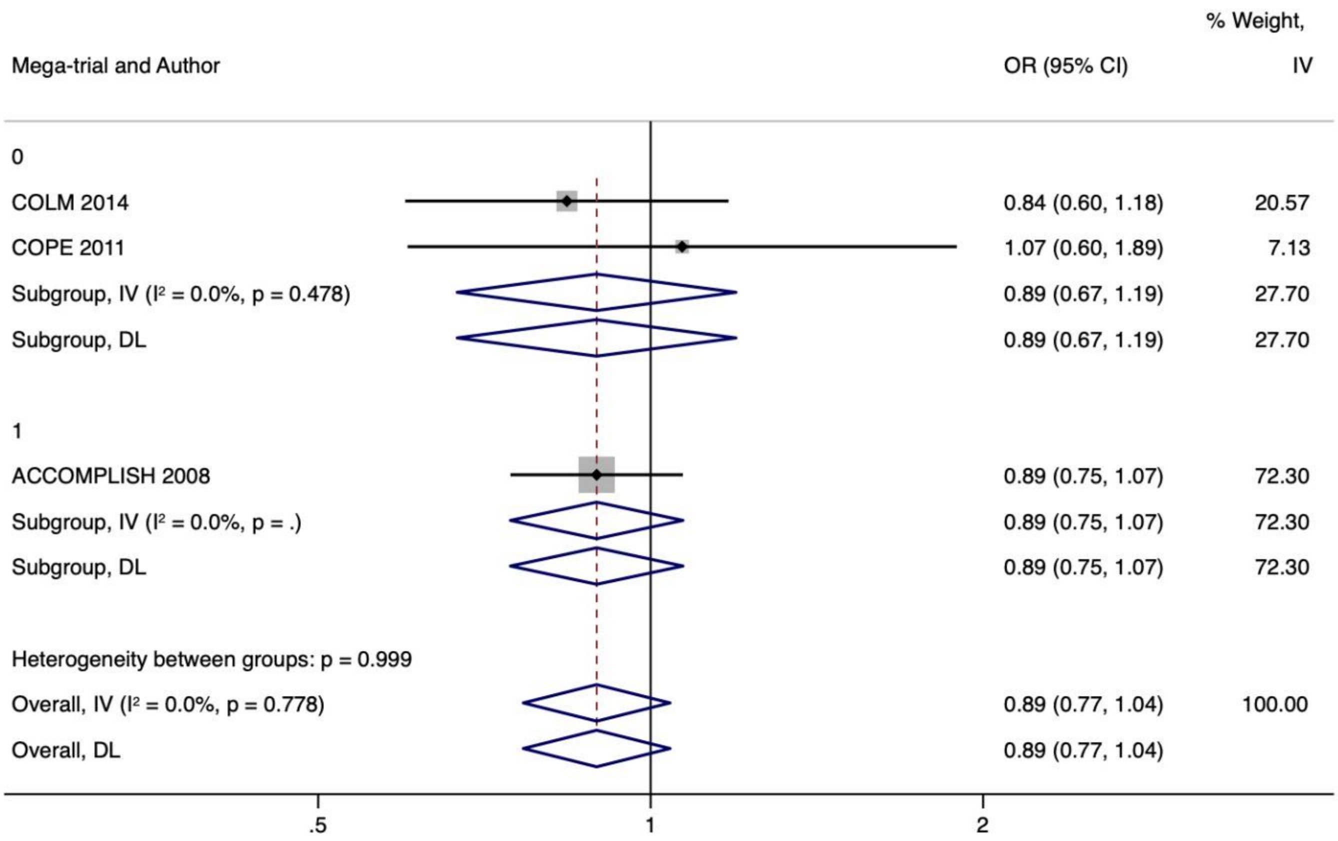

© 2024 Kastrati L et al. *JAMA Network Open.*

3. Huo 2015

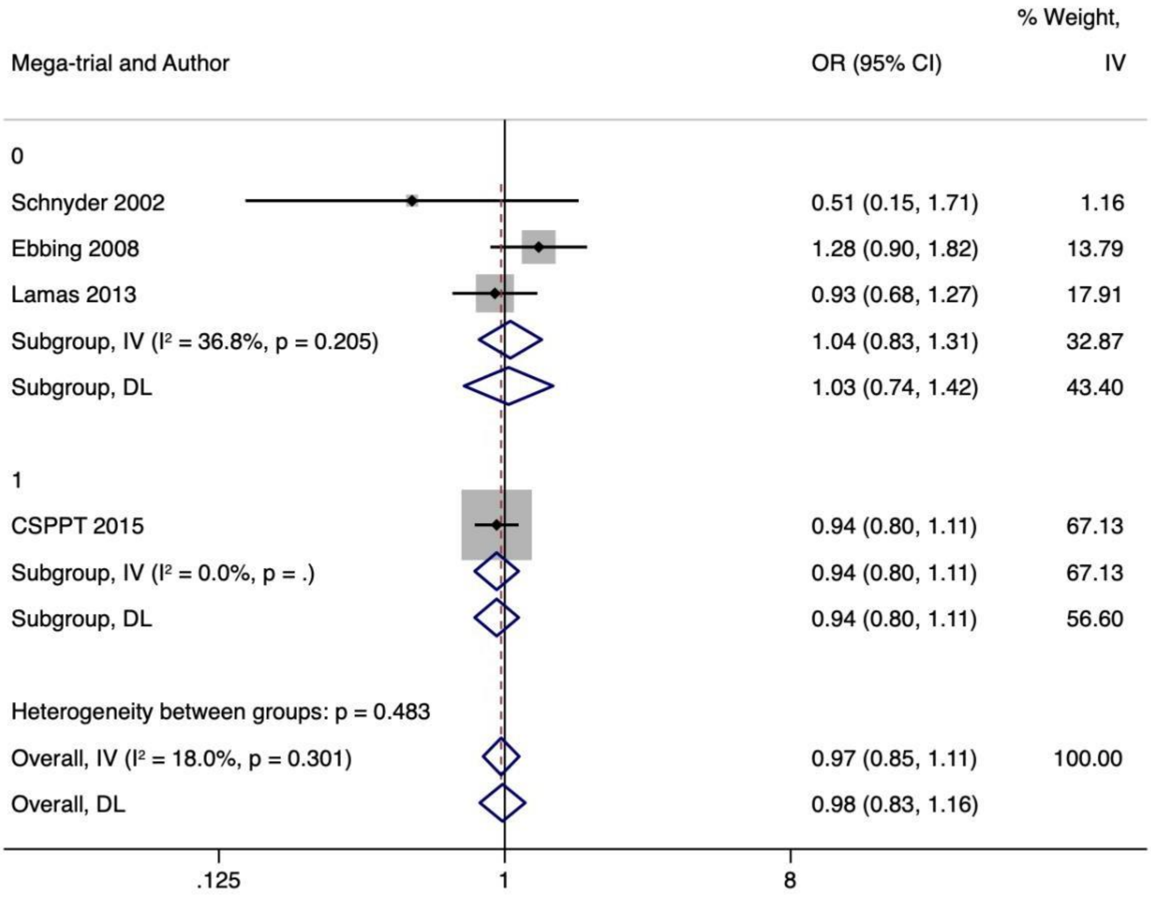

© 2024 Kastrati L et al. *JAMA Network Open.*

4. ORIGIN 2012

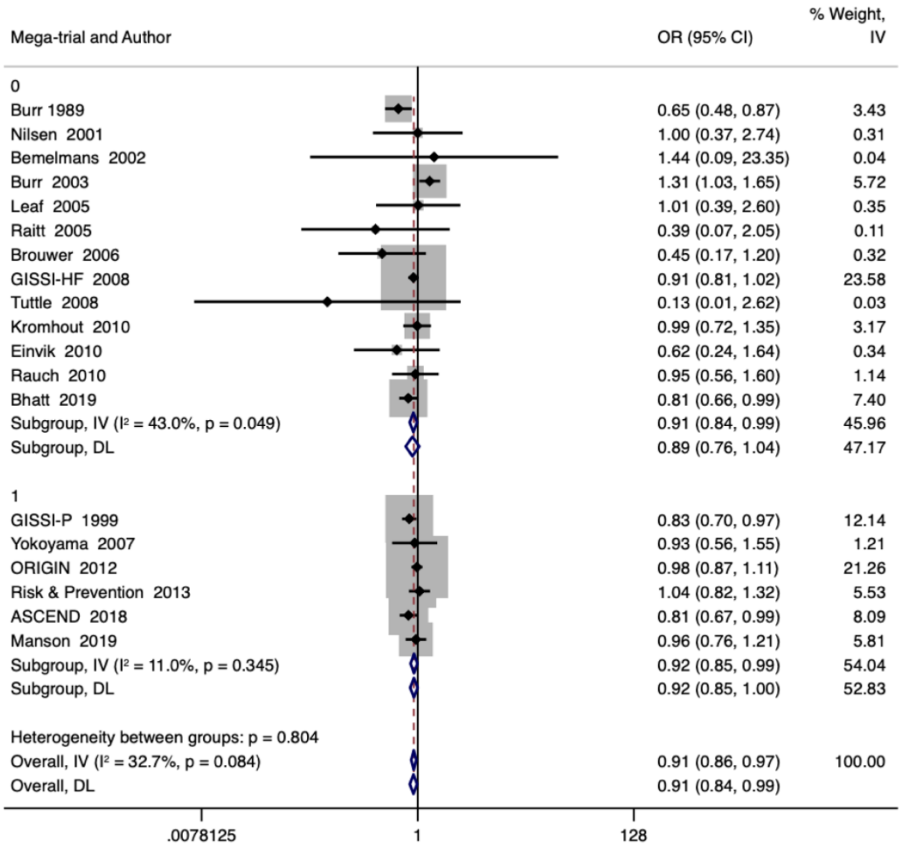

5. **Aberle 2011**

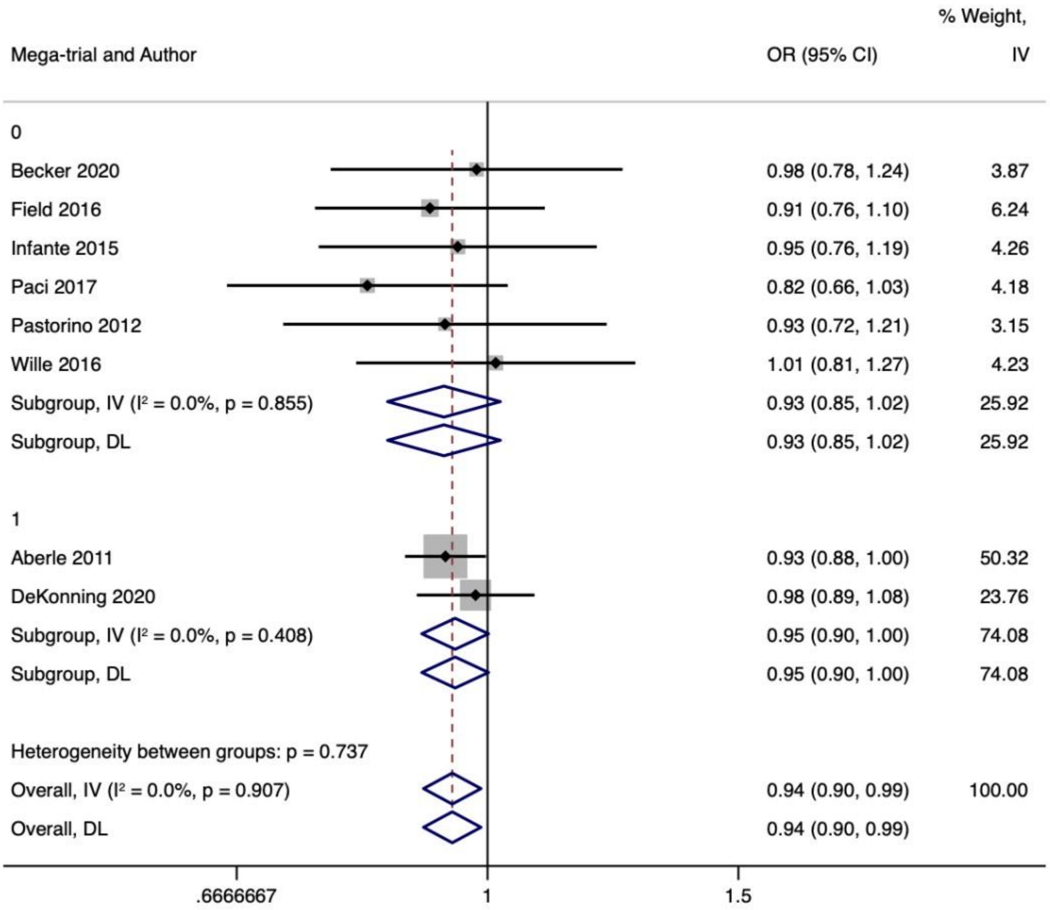

© 2024 Kastrati L et al. *JAMA Network Open.*

6. JPP 2014

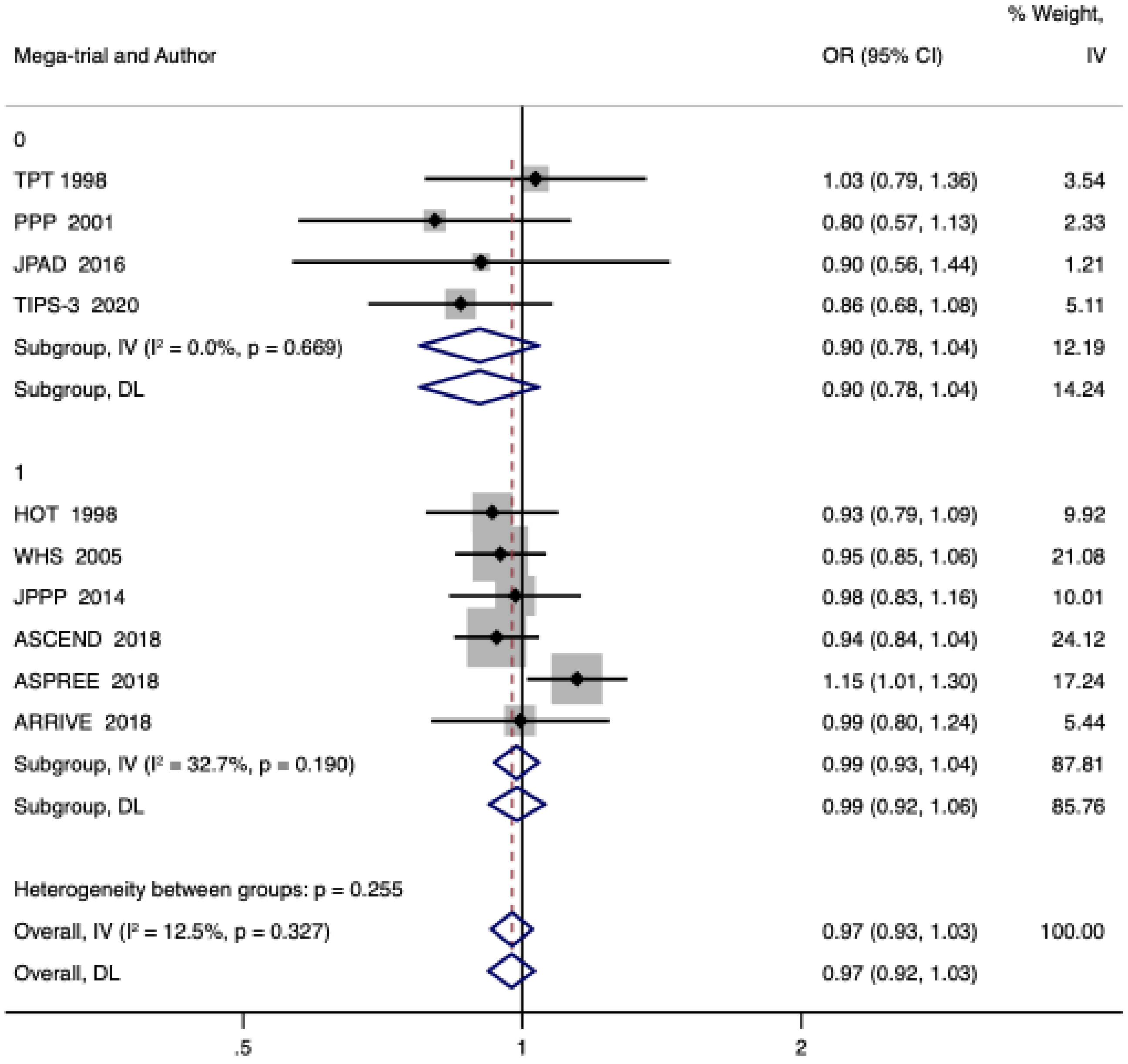

© 2024 Kastrati L et al. *JAMA Network Open.*

7. ACCORD 2008

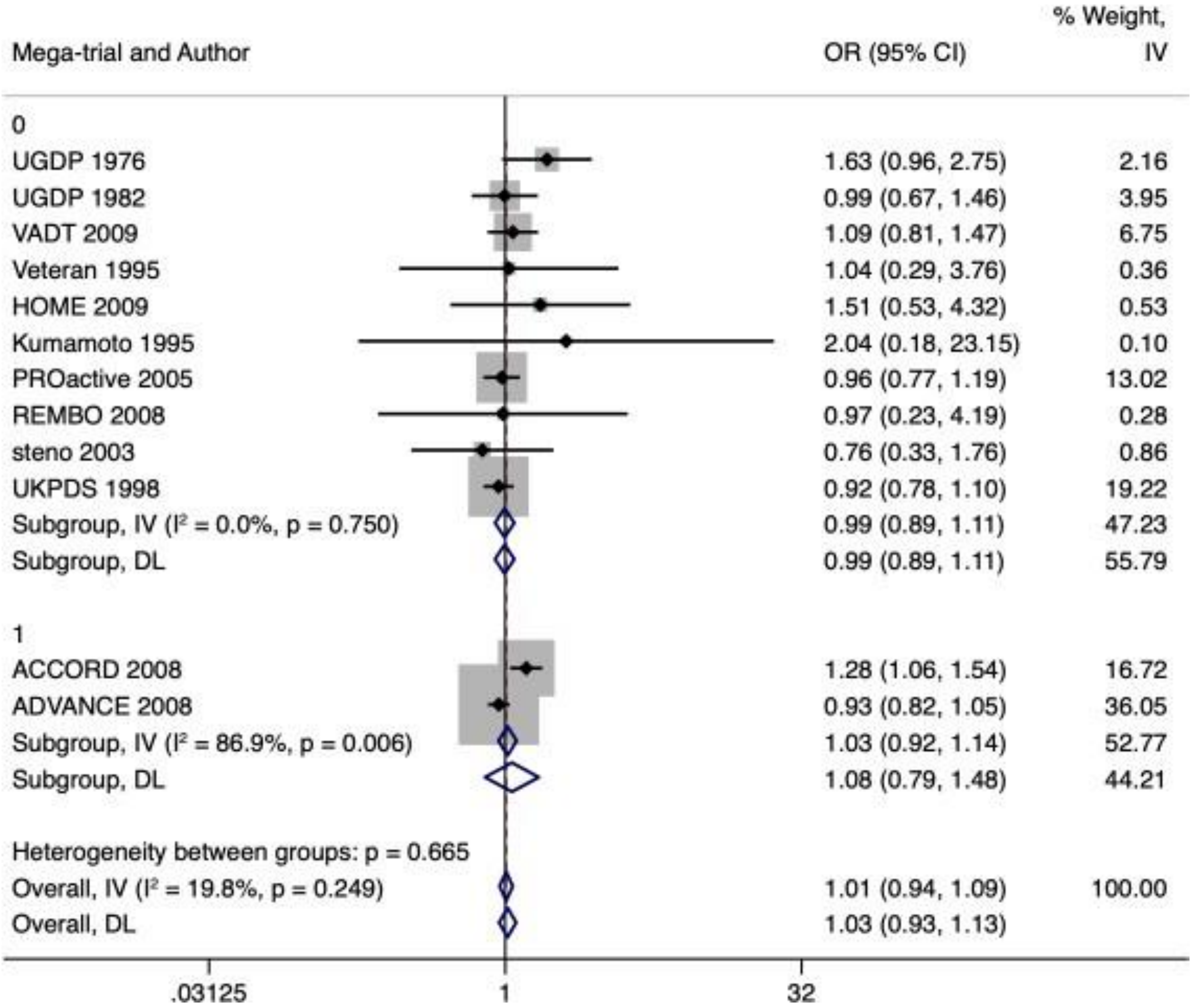

© 2024 Kastrati L et al. *JAMA Network Open.*

8. VITAL 2019

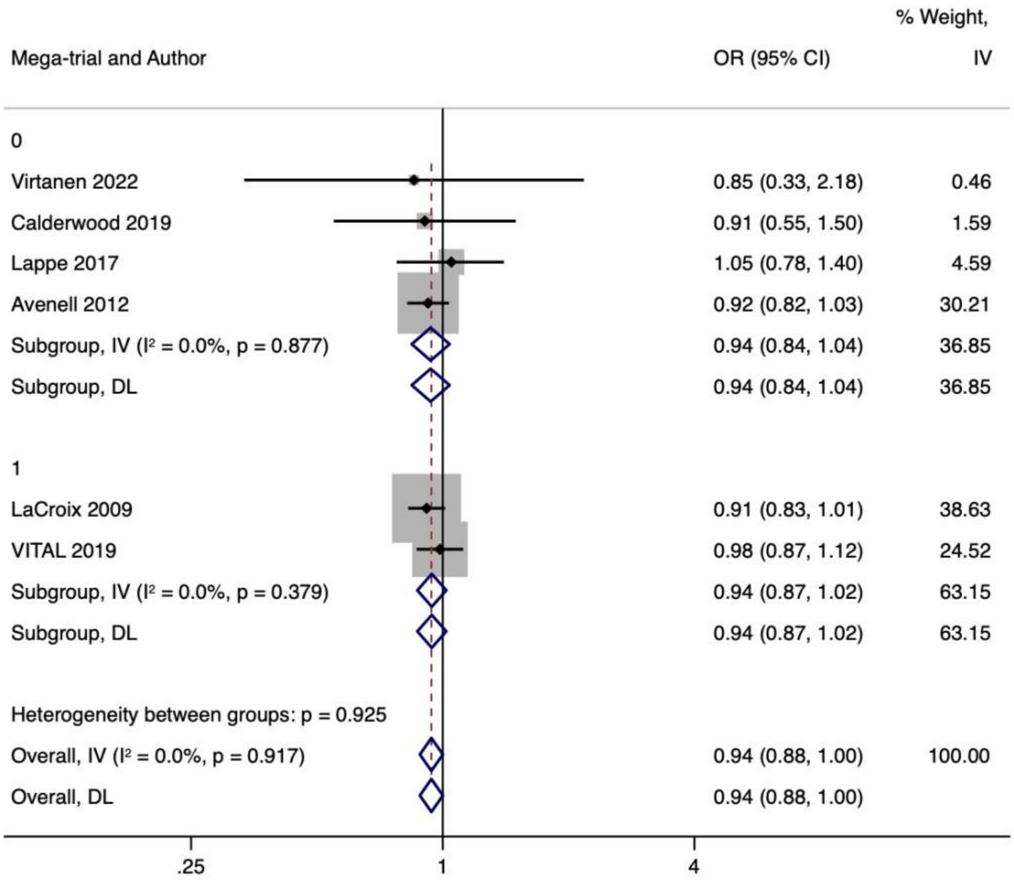

© 2024 Kastrati L et al. *JAMA Network Open.*

9. REVEAL 2017

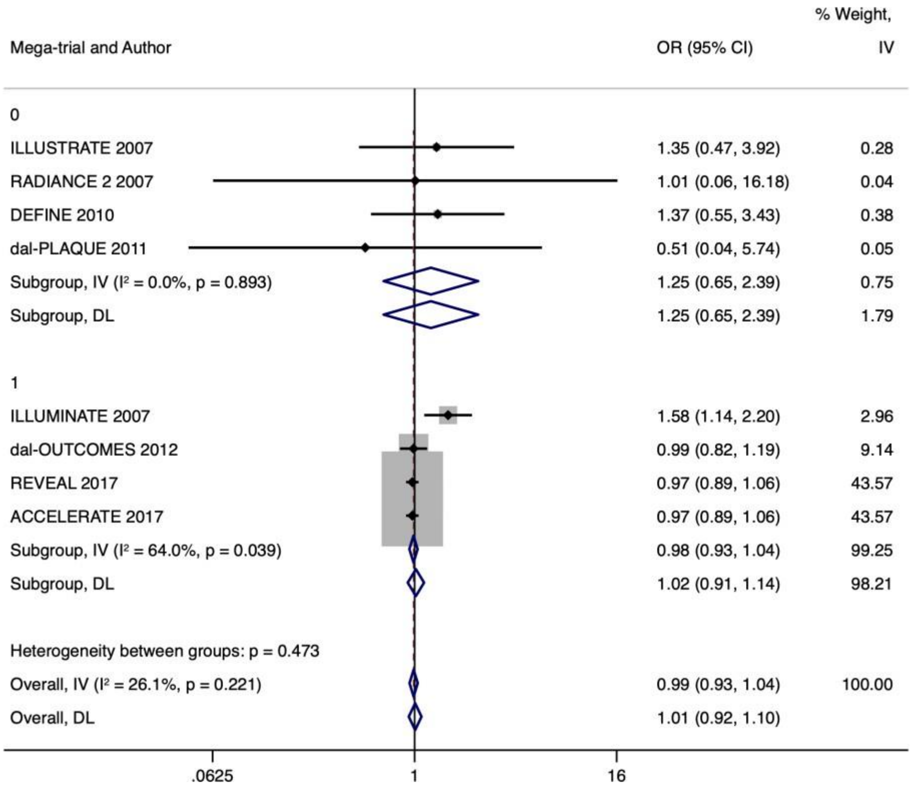

© 2024 Kastrati L et al. *JAMA Network Open.*

10. NISSEN 2016

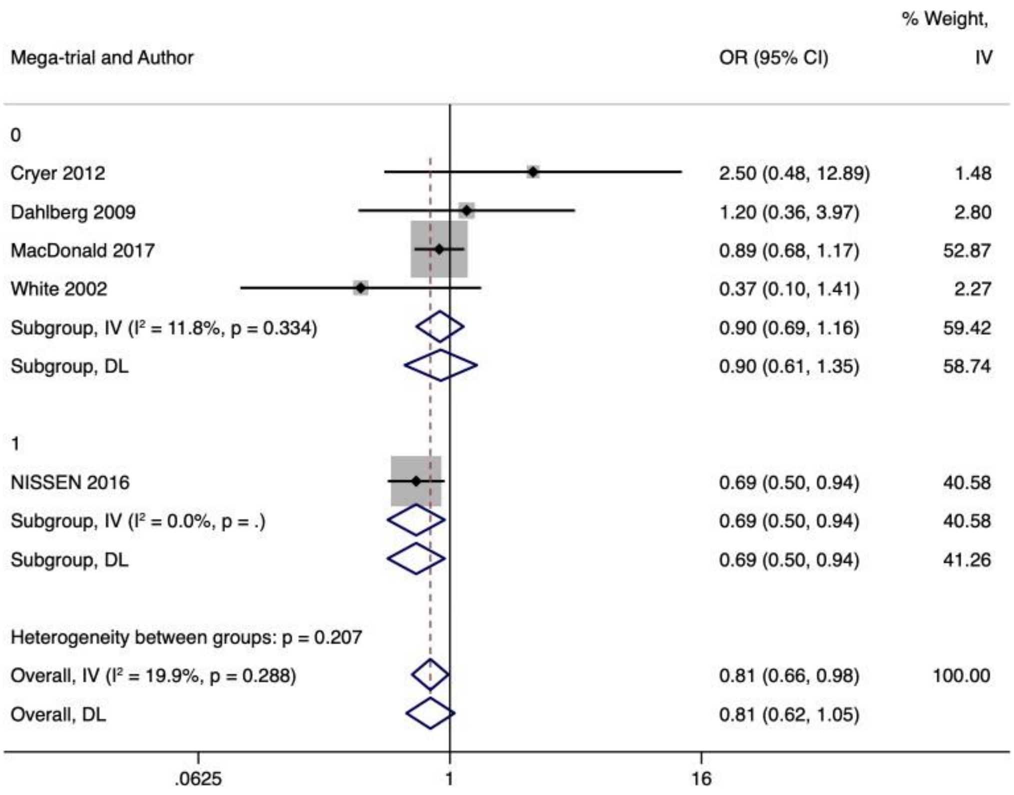

© 2024 Kastrati L et al. *JAMA Network Open.*

11. EXTRACT TIMI 25 2006

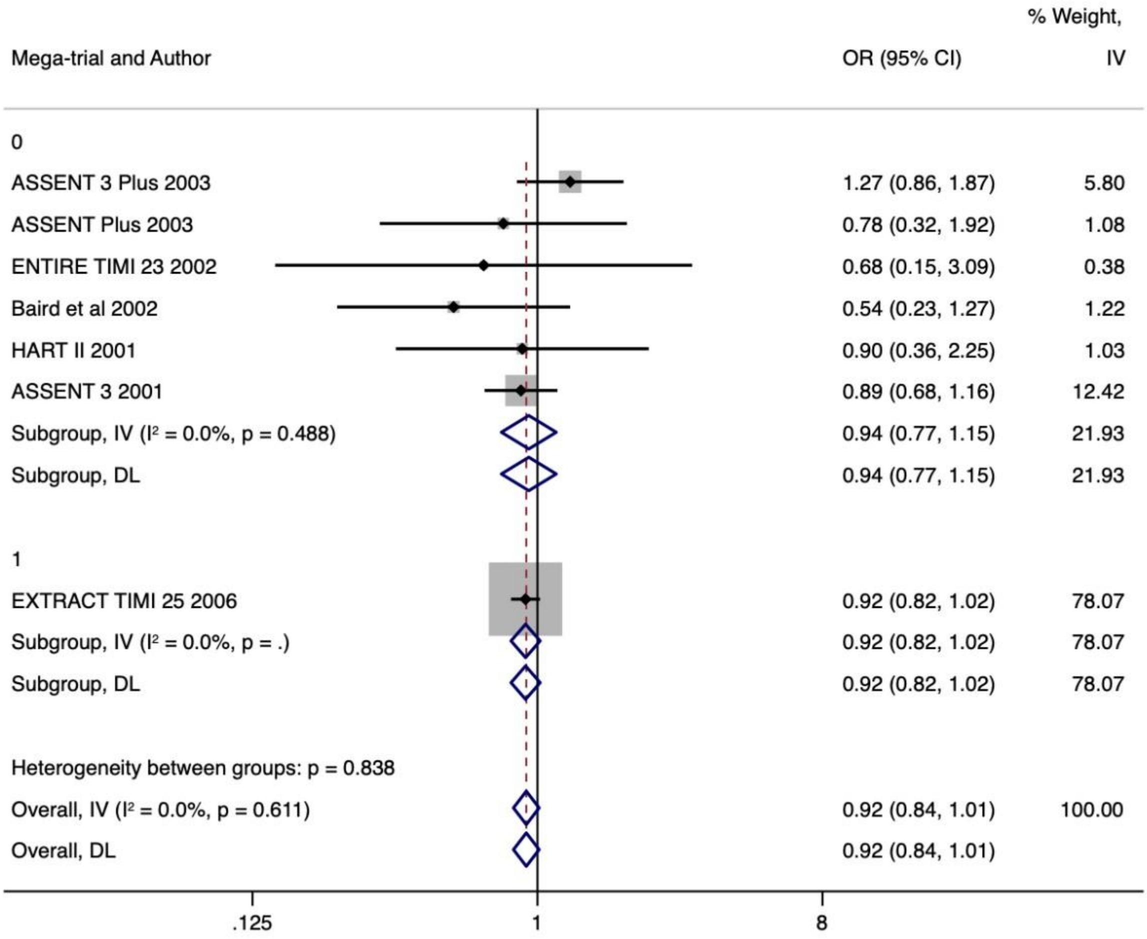

© 2024 Kastrati L et al. *JAMA Network Open.*

ODYSSEY OUTCOMES

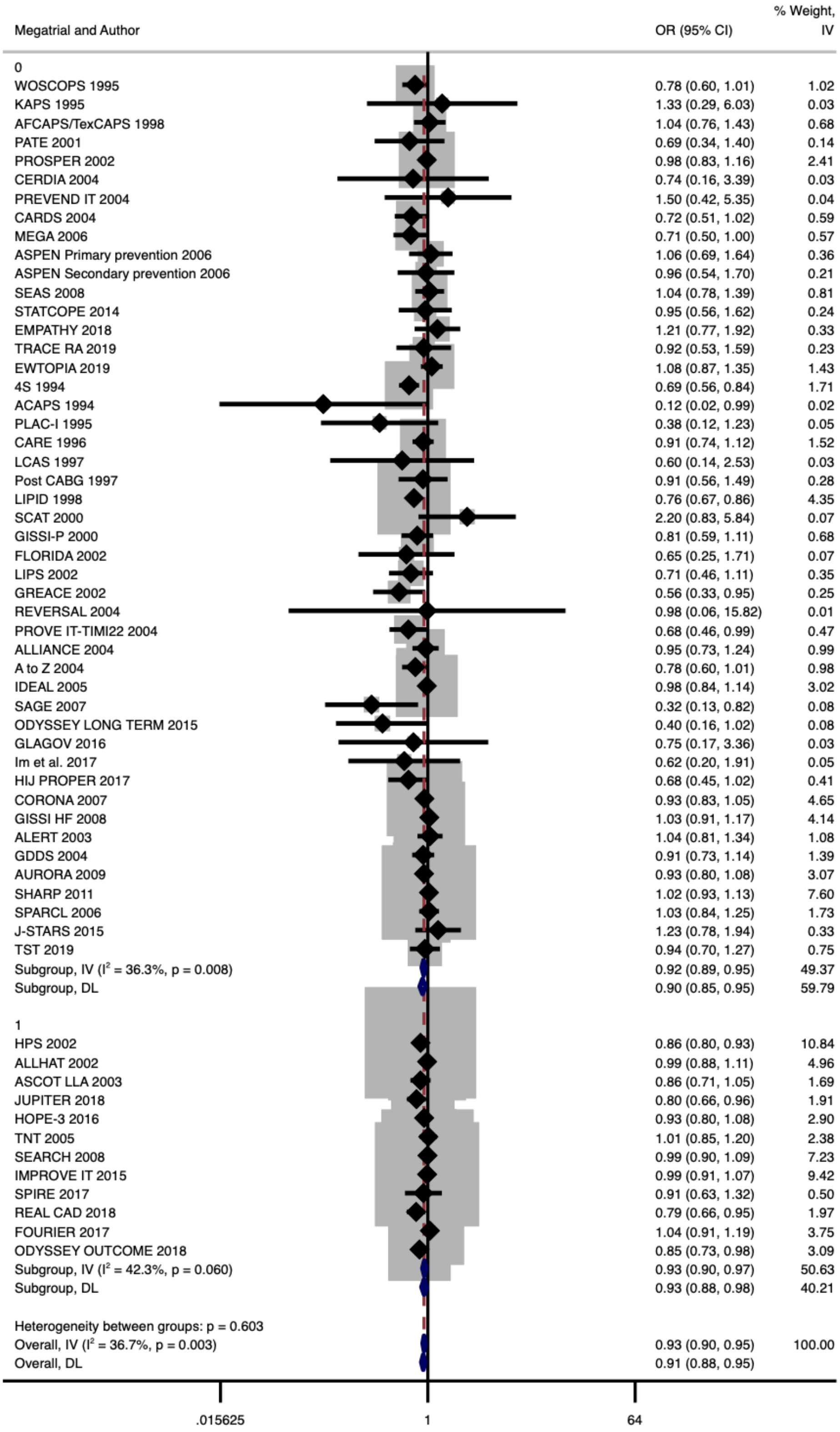

13. COMMIT 2005-

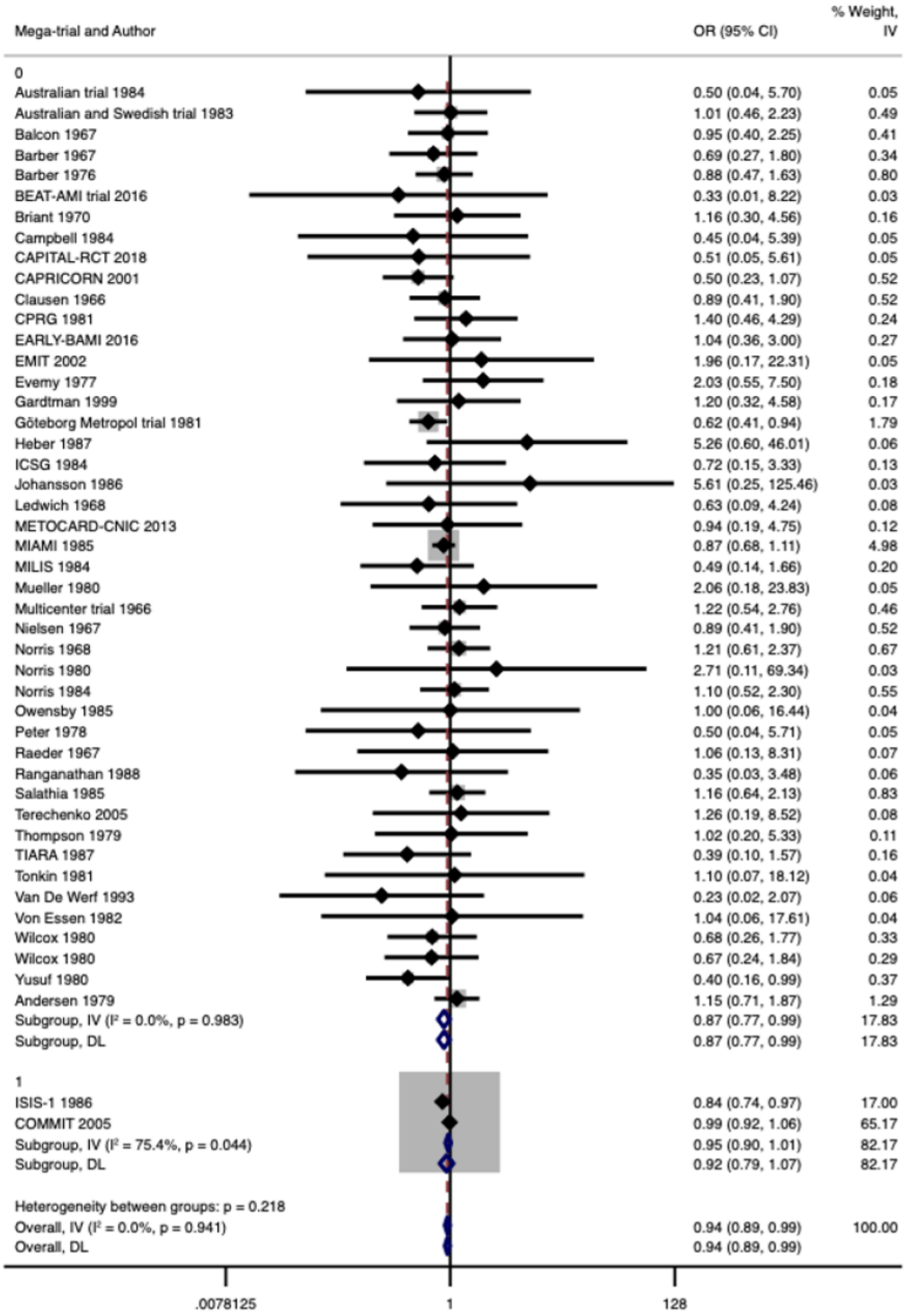

14. SIGNIFY 2014

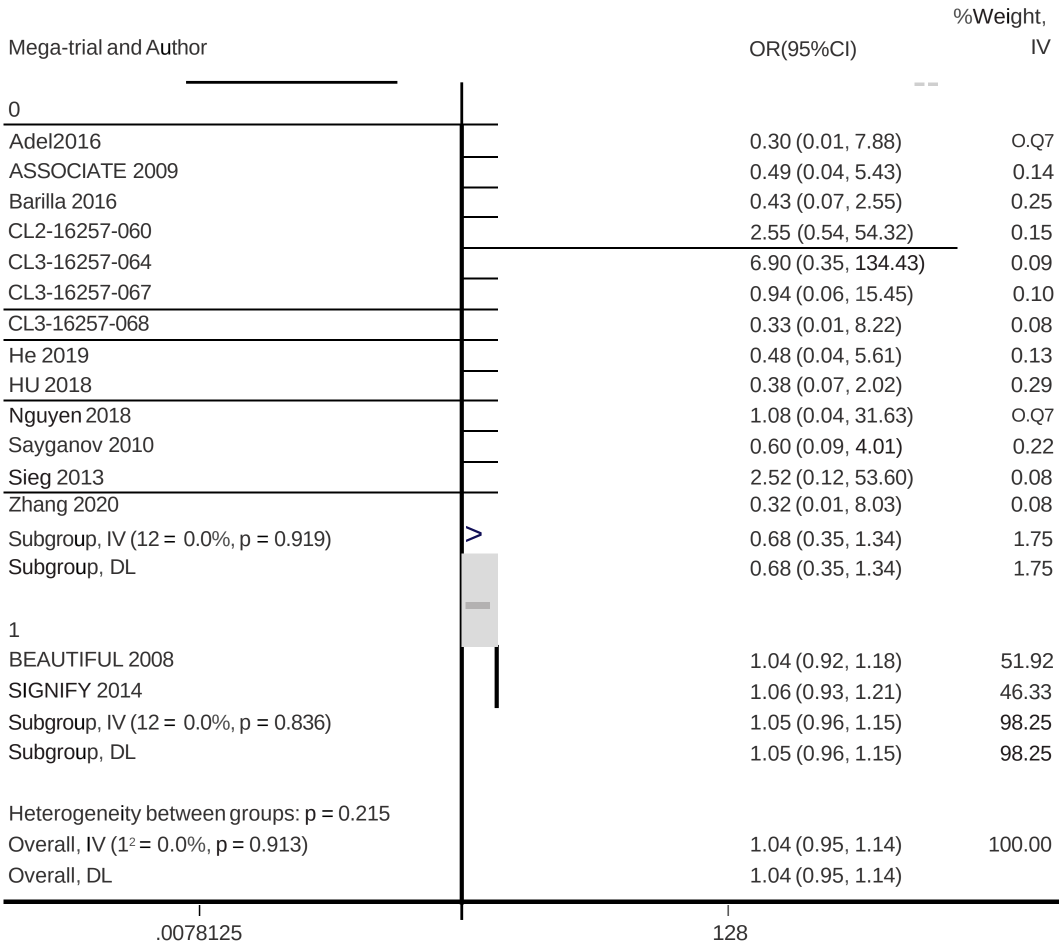

© 2024 Kastrati L et al. *JAMA Network Open.*

15. CHARISMA 2006

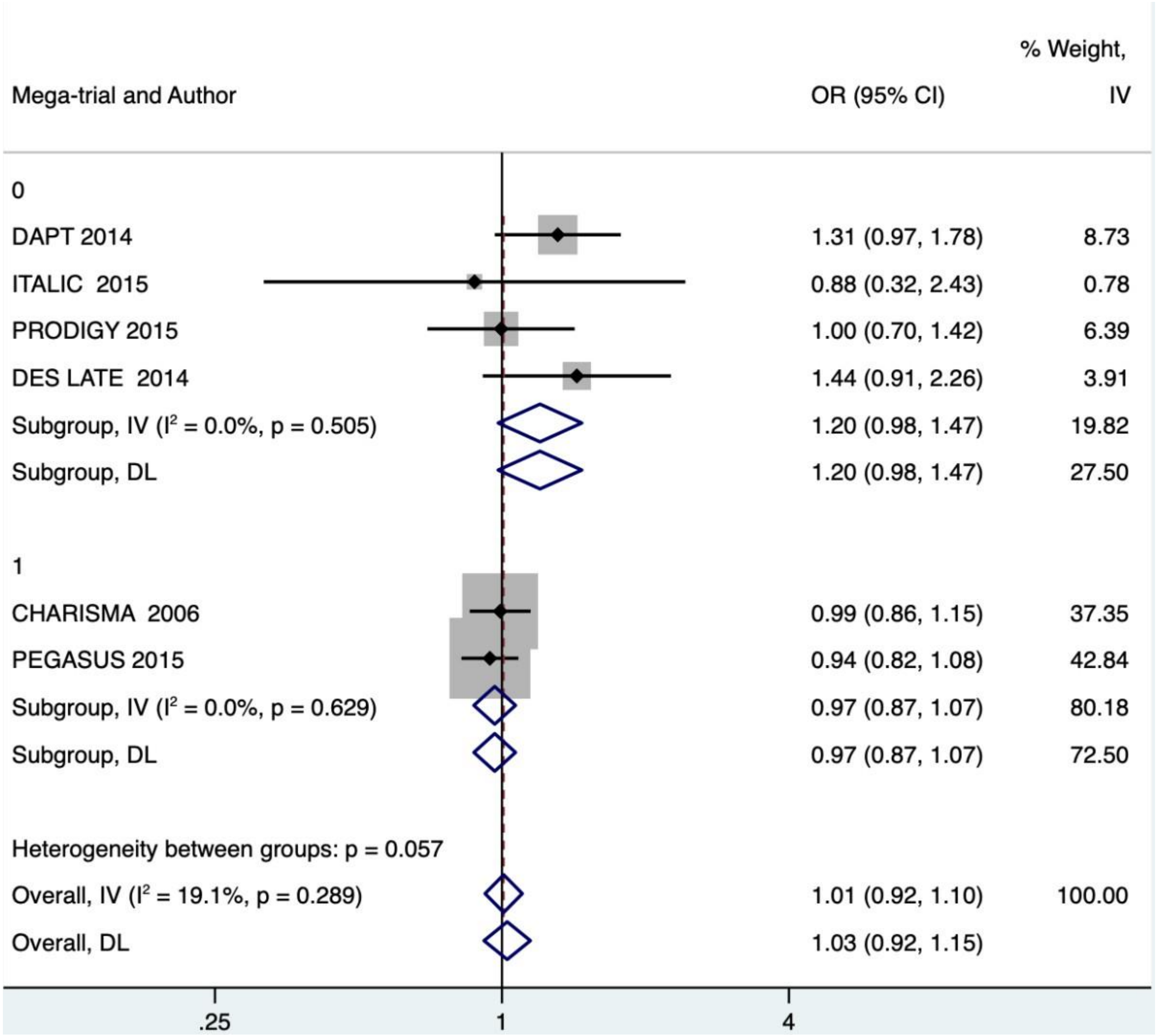

© 2024 Kastrati L et al. *JAMA Network Open.*

16. STABILITY

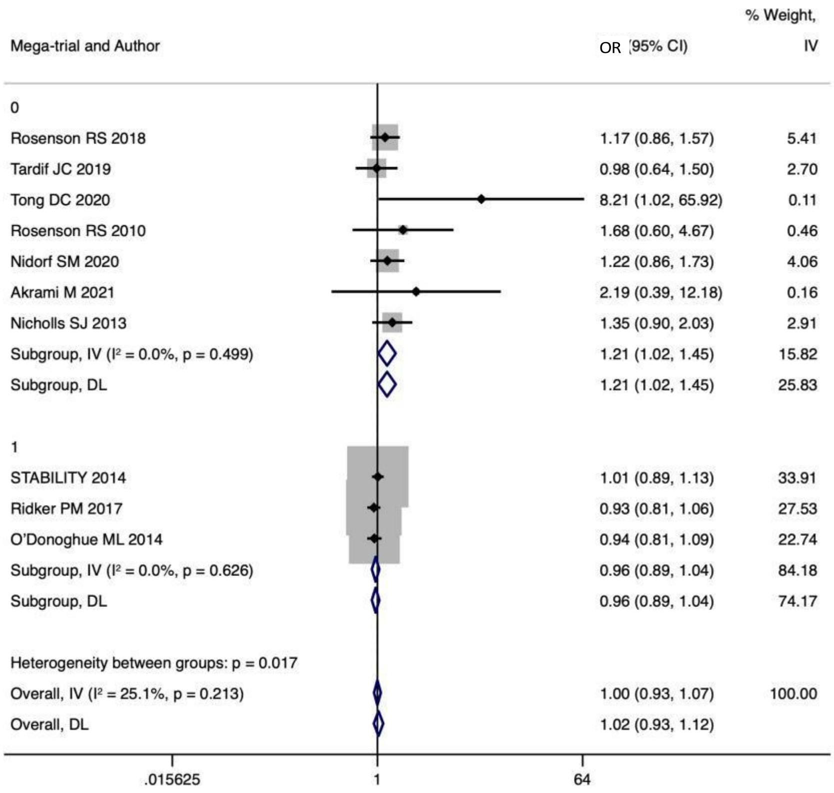

\

© 2024 Kastrati L et al. *JAMA Network Open.*

17. POISE-2 2014

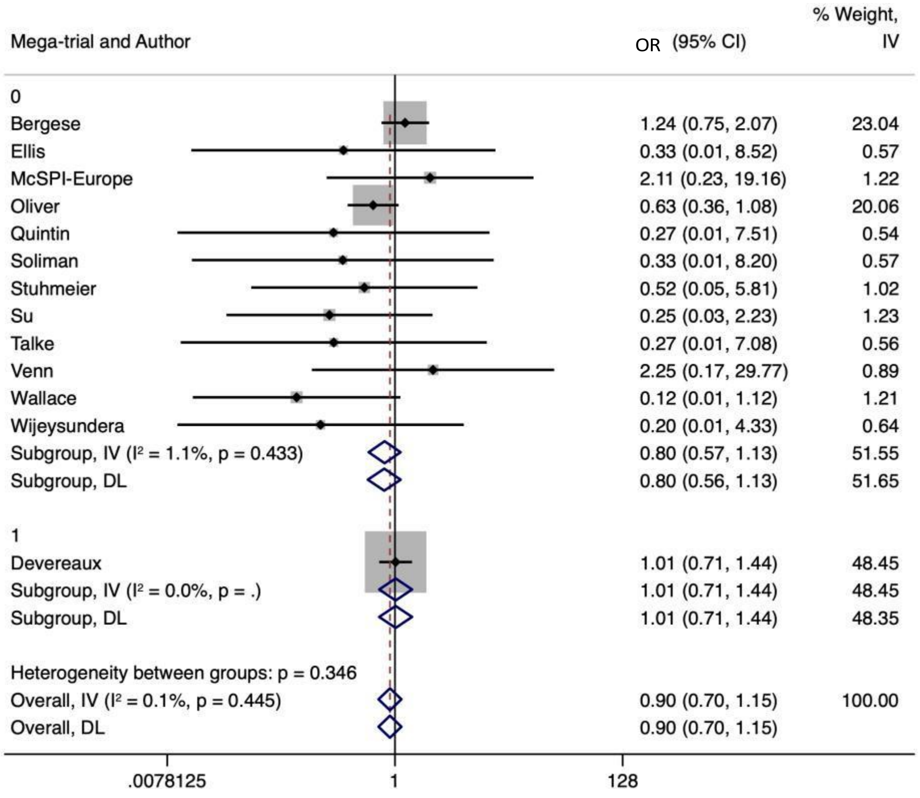

© 2024 Kastrati L et al. *JAMA Network Open.*

18. CURRENT OASIS 7- 2010

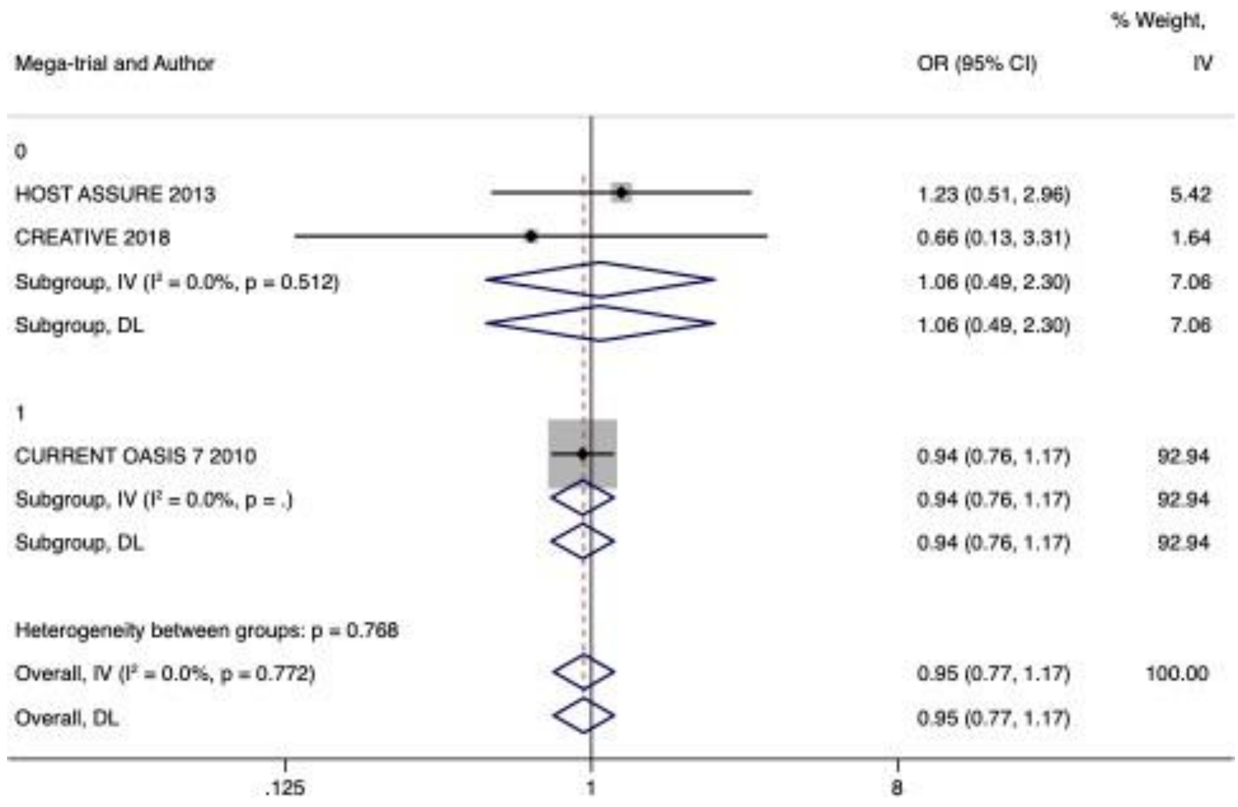

© 2024 Kastrati L et al. *JAMA Network Open.*

19. ONTARGET 2008

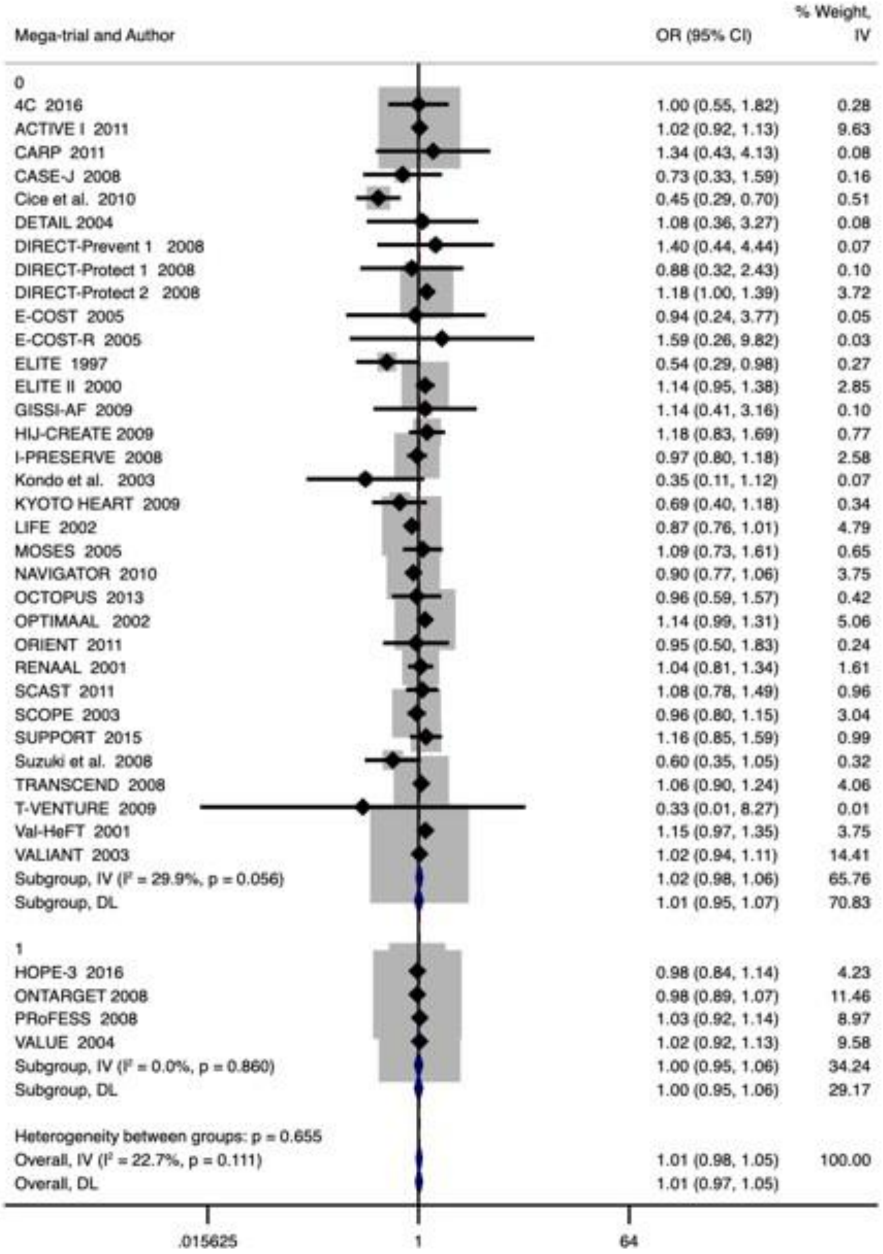

© 2024 Kastrati L et al. *JAMA Network Open.*

20. SU.VI.MAX 2004

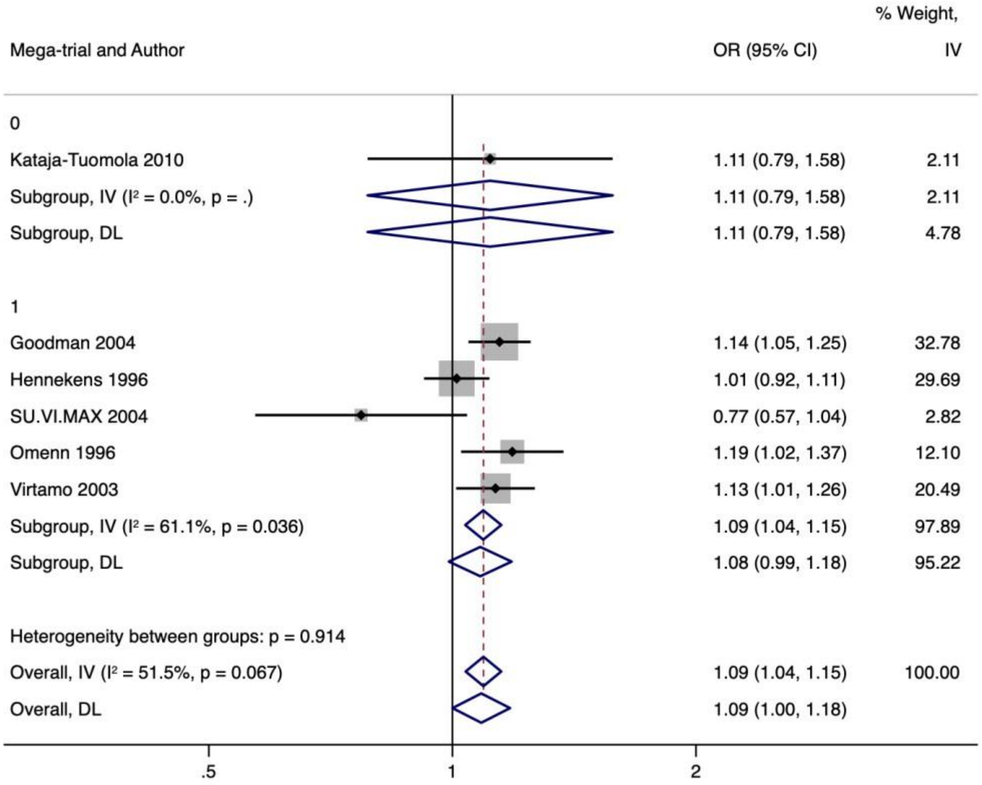

© 2024 Kastrati L et al. *JAMA Network Open.*

21. ATLAS 2012

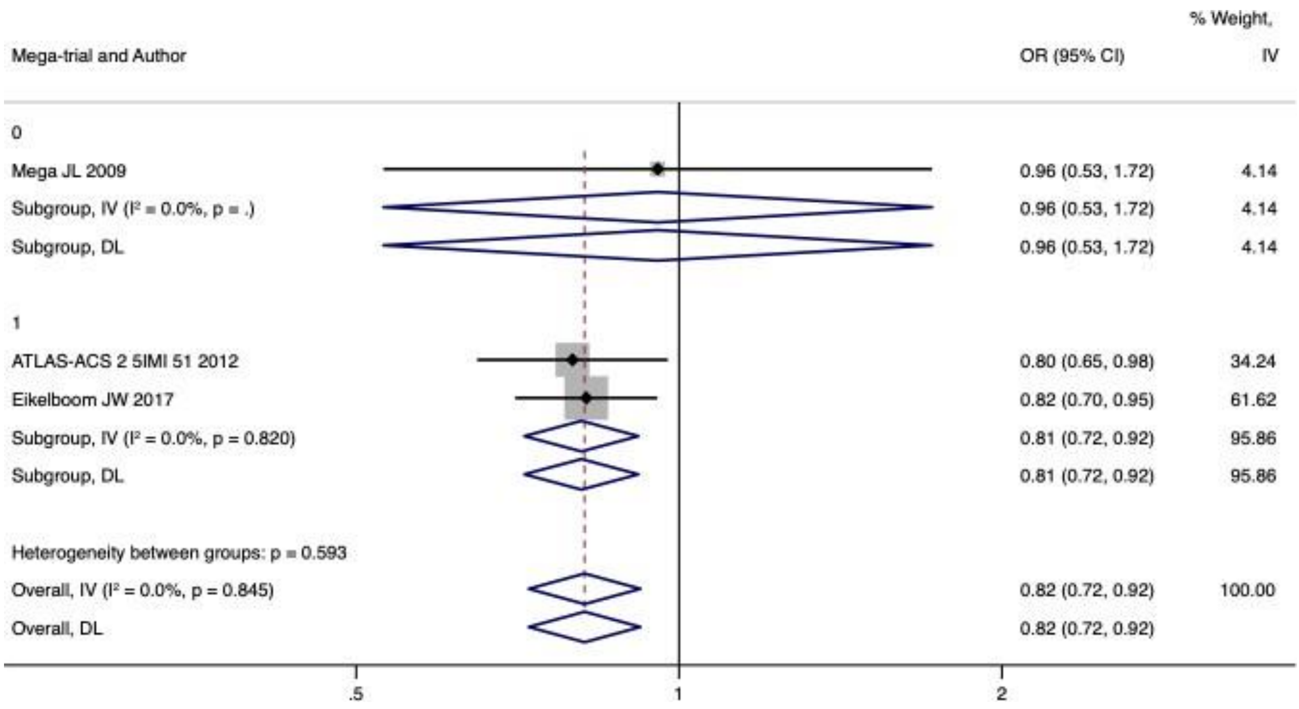

© 2024 Kastrati L et al. *JAMA Network Open.*

22. GLOBAL LEADERS 2018

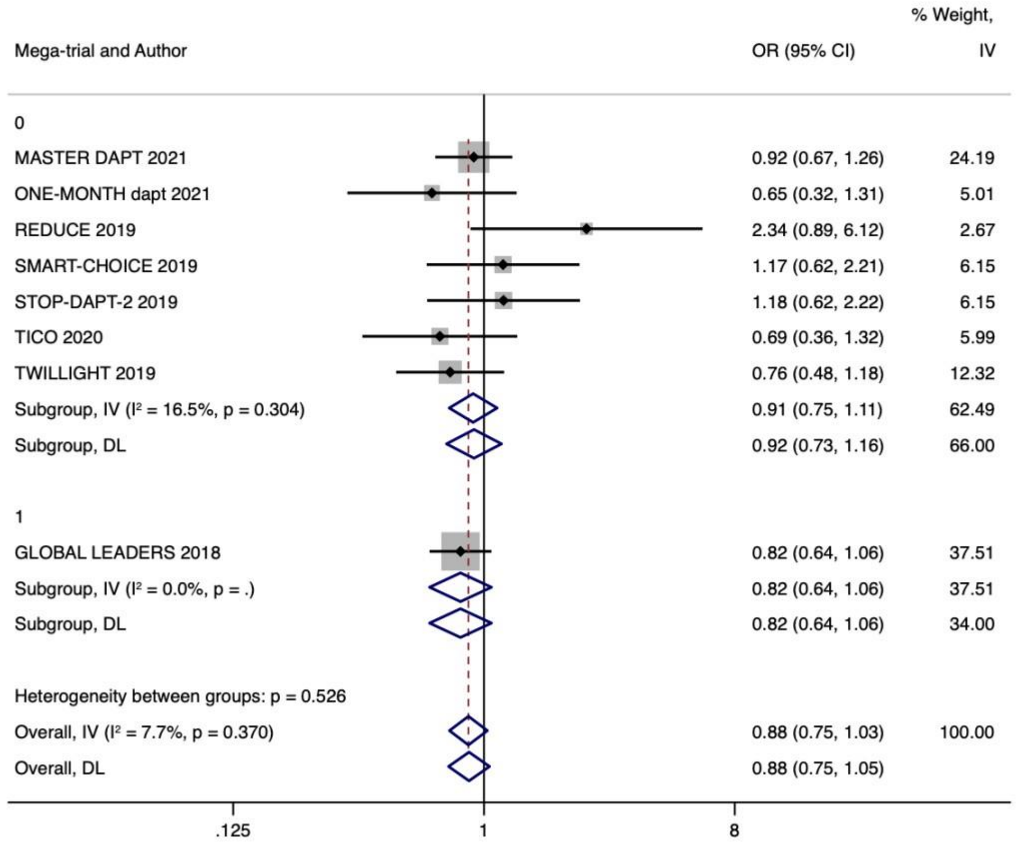

© 2024 Kastrati L et al. *JAMA Network Open.*

23 WALLENTIN – There is no Forest plot for this study as there was only one megatrial and one smaller trial.

© 2024 Kastrati L et al. *JAMA Network Open.*

24 SAVOR TIMI-53 2013

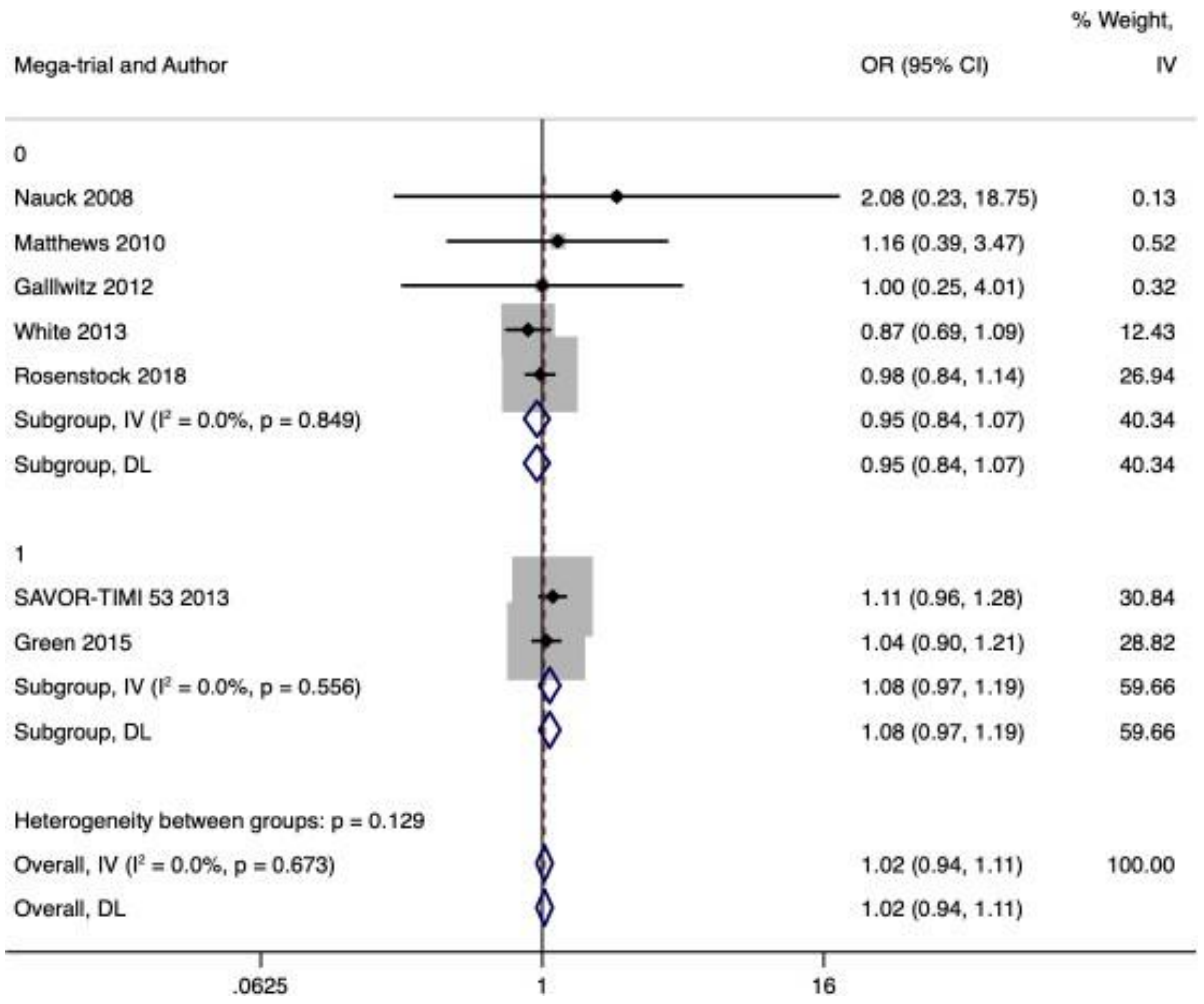

© 2024 Kastrati L et al. *JAMA Network Open.*

25 SCORED 2021

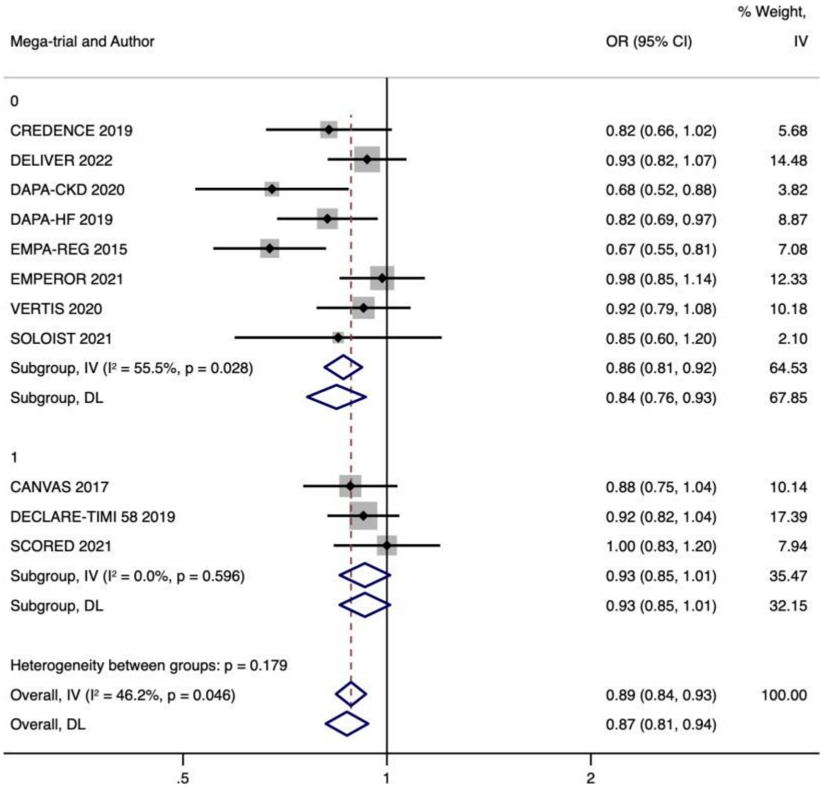

© 2024 Kastrati L et al. *JAMA Network Open.*

26. EXSCEL 2017

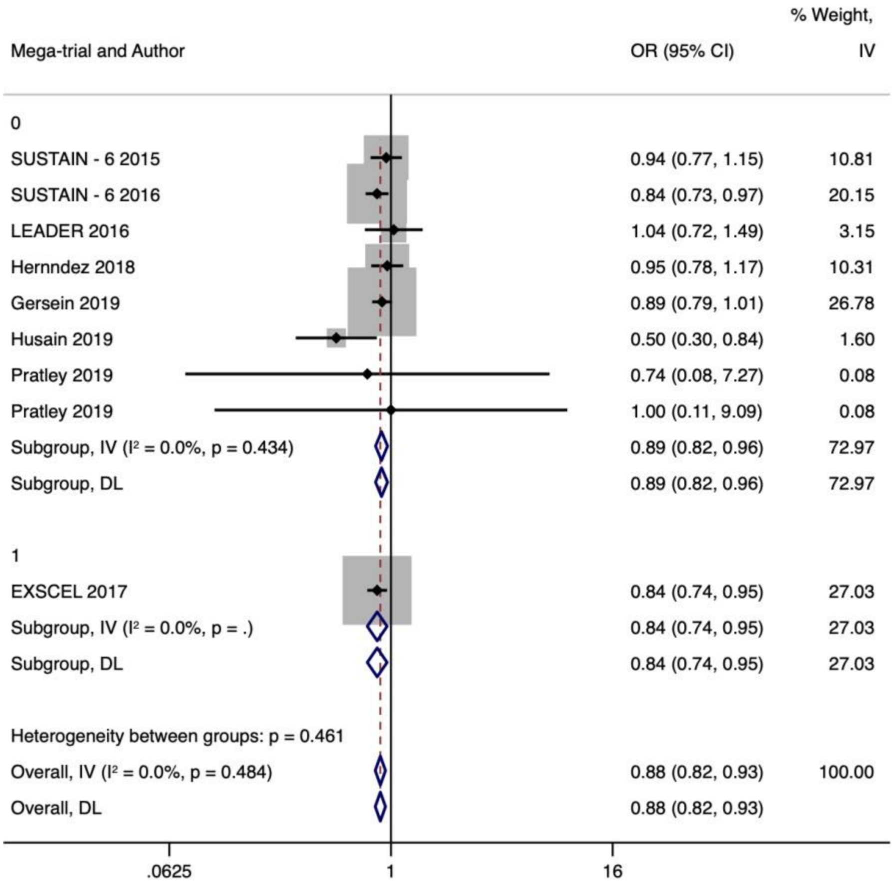

© 2024 Kastrati L et al. *JAMA Network Open.*

eFigure 2. Agreement Between Mega-Trials and Smaller Trials for All-Cause Mortality: Random Effects (DerSimonian Laird)

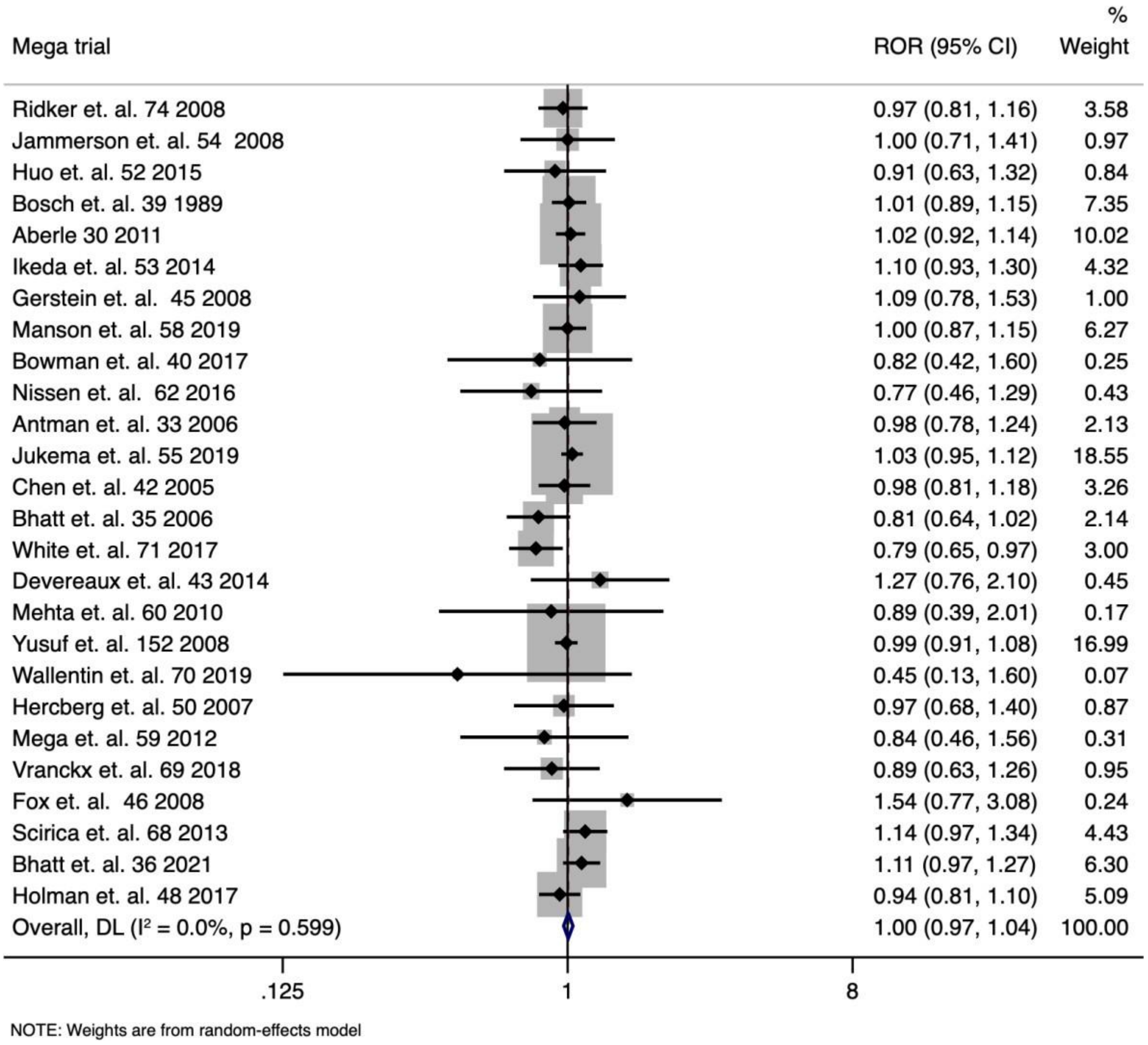

© 2024 Kastrati L et al. *JAMA Network Open.*

eFigure 3. Agreement Between Smaller Trials Prior and After the Publication of the First Mega-Trial

3A. Primary Outcome

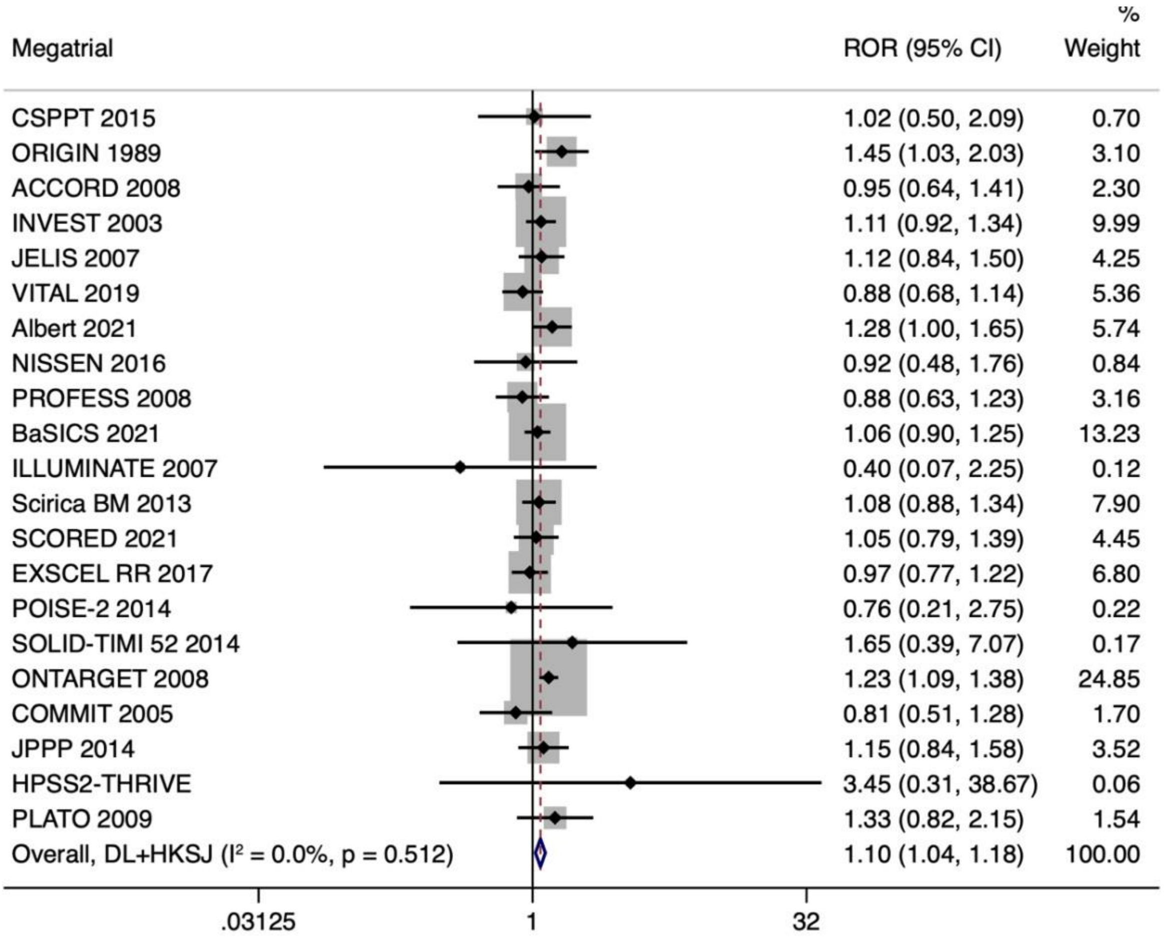

NOTE: Weights are from random-effects model

© 2024 Kastrati L et al. *JAMA Network Open.*

3B. All-Cause Mortality

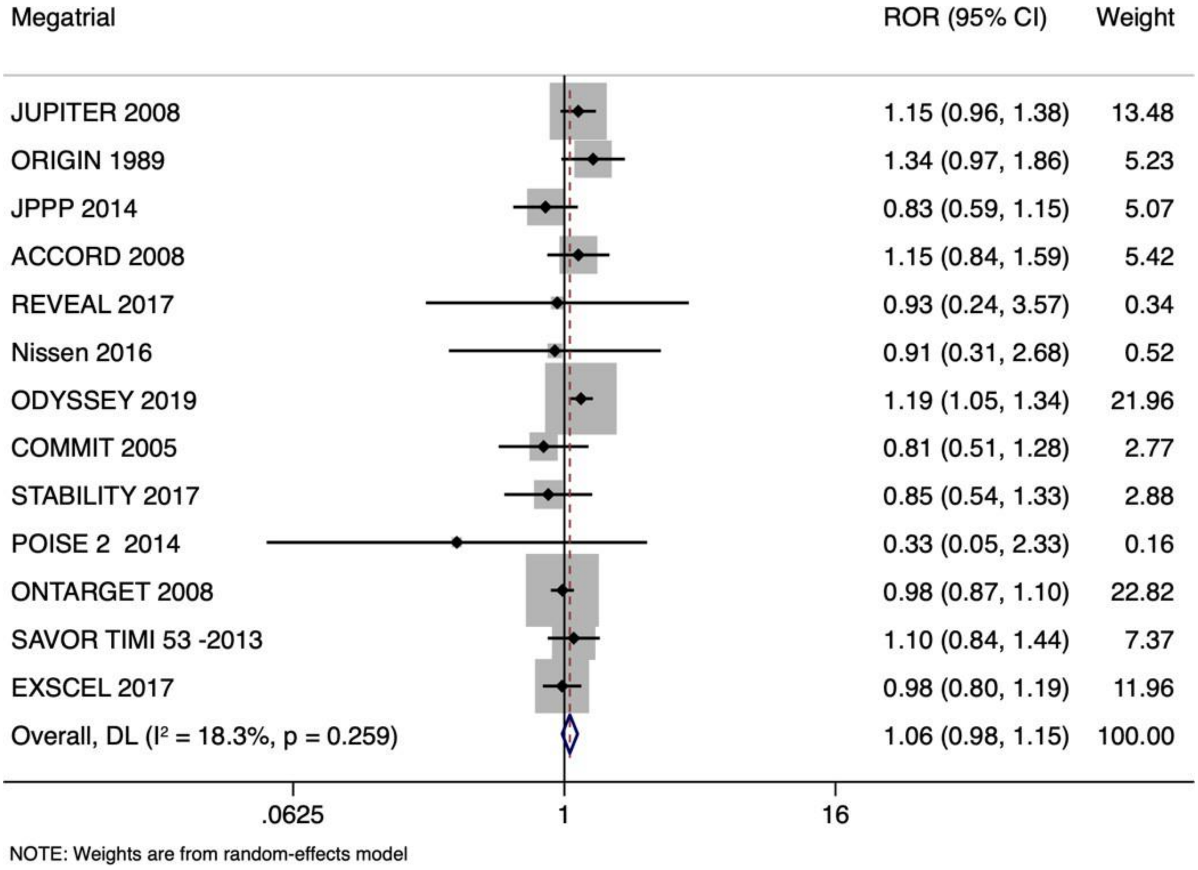

© 2024 Kastrati L et al. *JAMA Network Open.*

eTable 3. Results of Uni- and Multivariable Meta-Regression

	Primary Outcome		All-Cause Mortality	
	Coefficient (SE)	P-value	Coefficient (SE)	P-value
Intervention	-0.01(0.08)	0.89	.009 (0.1)	0.9
Speciality	0.01(0.05)	0.757	0.02 (0.06)	0.688
ROB-MEGA	0.01 (0.07)	0.87	0.01 (0.05)	0.76
ROB-SMALL	0.04 (0.05)	0.307	0.01 (0.05)	0.854
Median_small	-3.93e-06 (0.00001)	0.77	-1.42e (0.00002)	0.95
Total-small	1.28e-06 (1.74e-06)	0.46	1.44e-07 (7.03e-07)	0.838
Multivariate analyses		Non-significant		Non-significant

SE- Standard Error
ROB-MEGA- Proportion of mega-trials at high risk of bias.
ROB-SMALL- Proportion of smaller trials at high risk of bias.
Median-small- The median number of participants on the smaller trials.
Total-small- The total number of participants in the smaller trials.
Multivariate Analysis- Meta regression including all of the above listed variables.

© 2024 Kastrati L et al. *JAMA Network Open.*

eFigure 4. Agreement Between Mega-Trials and Smaller Trials With 1/5 of the Least Weighted Mega-Trial

4A. Primary Outcome

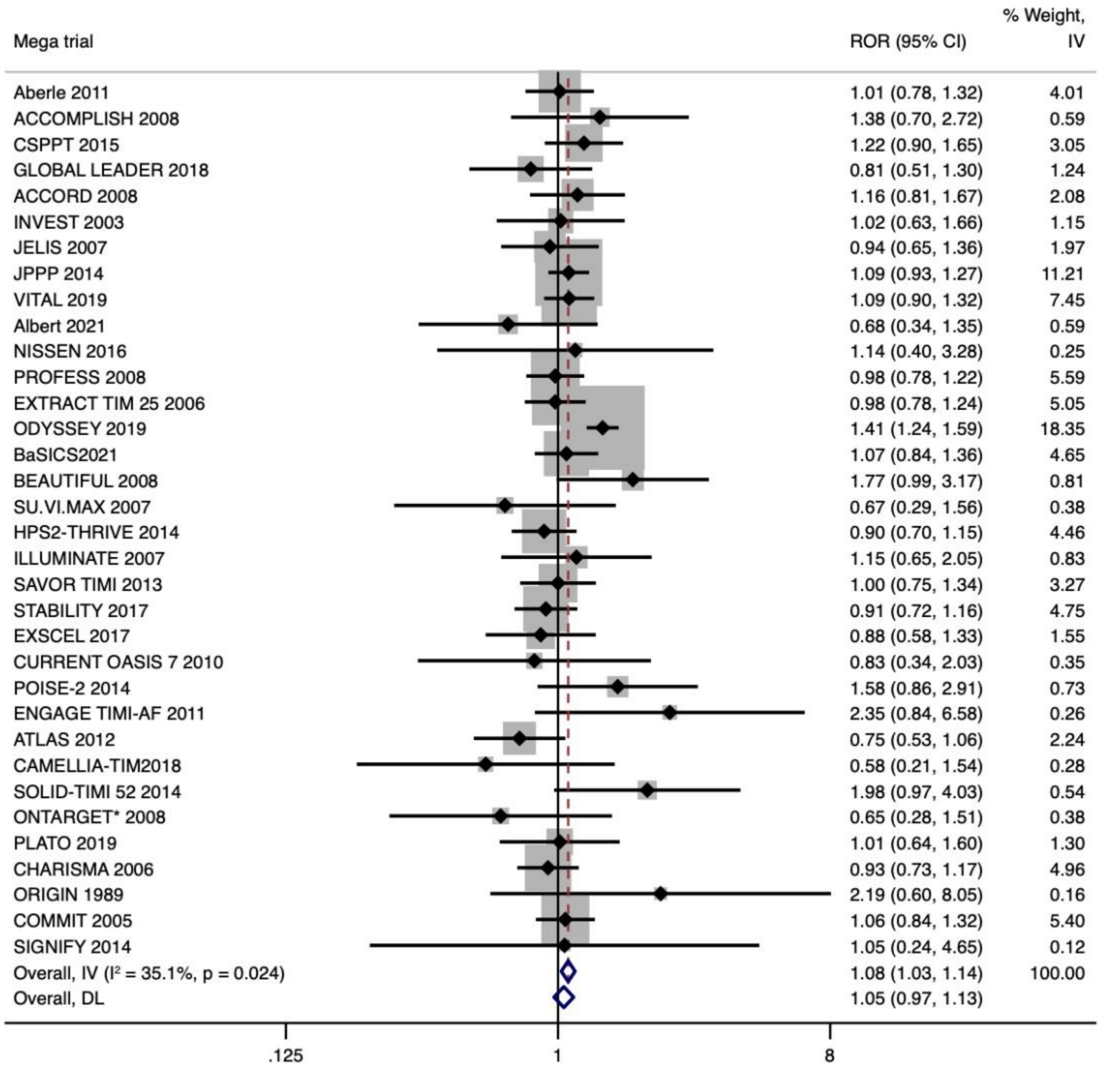

© 2024 Kastrati L et al. *JAMA Network Open.*

4B. Primary Outcome

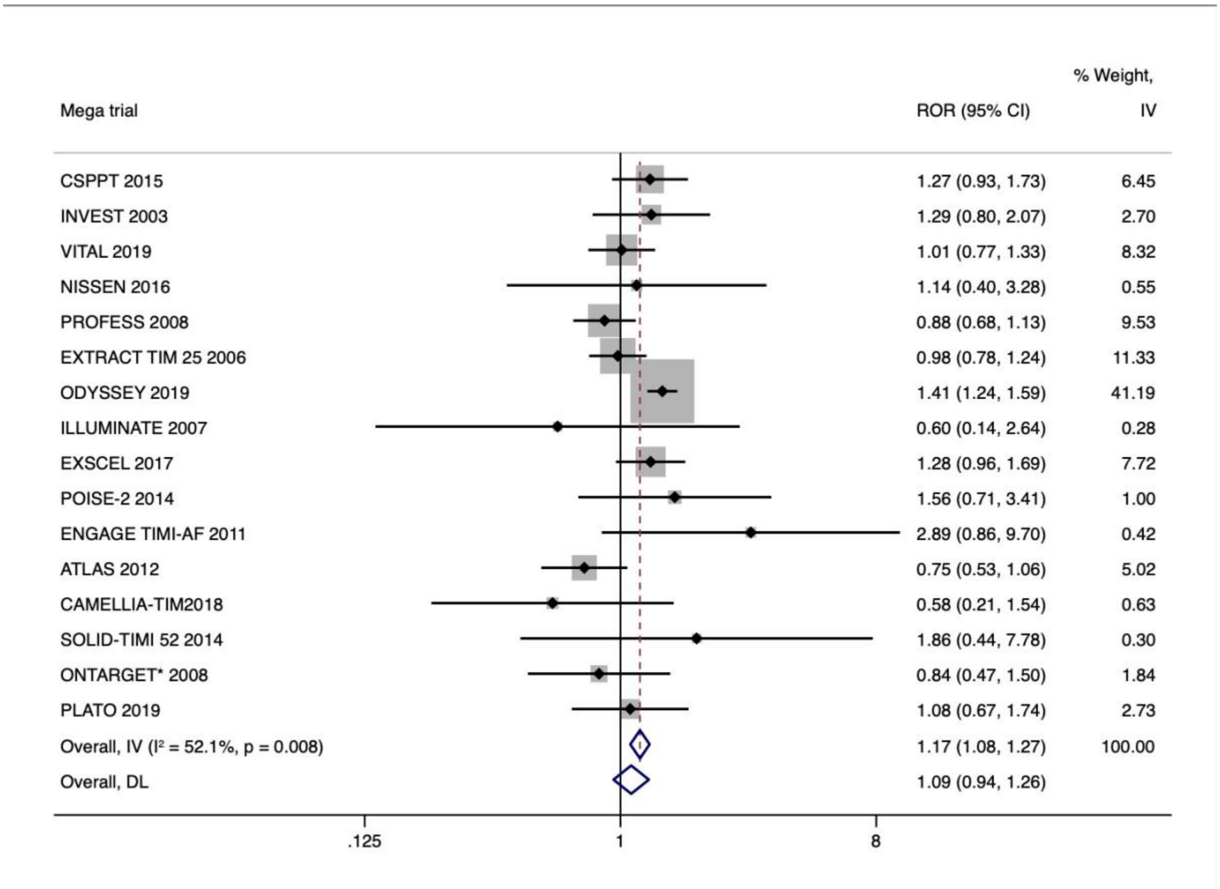

© 2024 Kastrati L et al. *JAMA Network Open.*

4C. All-Cause Mortality

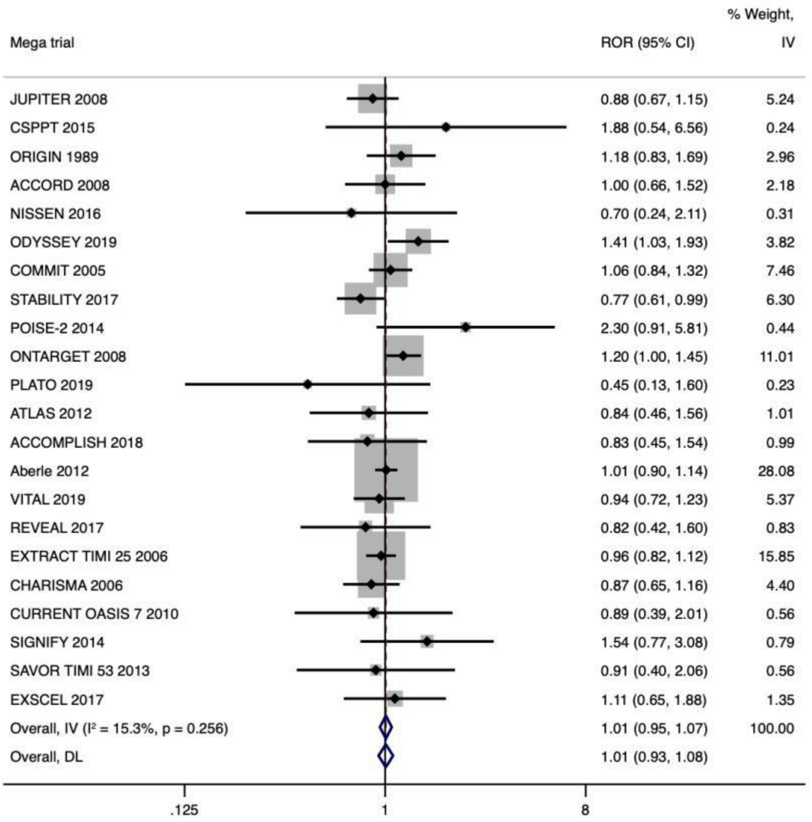

© 2024 Kastrati L et al. *JAMA Network Open.*

4D. Agreement Between Mega-Trials and Smaller Trials Trials With 1/5 of the Least Weighted Mega-Trial Published Prior or Up Until The Mega-Trial: All-Cause Mortality

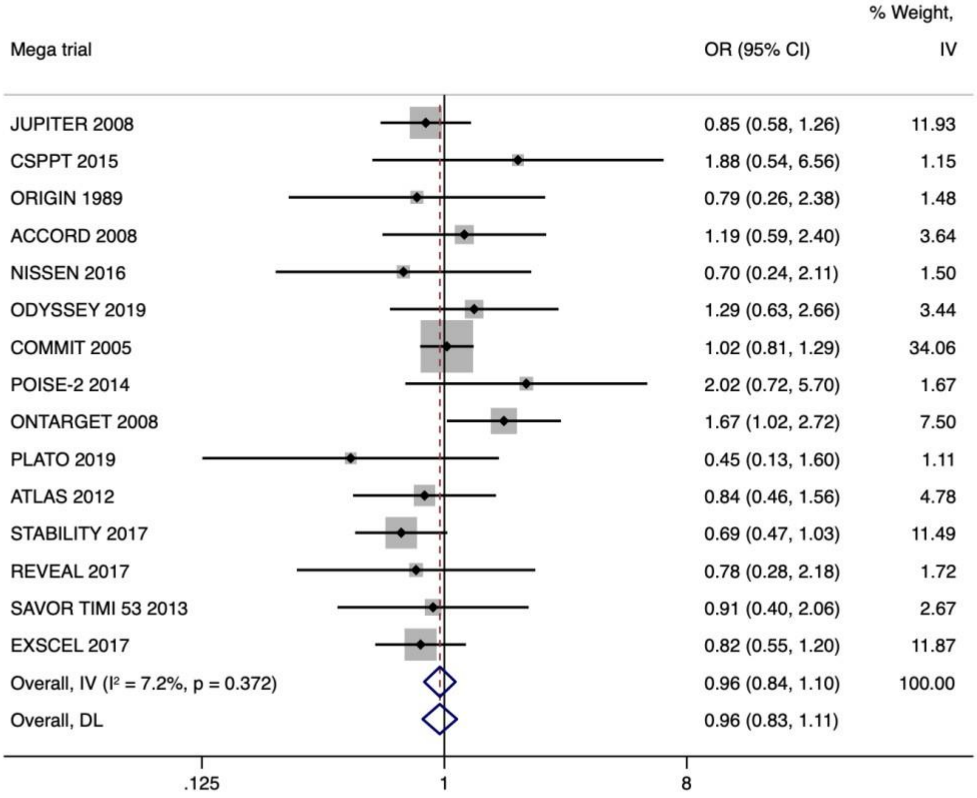

© 2024 Kastrati L et al. *JAMA Network Open.*

eFigure 5. Agreement Between Mega-Trials and Smaller Trials With 1/10 of the Least Weighted Megatrial

5A. Primary Outcome

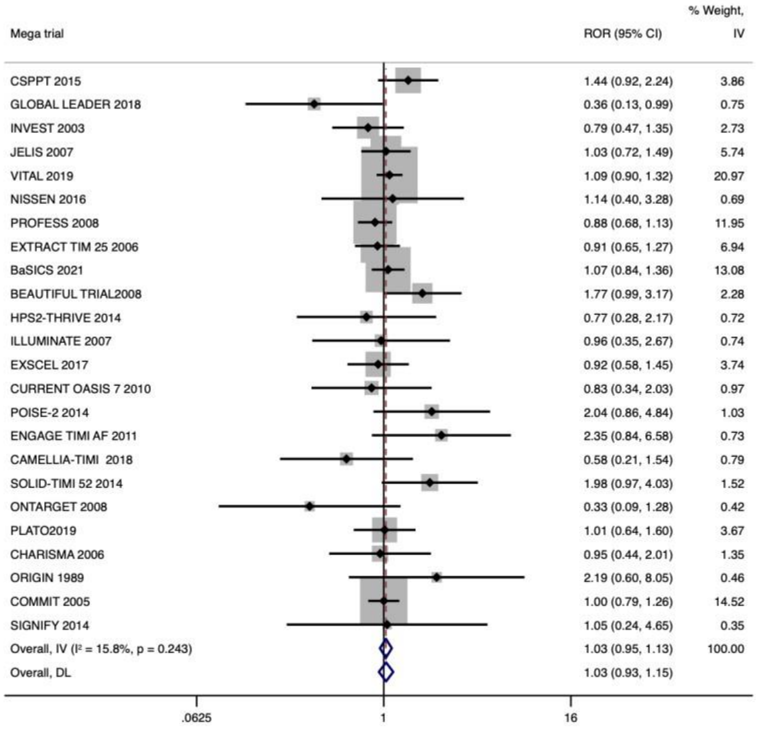

© 2024 Kastrati L et al. *JAMA Network Open.*

5B. Agreement Between Mega-Trials and Smaller Trials With 1/10 of the Least Weighted Mega-Trial Published Prior or Up Until the Mega-Trial – Primary Outcome

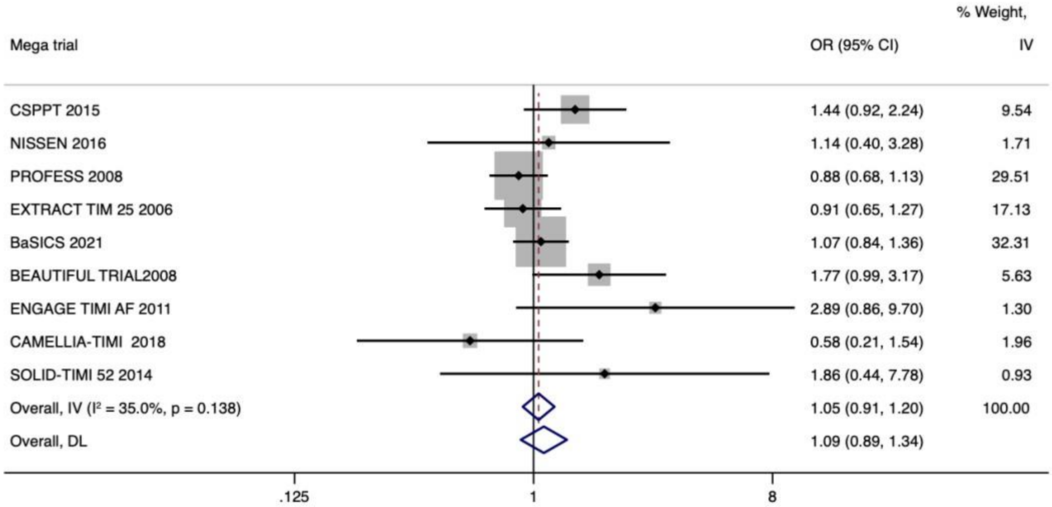

© 2024 Kastrati L et al. *JAMA Network Open.*

5C. Agreement Between Mega-Trials and Smaller Trials With 1/10 of the Least Weighted Megatrial – All-Cause Mortality

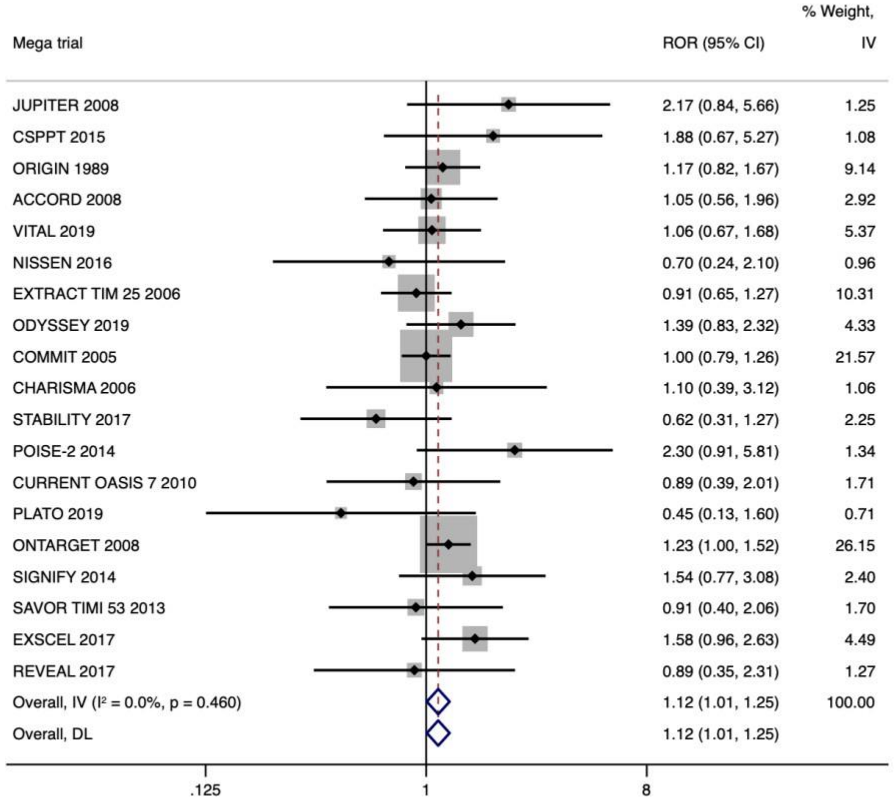

© 2024 Kastrati L et al. *JAMA Network Open.*

5D. Agreement Between Mega-Trials and Smaller Trials With 1/10 of the Least Weighted Mega-Trial Published Prior or Up Until the Mega-Trial – Primary Outcome

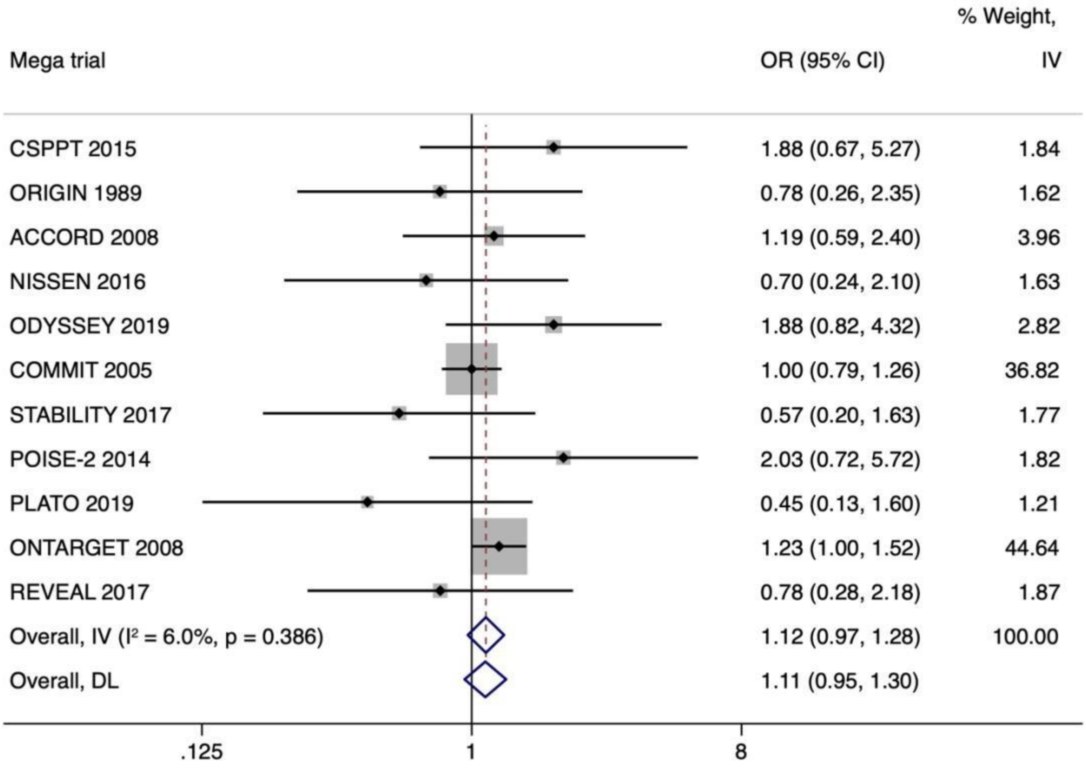

© 2024 Kastrati L et al. *JAMA Network Open.*

eFigure 6. Agreement Between Mega-Trials and Smaller Trials, Pooling the Results Using Fixed Effects

6A. Primary Outcome

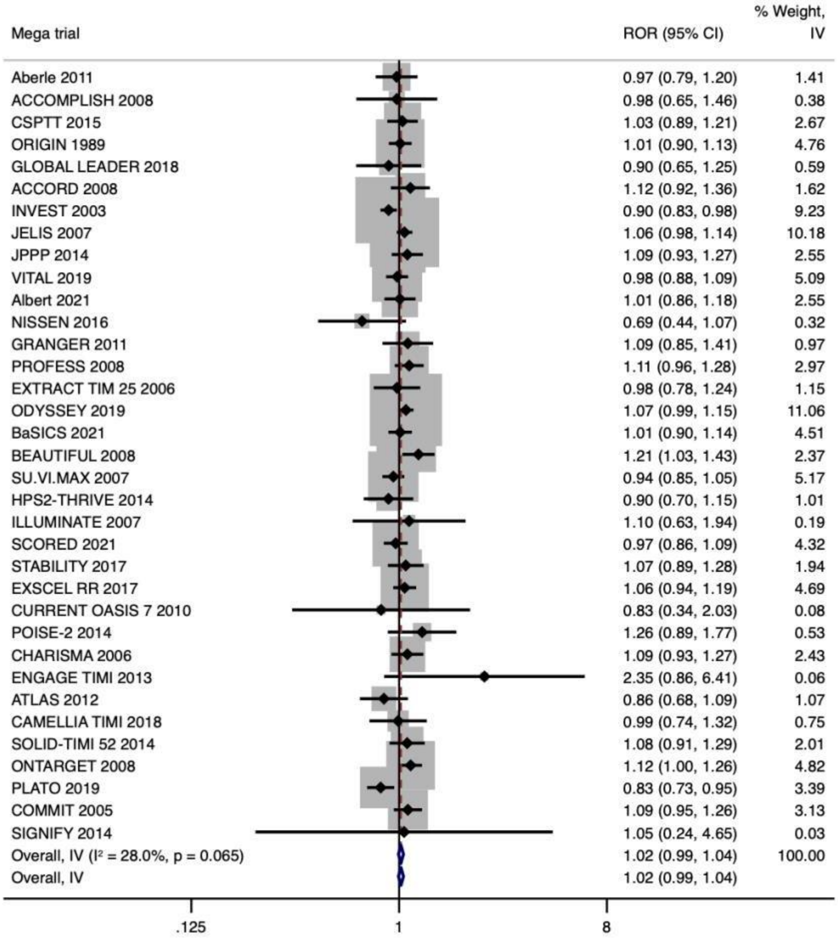

© 2024 Kastrati L et al. *JAMA Network Open.*

6B. All-Cause Mortality

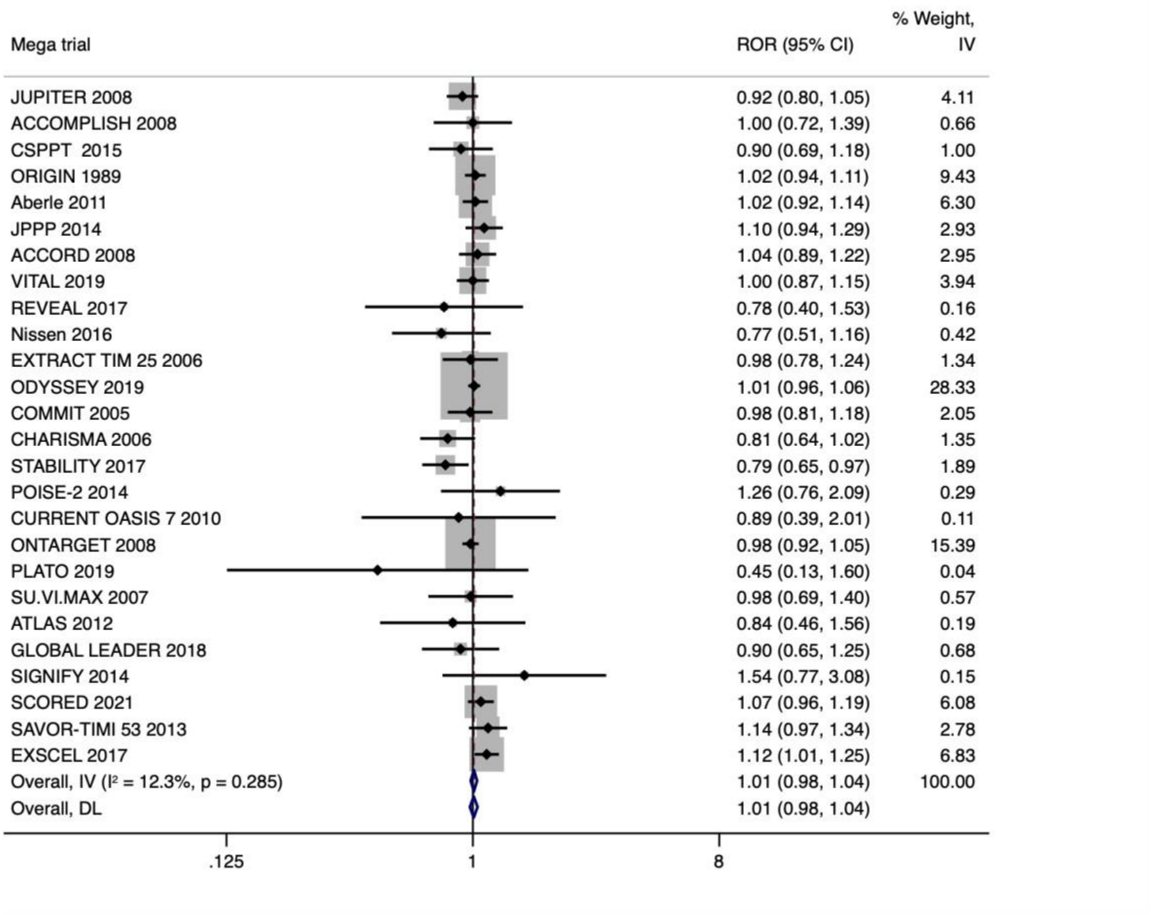

© 2024 Kastrati L et al. *JAMA Network Open.*

eFigure 7. Agreement Between Mega-Trials and Smaller Trials, Pooling the Results Using Random Effects – HKSJ Method

7A. Primary Outcome

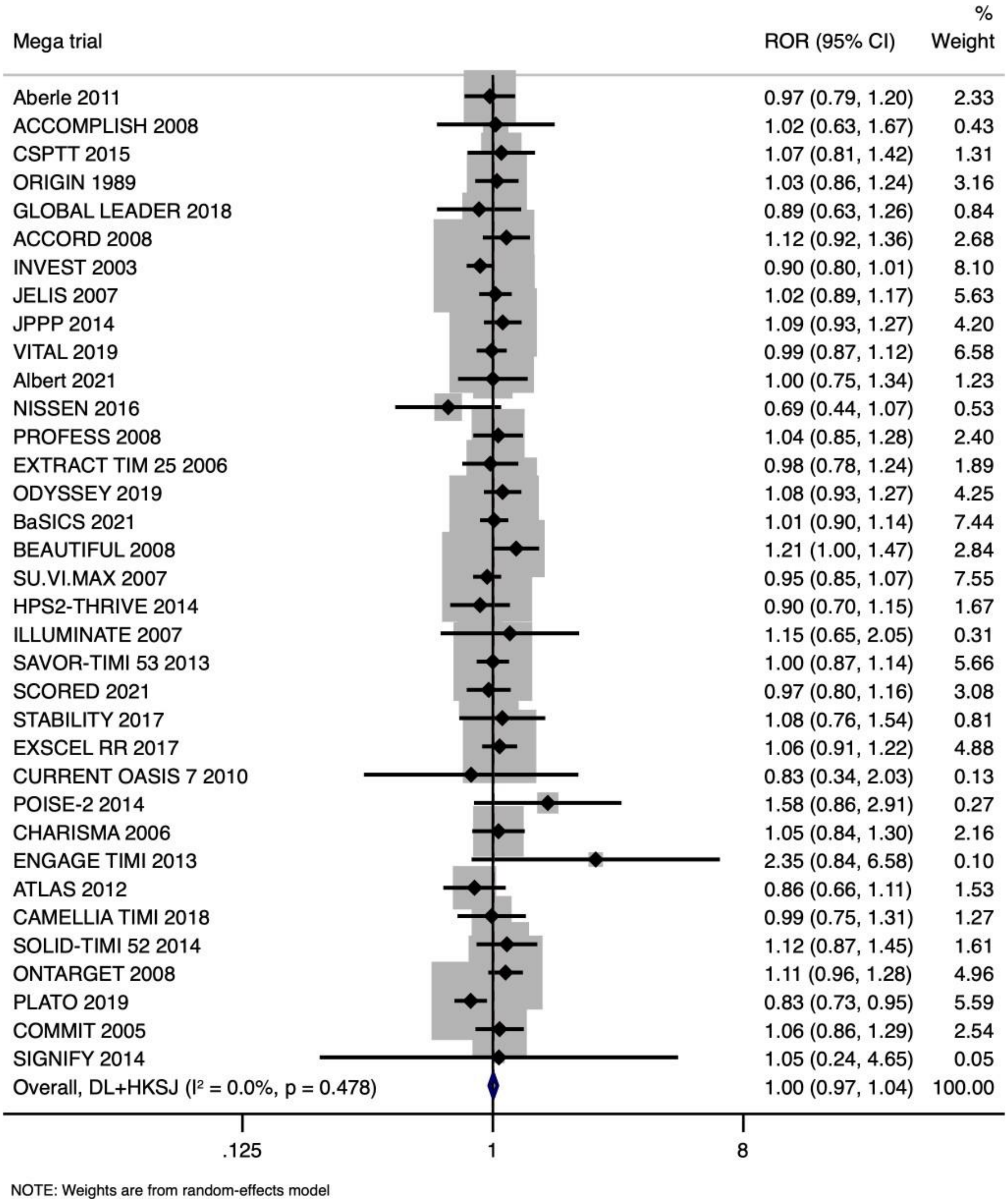

© 2024 Kastrati L et al. *JAMA Network Open.*

7B. All-Cause Mortality

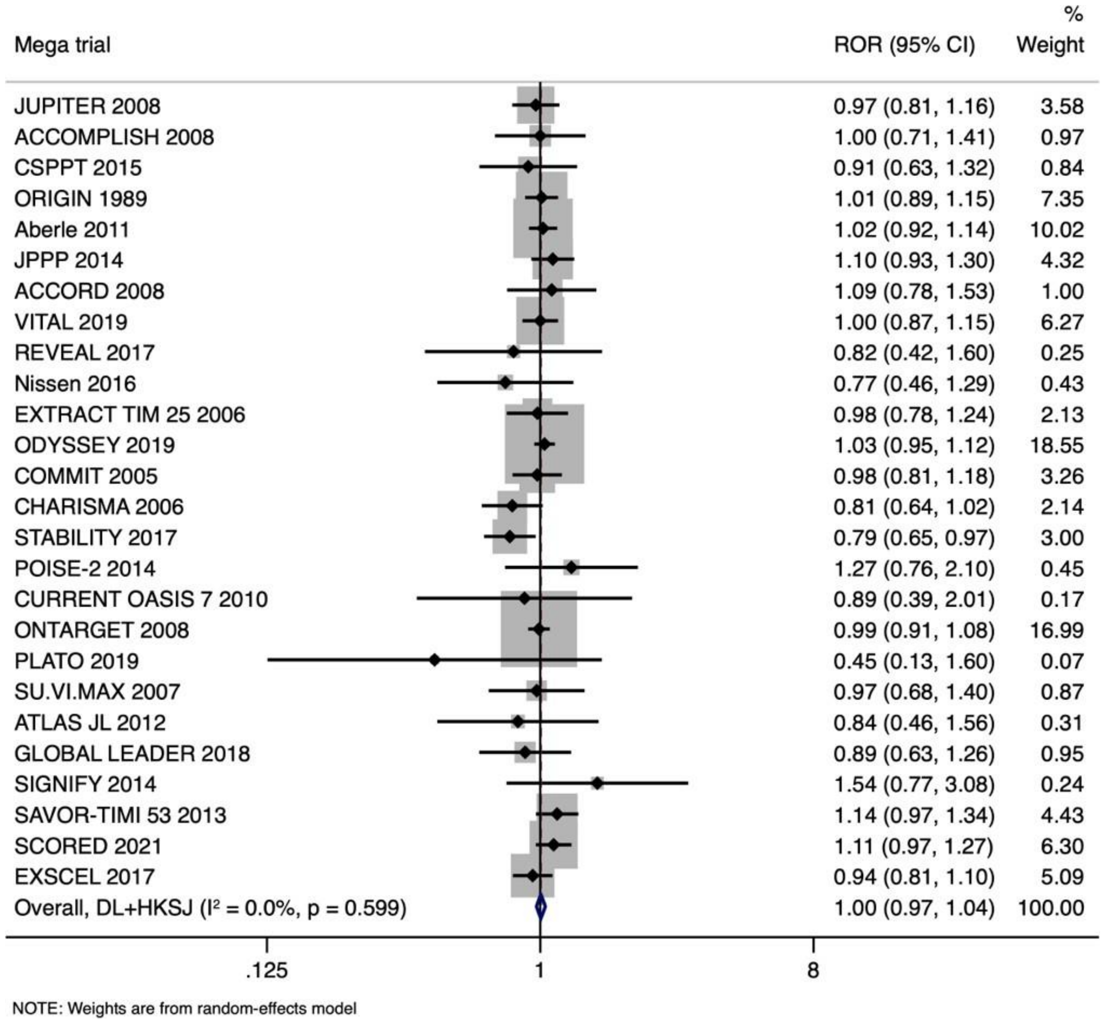

© 2024 Kastrati L et al. *JAMA Network Open.*

eFigure 8. Agreement Between Mega-Trials and Smaller Trials Stratified to Blinding

8A. Primary Outcome

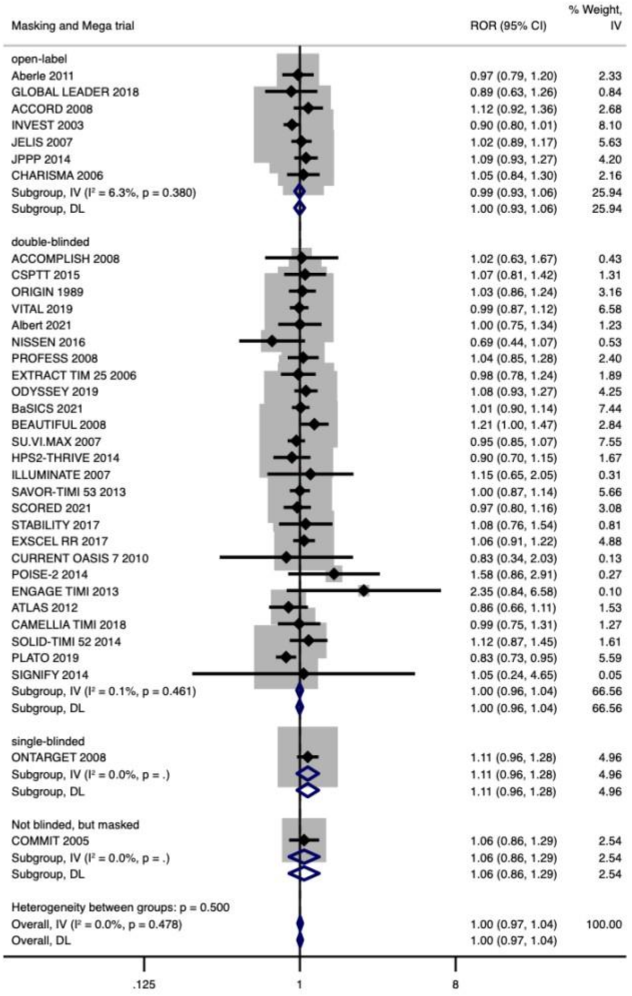

© 2024 Kastrati L et al. *JAMA Network Open.*

8B. All-Cause Mortality

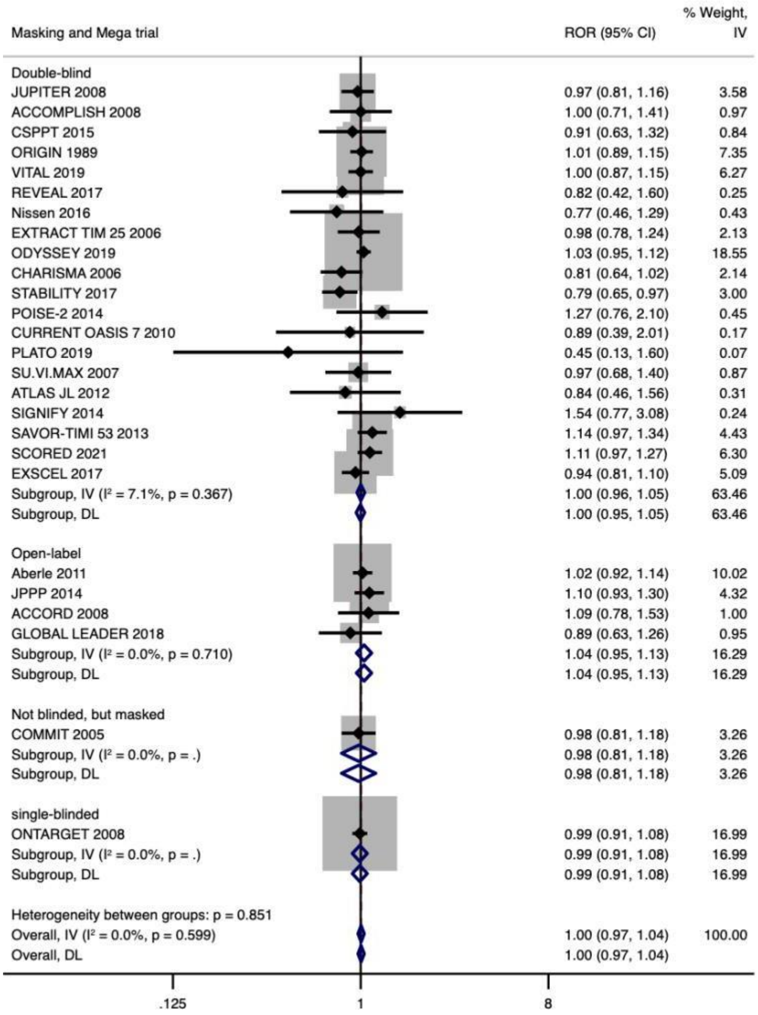

© 2024 Kastrati L et al. *JAMA Network Open.*

eFigure 9. Agreement Between Mega-Trials and Smaller Trials Stratified to Intervention Type

9A. Primary Outcome

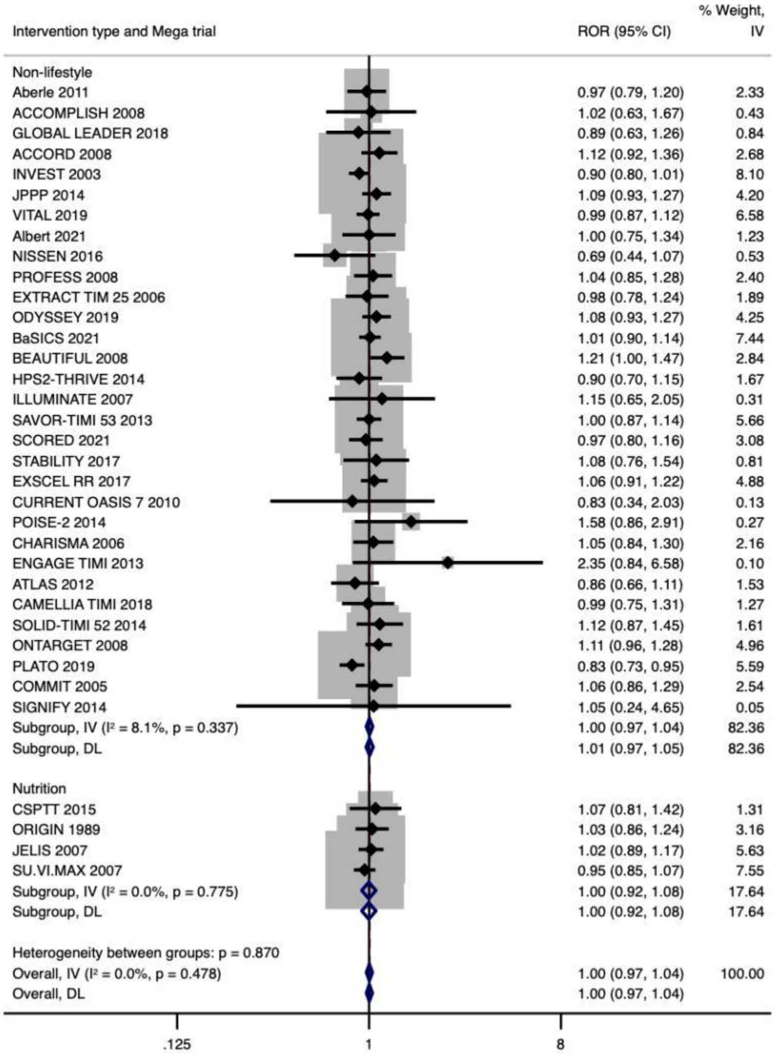

© 2024 Kastrati L et al. *JAMA Network Open.*

9B. All-cause mortality

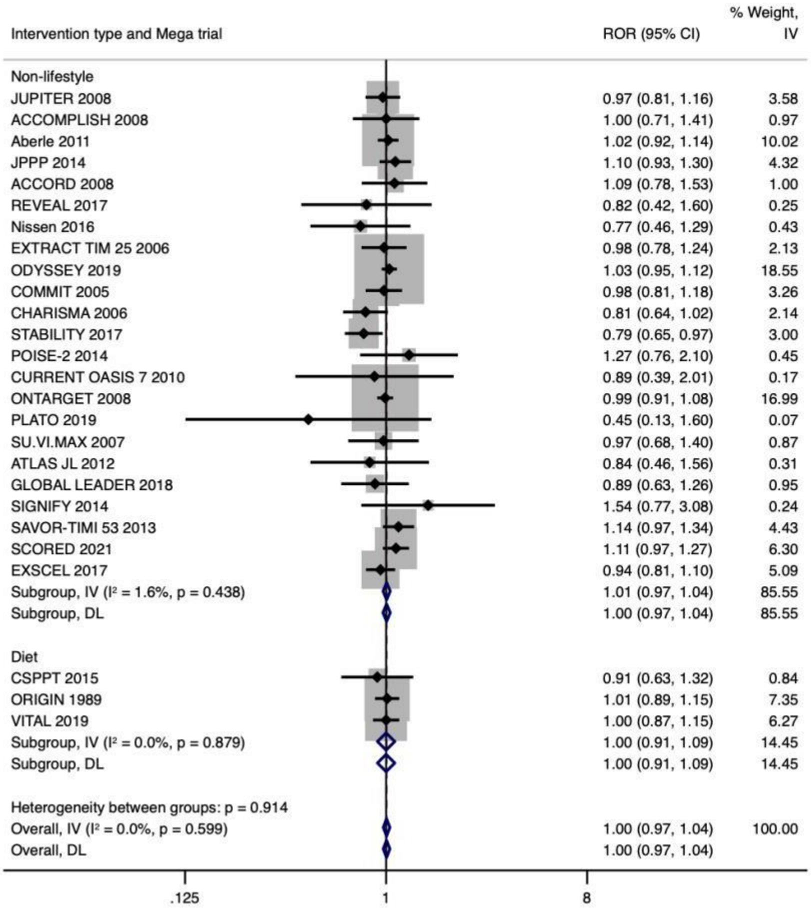

© 2024 Kastrati L et al. *JAMA Network Open.*

eFigure 10. Agreement Between Mega-Trials and Smaller Trials Stratified to Specialty

10A. Primary Outcome

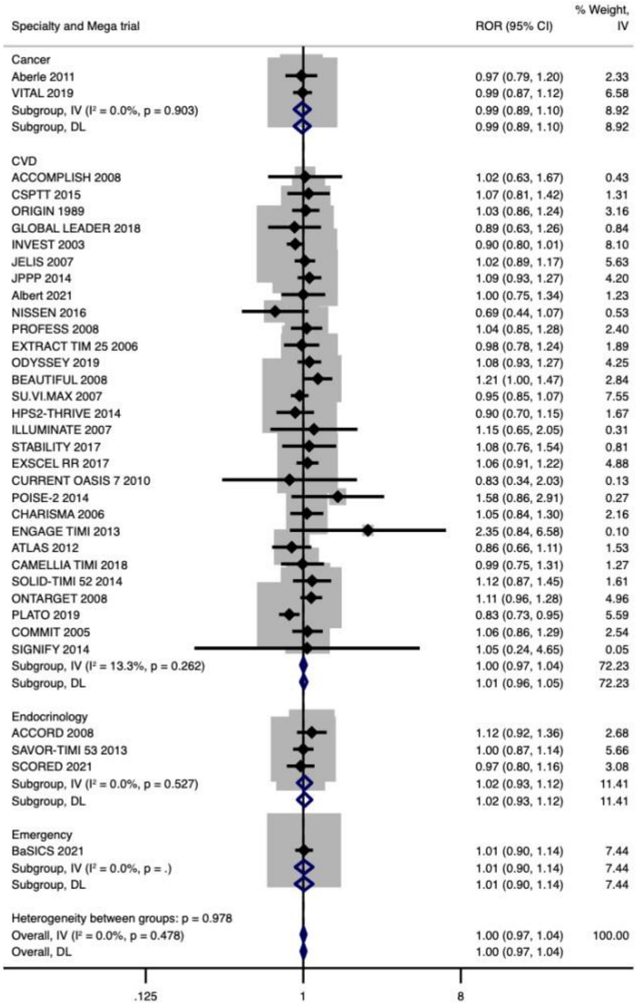

© 2024 Kastrati L et al. *JAMA Network Open.*

10B. All-Cause Mortality

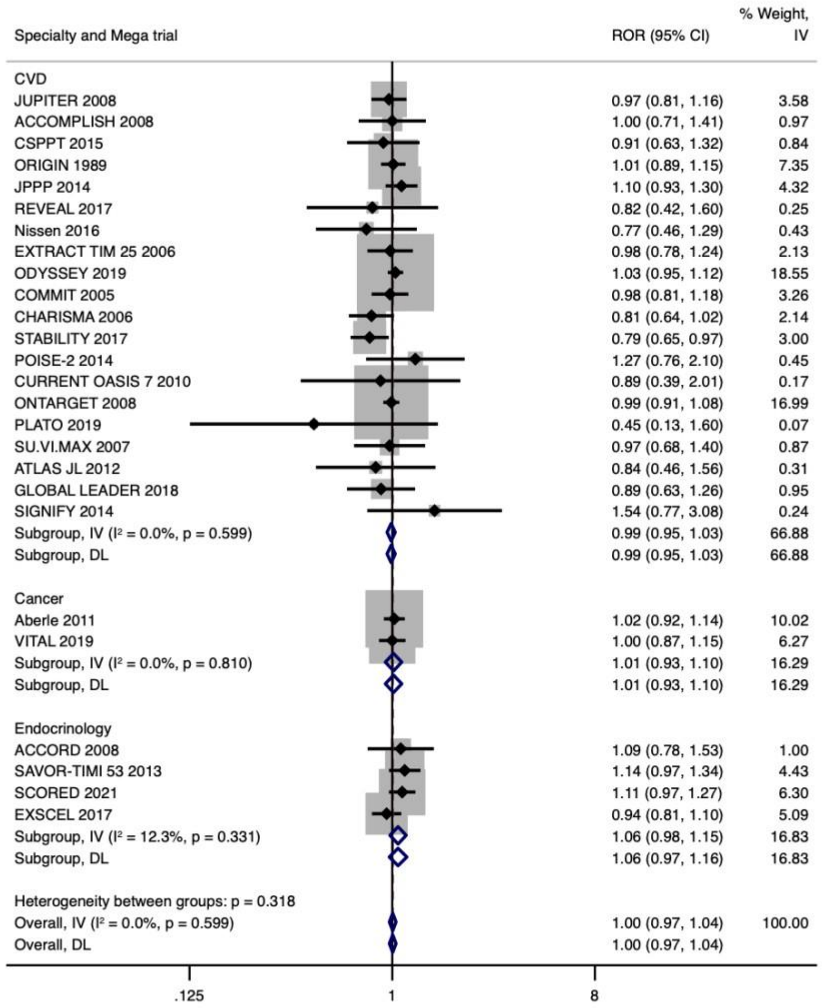

© 2024 Kastrati L et al. *JAMA Network Open.*

eFigure 11. Agreement Between Mega-Trials and Smaller Trials Stratified to Heterogeneity

11A. Primary Outcome

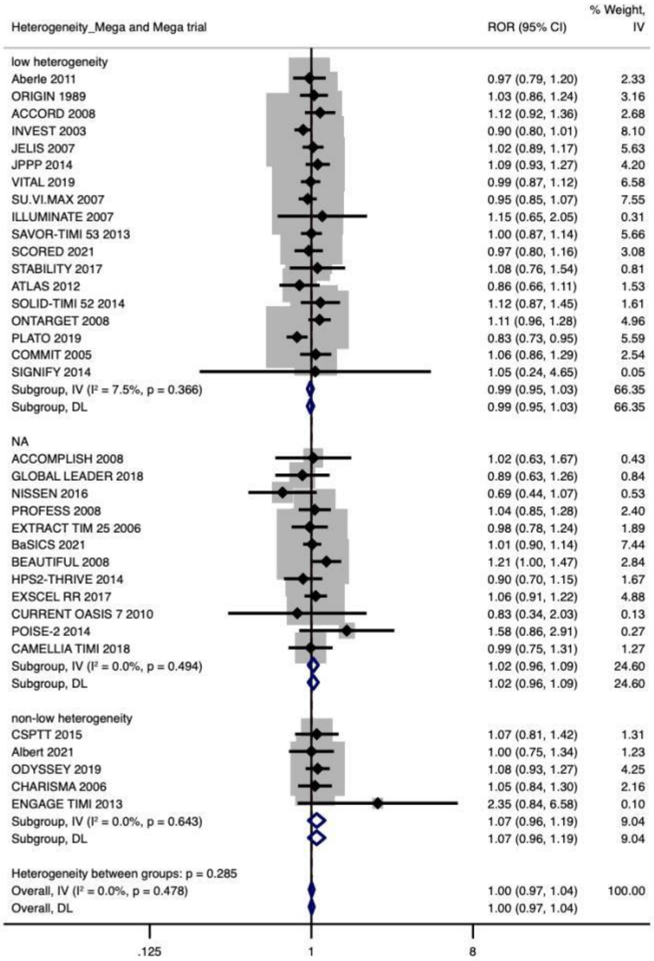

© 2024 Kastrati L et al. *JAMA Network Open.*

11B. All-Cause Mortality

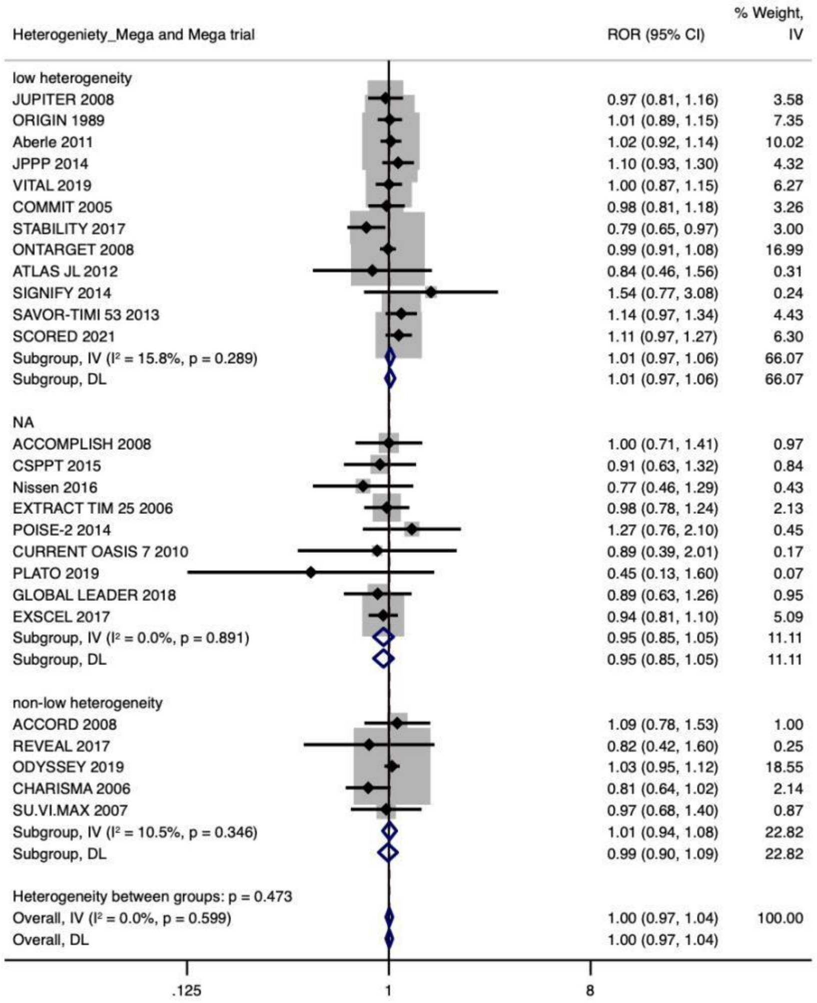

© 2024 Kastrati L et al. *JAMA Network Open.*

eFigure 12. Agreement Between Trials With More Than 30,000 Participants and Smaller Trial for the Primary Outcome

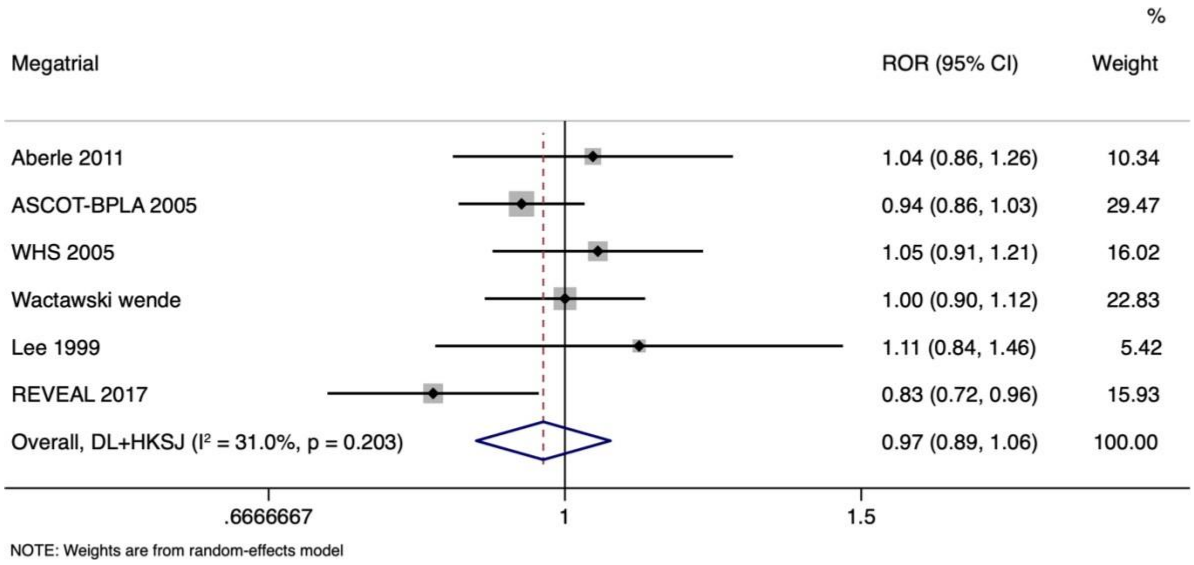

Note* there were only 3 instances for all-cause mortality (ACM) therefore we did not preform this sensitivity analysis for ACM.

© 2024 Kastrati L et al. *JAMA Network Open.*

eFigure 13. Agreement Between Mega-Trials When More Than One Was Present in a Meta-Analysis–Primary Outcome

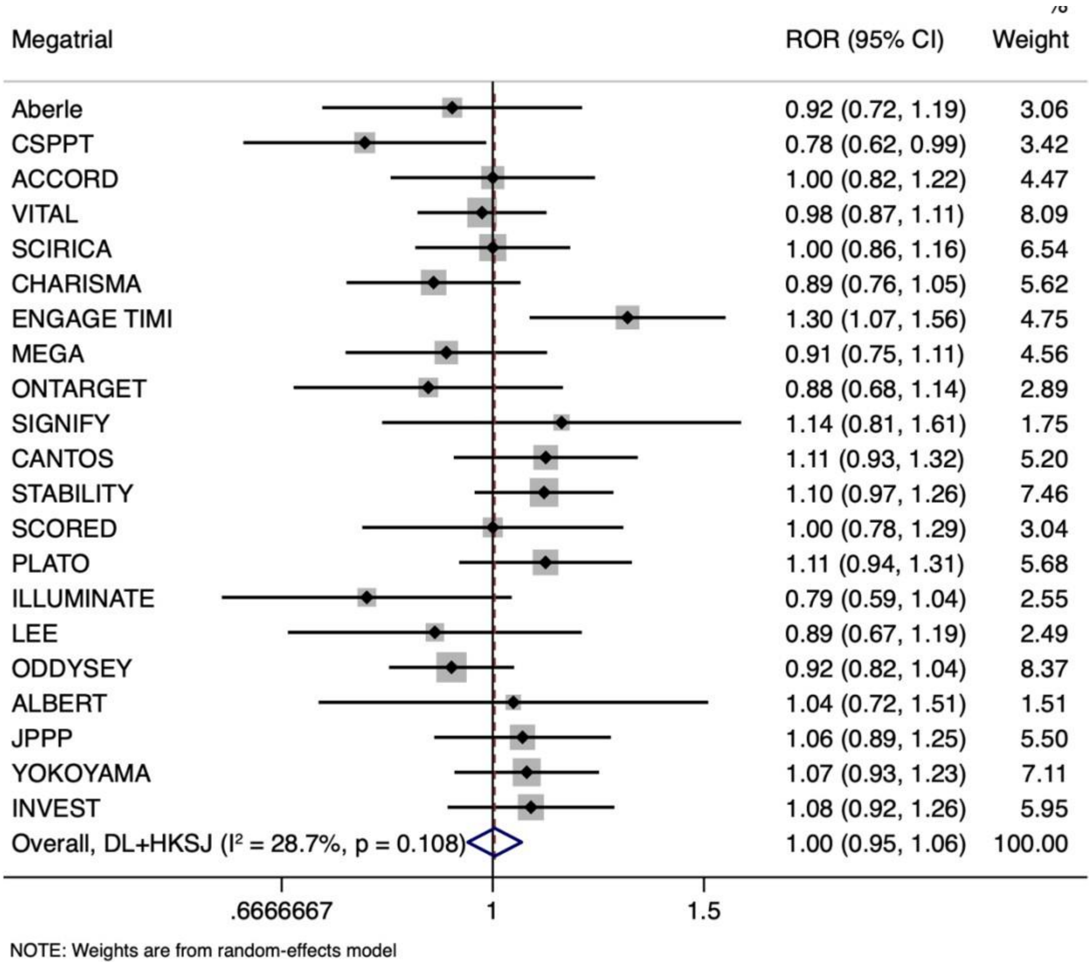

© 2024 Kastrati L et al. *JAMA Network Open.*

eREFERENCES

1. Jamerson, K., et al., *Benazepril plus amlodipine or hydrochlorothiazide for hypertension in high-risk patients.* N Engl J Med, 2008. **359**(23): p. 2417-28.
2. Vranckx, P., et al., *Ticagrelor plus aspirin for 1 month, followed by ticagrelor monotherapy for 23 months vs aspirin plus clopidogrel or ticagrelor for 12 months, followed by aspirin monotherapy for 12 months after implantation of a drug-eluting stent: a multicentre, open-label, randomised superiority trial.* Lancet, 2018. **392**(10151): p. 940-949.
3. Nissen, S.E., et al., *Cardiovascular Safety of Celecoxib, Naproxen, or Ibuprofen for Arthritis.* N Engl J Med, 2016. **375**(26): p. 2519-29.
4. Antman, E.M., et al., *Enoxaparin versus unfractionated heparin with fibrinolysis for ST-elevation myocardial infarction.* N Engl J Med, 2006. **354**(14): p. 1477-88.
5. Fox, K., et al., *Ivabradine for patients with stable coronary artery disease and left-ventricular systolic dysfunction (BEAUTIFUL): a randomised, double-blind, placebo-controlled trial.* Lancet, 2008. **372**(9641): p. 807-16.
6. Landray, M.J., et al., *Effects of extended-release niacin with laropiprant in high-risk patients.* N Engl J Med, 2014. **371**(3): p. 203-12.
7. Barter, P.J., et al., *Effects of torcetrapib in patients at high risk for coronary events.* N Engl J Med, 2007. **357**(21): p. 2109-22.
8. Wallentin, L., et al., *Ticagrelor versus clopidogrel in patients with acute coronary syndromes.* N Engl J Med, 2009. **361**(11): p. 1045-57.
9. Mehta, S.R., et al., *Double-dose versus standard-dose clopidogrel and high-dose versus low-dose aspirin in individuals undergoing percutaneous coronary intervention for acute coronary syndromes (CURRENT-OASIS 7): a randomised factorial trial.* Lancet, 2010. **376**(9748): p. 1233-43.
10. Devereaux, P.J., et al., *Clonidine in patients undergoing noncardiac surgery.* N Engl J Med, 2014. **370**(16): p. 1504-13.
11. Giugliano, R.P., et al., *Edoxaban versus warfarin in patients with atrial fibrillation.* N Engl J Med, 2013. **369**(22): p. 2093-104.
12. O'Donoghue, M.L., et al., *Effect of darapladib on major coronary events after an acute coronary syndrome: the SOLID-TIMI 52 randomized clinical trial.* Jama, 2014. **312**(10): p. 1006-15.
13. Yusuf, S., et al., *Telmisartan, ramipril, or both in patients at high risk for vascular events.* N Engl J Med, 2008. **358**(15): p. 1547-59.
14. Bowman, L., et al., *Effects of Anacetrapib in Patients with Atherosclerotic Vascular Disease.* N Engl J Med, 2017. **377**(13): p. 1217-1227.
15. Gaziano, J.M., et al., *Use of aspirin to reduce risk of initial vascular events in patients at moderate risk of cardiovascular disease (ARRIVE): a randomised, double-blind, placebo-controlled trial.* Lancet, 2018. **392**(10152): p. 1036-1046.
16. Lincoff, A.M., et al., *Evacetrapib and Cardiovascular Outcomes in High-Risk Vascular Disease.* N Engl J Med, 2017. **376**(20): p. 1933-1942.
17. Schwartz, G.G., et al., *Effects of dalcetrapib in patients with a recent acute coronary syndrome.* N Engl J Med, 2012. **367**(22): p. 2089-99.
18. Wiviott, S.D., et al., *Prasugrel versus clopidogrel in patients with acute coronary syndromes.* N Engl J Med, 2007. **357**(20): p. 2001-15.
19. Abtan, J., et al., *Efficacy and Safety of Cangrelor in Preventing Periprocedural Complications in Patients With Stable Angina and Acute Coronary Syndromes Undergoing Percutaneous Coronary Intervention: The CHAMPION PHOENIX Trial.* JACC Cardiovasc Interv, 2016. **9**(18): p. 1905-13.

© 2024 Kastrati L et al. *JAMA Network Open.*

20. Julius, S., et al., *Outcomes in hypertensive patients at high cardiovascular risk treated with regimens based on valsartan or amlodipine: the VALUE randomised trial.* Lancet, 2004. **363**(9426): p. 2022-31.
21. Virtamo, J., et al., *Incidence of cancer and mortality following alpha-tocopherol and beta-carotene supplementation: a postintervention follow-up.* Jama, 2003. **290**(4): p. 476-85.
22. Omenn, G.S., et al., *Effects of a combination of beta carotene and vitamin A on lung cancer and cardiovascular disease.* N Engl J Med, 1996. **334**(18): p. 1150-5.
23. Hennekens, C.H., et al., *Lack of effect of long-term supplementation with beta carotene on the incidence of malignant neoplasms and cardiovascular disease.* N Engl J Med, 1996. **334**(18): p. 1145-9.
24. Goodman, G.E., et al., *The Beta-Carotene and Retinol Efficacy Trial: incidence of lung cancer and cardiovascular disease mortality during 6-year follow-up after stopping beta-carotene and retinol supplements.* J Natl Cancer Inst, 2004. **96**(23): p. 1743-50.
25. *Randomised trial of intravenous atenolol among 16 027 cases of suspected acute myocardial infarction: ISIS-1. First International Study of Infarct Survival Collaborative Group.* Lancet, 1986. **2**(8498): p. 57-66.
26. Taguchi, I., et al., *High-Dose Versus Low-Dose Pitavastatin in Japanese Patients With Stable Coronary Artery Disease (REAL-CAD): A Randomized Superiority Trial.* Circulation, 2018. **137**(19): p. 1997-2009.
27. Armitage, J., et al., *Intensive lowering of LDL cholesterol with 80 mg versus 20 mg simvastatin daily in 12,064 survivors of myocardial infarction: a double-blind randomised trial.* Lancet, 2010. **376**(9753): p. 1658-69.
28. LaRosa, J.C., et al., *Intensive lipid lowering with atorvastatin in patients with stable coronary disease.* N Engl J Med, 2005. **352**(14): p. 1425-35.
29. Sever, P.S., et al., *Prevention of coronary and stroke events with atorvastatin in hypertensive patients who have average or lower-than-average cholesterol concentrations, in the Anglo-Scandinavian Cardiac Outcomes Trial--Lipid Lowering Arm (ASCOT-LLA): a multicentre randomised controlled trial.* Lancet, 2003. **361**(9364): p. 1149-58.
30. *Major outcomes in high-risk hypertensive patients randomized to angiotensin-converting enzyme inhibitor or calcium channel blocker vs diuretic: The Antihypertensive and Lipid-Lowering Treatment to Prevent Heart Attack Trial (ALLHAT).* Jama, 2002. **288**(23): p. 2981-97.
31. LaCroix, A.Z., et al., *Calcium plus vitamin D supplementation and mortality in postmenopausal women: the Women's Health Initiative calcium-vitamin D randomized controlled trial.* J Gerontol A Biol Sci Med Sci, 2009. **64**(5): p. 559-67.
32. Hansson, L., et al., *Effects of intensive blood-pressure lowering and low-dose aspirin in patients with hypertension: principal results of the Hypertension Optimal Treatment (HOT) randomised trial. HOT Study Group.* Lancet, 1998. **351**(9118): p. 1755-62.
33. de Koning, H.J., et al., *Reduced Lung-Cancer Mortality with Volume CT Screening in a Randomized Trial.* N Engl J Med, 2020. **382**(6): p. 503-513.
34. *Dietary supplementation with n-3 polyunsaturated fatty acids and vitamin E after myocardial infarction: results of the GISSI-Prevenzione trial. Gruppo Italiano per lo Studio della Sopravvivenza nell'Infarto miocardico.* Lancet, 1999. **354**(9177): p. 447-55.

© 2024 Kastrati L et al. *JAMA Network Open.*

35. Leppälä, J.M., et al., *Controlled trial of alpha-tocopherol and beta-carotene supplements on stroke incidence and mortality in male smokers.* Arterioscler Thromb Vasc Biol, 2000. **20**(1): p. 230-5.
36. Lee, I.M., et al., *Beta-carotene supplementation and incidence of cancer and cardiovascular disease: the Women's Health Study.* J Natl Cancer Inst, 1999. **91**(24): p. 2102-6.
37. McNeil, J.J., et al., *Effect of Aspirin on All-Cause Mortality in the Healthy Elderly.* N Engl J Med, 2018. **379**(16): p. 1519-1528.
38. Ridker, P.M., et al., *A randomized trial of low-dose aspirin in the primary prevention of cardiovascular disease in women.* N Engl J Med, 2005. **352**(13): p. 1293-304.
39. Hansson, L., et al., *Effect of angiotensin-converting-enzyme inhibition compared with conventional therapy on cardiovascular morbidity and mortality in hypertension: the Captopril Prevention Project (CAPPP) randomised trial.* Lancet, 1999. **353**(9153): p. 611-6.
40. Dahlöf, B., et al., *Prevention of cardiovascular events with an antihypertensive regimen of amlodipine adding perindopril as required versus atenolol adding bendroflumethiazide as required, in the Anglo-Scandinavian Cardiac Outcomes Trial-Blood Pressure Lowering Arm (ASCOT-BPLA): a multicentre randomised controlled trial.* Lancet, 2005. **366**(9489): p. 895-906.
41. Black, H.R., et al., *Principal results of the Controlled Onset Verapamil Investigation of Cardiovascular End Points (CONVINCE) trial.* Jama, 2003. **289**(16): p. 2073-82.
42. Hansson, L., et al., *Randomised trial of effects of calcium antagonists compared with diuretics and beta-blockers on cardiovascular morbidity and mortality in hypertension: the Nordic Diltiazem (NORDIL) study.* Lancet, 2000. **356**(9227): p. 359-65.
43. Patel, A., et al., *Intensive blood glucose control and vascular outcomes in patients with type 2 diabetes.* N Engl J Med, 2008. **358**(24): p. 2560-72.
44. Wactawski-Wende, J., et al., *Calcium plus vitamin D supplementation and the risk of colorectal cancer.* N Engl J Med, 2006. **354**(7): p. 684-96.
45. Cannon, C.P., et al., *Ezetimibe Added to Statin Therapy after Acute Coronary Syndromes.* N Engl J Med, 2015. **372**(25): p. 2387-97.
46. Sabatine, M.S., et al., *Evolocumab and Clinical Outcomes in Patients with Cardiovascular Disease.* N Engl J Med, 2017. **376**(18): p. 1713-1722.
47. Green, J.B., et al., *Effect of Sitagliptin on Cardiovascular Outcomes in Type 2 Diabetes.* N Engl J Med, 2015. **373**(3): p. 232-42.
48. Nicholls, S.J., et al., *Effect of High-Dose Omega-3 Fatty Acids vs Corn Oil on Major Adverse Cardiovascular Events in Patients at High Cardiovascular Risk: The STRENGTH Randomized Clinical Trial.* Jama, 2020. **324**(22): p. 2268-2280.
49. *MRC/BHF Heart Protection Study of antioxidant vitamin supplementation in 20,536 high-risk individuals: a randomised placebo-controlled trial.* Lancet, 2002. **360**(9326): p. 23-33.
50. *MRC/BHF Heart Protection Study of cholesterol lowering with simvastatin in 20,536 high-risk individuals: a randomised placebo-controlled trial.* Lancet, 2002. **360**(9326): p. 7-22.
51. Sesso, H.D., et al., *Effect of cocoa flavanol supplementation for the prevention of cardiovascular disease events: the COcoa Supplement and Multivitamin Outcomes Study (COSMOS) randomized clinical trial.* Am J Clin Nutr, 2022. **115**(6): p. 1490-1500.
52. Oldenburg, C.E., et al., *Neonatal azithromycin administration for prevention of infant mortality.* NEJM Evid, 2022. **1**(4): p. EVIDoa2100054.

© 2024 Kastrati L et al. *JAMA Network Open.*

53. Granger, C.B., et al., *Apixaban versus warfarin in patients with atrial fibrillation.* N Engl J Med, 2011. **365**(11): p. 981-92.
54. Bhatt, D.L., et al., *Ticagrelor in patients with diabetes and stable coronary artery disease with a history of previous percutaneous coronary intervention (THEMIS-PCI): a phase 3, placebo-controlled, randomised trial.* Lancet, 2019. **394**(10204): p. 1169-1180.
55. Wallentin, L., et al., *Efficacy and safety of dabigatran compared with warfarin at different levels of international normalised ratio control for stroke prevention in atrial fibrillation: an analysis of the RE-LY trial.* Lancet, 2010. **376**(9745): p. 975-83.
56. Johnston, S.C., et al., *Ticagrelor versus Aspirin in Acute Stroke or Transient Ischemic Attack.* N Engl J Med, 2016. **375**(1): p. 35-43.
57. Steg, P.G., et al., *Anticoagulation with otamixaban and ischemic events in non-ST-segment elevation acute coronary syndromes: the TAO randomized clinical trial.* Jama, 2013. **310**(11): p. 1145-55.
58. Johnston, S.C., et al., *Ticagrelor and Aspirin or Aspirin Alone in Acute Ischemic Stroke or TIA.* N Engl J Med, 2020. **383**(3): p. 207-217.
59. Lipson, D.A., et al., *Once-Daily Single-Inhaler Triple versus Dual Therapy in Patients with COPD.* N Engl J Med, 2018. **378**(18): p. 1671-1680.
60. Wise, R.A., et al., *Tiotropium Respimat inhaler and the risk of death in COPD.* N Engl J Med, 2013. **369**(16): p. 1491-501.
61. Nissen, S.E., et al., *Bempedoic Acid for Primary Prevention of Cardiovascular Events in Statin-Intolerant Patients.* Jama, 2023. **330**(2): p. 131-140.
62. Stempel, D.A., et al., *Serious Asthma Events with Fluticasone plus Salmeterol versus Fluticasone Alone.* N Engl J Med, 2016. **374**(19): p. 1822-30.
63. Vestbo, J., et al., *Fluticasone furoate and vilanterol and survival in chronic obstructive pulmonary disease with heightened cardiovascular risk (SUMMIT): a double-blind randomised controlled trial.* Lancet, 2016. **387**(10030): p. 1817-26.
64. Spyropoulos, A.C., et al., *Rivaroxaban for Thromboprophylaxis after Hospitalization for Medical Illness.* N Engl J Med, 2018. **379**(12): p. 1118-1127.
65. Vogel, V.G., et al., *Effects of tamoxifen vs raloxifene on the risk of developing invasive breast cancer and other disease outcomes: the NSABP Study of Tamoxifen and Raloxifene (STAR) P-2 trial.* Jama, 2006. **295**(23): p. 2727-41.
66. Hiatt, W.R., et al., *Ticagrelor versus Clopidogrel in Symptomatic Peripheral Artery Disease.* N Engl J Med, 2017. **376**(1): p. 32-40.
67. Peters, S.P., et al., *Serious Asthma Events with Budesonide plus Formoterol vs. Budesonide Alone.* N Engl J Med, 2016. **375**(9): p. 850-60.
68. Lippman, S.M., et al., *Effect of selenium and vitamin E on risk of prostate cancer and other cancers: the Selenium and Vitamin E Cancer Prevention Trial (SELECT).* Jama, 2009. **301**(1): p. 39-51.
69. Barrett-Connor, E., et al., *Effects of raloxifene on cardiovascular events and breast cancer in postmenopausal women.* N Engl J Med, 2006. **355**(2): p. 125-37.
70. Christen, W.G., et al., *Age-related cataract in men in the selenium and vitamin e cancer prevention trial eye endpoints study: a randomized clinical trial.* JAMA Ophthalmol, 2015. **133**(1): p. 17-24.
71. James, W.P., et al., *Effect of sibutramine on cardiovascular outcomes in overweight and obese subjects.* N Engl J Med, 2010. **363**(10): p. 905-17.
72. *Effect of early tranexamic acid administration on mortality, hysterectomy, and other morbidities in women with post-partum haemorrhage (WOMAN): an international, randomised, double-blind, placebo-controlled trial.* Lancet, 2017. **389**(10084): p. 2105-2116.

© 2024 Kastrati L et al. *JAMA Network Open.*

73. Chandramohan, D., et al., *Effect of Adding Azithromycin to Seasonal Malaria Chemoprevention.* N Engl J Med, 2019. **380**(23): p. 2197-2206.
74. *Effects of tranexamic acid on death, disability, vascular occlusive events and other morbidities in patients with acute traumatic brain injury (CRASH-3): a randomised, placebo-controlled trial.* Lancet, 2019. **394**(10210): p. 1713-1723.
75. Shakur, H., et al., *Effects of tranexamic acid on death, vascular occlusive events, and blood transfusion in trauma patients with significant haemorrhage (CRASH-2): a randomised, placebo-controlled trial.* Lancet, 2010. **376**(9734): p. 23-32.
76. Dumbleton, J.S., et al., *The Helicobacter Eradication Aspirin Trial (HEAT): A Large Simple Randomised Controlled Trial Using Novel Methodology in Primary Care.* EBioMedicine, 2015. **2**(9): p. 1200-4.
77. *Effects of a high-dose 24-h infusion of tranexamic acid on death and thromboembolic events in patients with acute gastrointestinal bleeding (HALT-IT): an international randomised, double-blind, placebo-controlled trial.* Lancet, 2020. **395**(10241): p. 1927-1936.
78. Brunvoll, S.H., et al., *Prevention of covid-19 and other acute respiratory infections with cod liver oil supplementation, a low dose vitamin D supplement: quadruple blinded, randomised placebo controlled trial.* Bmj, 2022. **378**: p. e071245.
79. Topol, E.J., et al., *Rimonabant for prevention of cardiovascular events (CRESCENDO): a randomised, multicentre, placebo-controlled trial.* Lancet, 2010. **376**(9740): p. 517-23.
80. Stiell, I.G., et al., *Early versus later rhythm analysis in patients with out-of-hospital cardiac arrest.* N Engl J Med, 2011. **365**(9): p. 787-97.
81. Das Pradhan, A., et al., *Triglyceride Lowering with Pemafibrate to Reduce Cardiovascular Risk.* N Engl J Med, 2022. **387**(21): p. 1923-1934.
82. Sazawal, S., et al., *Efficacy of chlorhexidine application to umbilical cord on neonatal mortality in Pemba, Tanzania: a community-based randomised controlled trial.* Lancet Glob Health, 2016. **4**(11): p. e837-e844.
83. Ishani, A., et al., *Chlorthalidone vs. Hydrochlorothiazide for Hypertension-Cardiovascular Events.* N Engl J Med, 2022. **387**(26): p. 2401-2410.
84. Strom, B.L., et al., *Comparative mortality associated with ziprasidone and olanzapine in real-world use among 18,154 patients with schizophrenia: The Ziprasidone Observational Study of Cardiac Outcomes (ZODIAC).* Am J Psychiatry, 2011. **168**(2): p. 193-201.
85. Bretthauer, M., et al., *Population-Based Colonoscopy Screening for Colorectal Cancer: A Randomized Clinical Trial.* JAMA Intern Med, 2016. **176**(7): p. 894-902.
86. Hermida, R.C., et al., *Bedtime hypertension treatment improves cardiovascular risk reduction: the Hygia Chronotherapy Trial.* Eur Heart J, 2020. **41**(48): p. 4565-4576.
87. Hansen, A.W., et al., *Effect of a Web-based intervention to promote physical activity and improve health among physically inactive adults: a population-based randomized controlled trial.* J Med Internet Res, 2012. **14**(5): p. e145.
88. Patel, M.R., et al., *Rivaroxaban versus warfarin in nonvalvular atrial fibrillation.* N Engl J Med, 2011. **365**(10): p. 883-91.

© 2024 Kastrati L et al. *JAMA Network Open.*
