# Supplementary material for: Agreement Between Mega-Trials and Smaller Trials: A Systematic Review and Meta-Research Analysis
Source: JAMA Netw Open. 2024 Sep 6;7(9):e2432296. doi: 10.1001/jamanetworkopen.2024.32296 (PMC11380108; doi:10.1001/jamanetworkopen.2024.32296)
Supplement: Supplement 2. — Data Sharing Statement [file jamanetwopen-e2432296-s002.pdf]

Data Sharing Statement

Kastrati. Agreement Between Mega-Trials and Smaller Trials. *JAMA Netw Open*. Published September 06, 2024. doi:10.1001/jamanetworkopen.2024.32296

Data
Data available: Yes
Data types: Data (not involving human participants)
How to access data: Data can become available upon request to jioannid@stanford.edu
When available: With publication

Supporting Documents
Document types: None

Additional Information
Who can access the data: Researchers whose proposed use of the data has been approved
Types of analyses: for any scientifically reasonable purpose
Mechanisms of data availability: after approval of a proposal
